# Targeting the Adenosine‐Mediated Metabolic Immune Checkpoint with Engineered Probiotic for Enhanced Chemo‐Immunotherapy

**DOI:** 10.1002/advs.202411813

**Published:** 2025-02-22

**Authors:** Jinhui Wang, Jing Wang, Zhijie Yu, Hongyu Wen, Chensi Zhao, Jiayong Zhong, Chuanle Xiao, Yingqiu Li, Jianqiao Xu, Jinquan Wang, Zong‐Wan Mao, Wei Xia

**Affiliations:** ^1^ MOE Key Laboratory of Bioinorganic and Synthetic Chemistry IGCME School of Chemistry Sun Yat‐Sen University Guangzhou 510006 China; ^2^ Guangdong Key Laboratory of Advanced Drug Delivery School of Bioscience and Biopharmaceutics Guangdong Pharmaceutical University Guangzhou 510006 China; ^3^ State Key Laboratory of Ophthalmology Zhongshan Ophthalmic Center Guangdong Provincial Key Laboratory of Ophthalmology and Visual Science Sun Yat‐sen University Guangzhou 510060 China; ^4^ State Key Laboratory of Biocontrol School of Life Sciences Sun Yat‐sen University Guangzhou 510006 China

**Keywords:** adenosine deaminase, chemo‐immunotherapy, engineered probiotic, metabolic modulation, tumor microenvironment

## Abstract

Immunotherapy has revolutionized cancer treatment by leveraging the patient's immune system, yet its efficacy is often hampered by the immunosuppressive tumor microenvironment (TME). Adenosine, a key player in this milieu, suppresses immune cell activity via cAMP signaling. Here, an innovative strategy to remodel the TME using a genetically engineered strain of *Escherichia coli* Nissle 1917 that expresses adenosine deaminase on its surface under hypoxic conditions is presented. This engineered probiotic targets tumors, converts immunosuppressive adenosine to inosine, and enhances anti‐tumor immune responses. In vivo, the engineered probiotic significantly improved immune cell infiltration and demonstrated synergistic effects with low‐dose doxorubicin in both subcutaneous and orthotopic mouse colorectal cancer model. Furthermore, the engineered probiotic modulated the TME, promoting a shift from M2‐like to M1‐like macrophages and increasing effector T cell populations. These findings highlight the potential of using engineered probiotics for metabolic modulation of the TME, offering a novel approach for enhancing cancer immunotherapy.

## Graphical abstract

This study presents engineered probiotics that convert immunosuppressive adenosine into inosine in tumors, alleviating immunosuppression and enhancing antitumor immunity. Demonstrating efficacy in murine ectopic and orthotopic tumor models, this approach offers a promising probiotic‐based cancer immunotherapy strategy, highlighting the potential of microbial therapeutics in oncology.

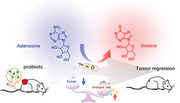

## 1 Introduction

Immunotherapy has emerged as a promising approach for cancer treatment by harnessing the patient's own immune system. This therapeutic strategy has shown remarkable success in various cancer types and improved survival rates for patients.^[^ ^1^, ^2^, ^3^ ^]^ However, the immunotherapeutic efficacy can be comprised by the immunosuppressive tumor environment.^[^ ^4^, ^5^, ^6^ ^]^ The adenosine metabolite in tumor milieu plays important roles in the establishment of such an immunosuppressive microenvironment. In tumor, the released ATP from damaged cell was converted to adenosine by the cell surface ecto‐nucleotidases, such as CD39 and CD73, resulting in the accumulation of extracellular adenosine.^[^ ^7^, ^8^ ^]^ The extracellular adenosine then binds to the adenosine receptors on immune cells and activates downstream cyclic adenosine 3′,5′‐monophosphate (cAMP) signaling, which inhibits the proliferation, survival, activation of immune cells, and facilitates immune evasion by tumors.^[^ ^9^ ^]^ Therefore, targeting the adenosinergic pathway is a plausible strategy to remodel the immunosuppressive tumor microenvironment (TME), and enhancing cancer immunotherapy.^[^ ^10^, ^11^, ^12^, ^13^, ^14^ ^]^ Recently, several studies demonstrated that a repurposed drug, PEGylated adenosine deaminase (PEG‐ADA) could deplete extracellular adenosine by enzymatic converting adenosine to inosine, which subsequently stimulates T cell proliferation, promotes tumor infiltration and inhibits tumor growth, indicative of the potential of adenosine deaminase (ADA) as a promising therapeutic enzyme for cancer treatment.^[^ ^15^, ^16^, ^17^ ^]^ However, relatively high concentration of PEG‐ADA must be administrated due to the lack of tumor specificity, resulting in low therapeutic efficacy and even side effect.^[^ ^18^ ^]^ Therefore, alternative means to locally and continuously increase the effective concentration of ADA in tumors are highly desirable.

Bacteria have attracted increasing attention in cancer treatment, benefiting from their inherent features, such as self‐propelled motility, hypoxia tropism, and preferential tumor colonization.^[^ ^19^ ^]^ Furthermore, the bacteria could also trigger anti‐tumor immune responses due to their intrinsic immunostimulatory nature. Another attractive characteristic of bacteria is their capability to be modified chemically and genetically. In the past few years, a variety of attenuated pathogenic bacteria and probiotics, such as *Listeria*, *Clostridium*, *Salmonella* and *Escherichia coli* Nissle 1917 (*Ec*N) have been engineered and extensively used in cancer treatments.^[^ ^20^, ^21^, ^22^, ^23^, ^24^, ^25^ ^]^ Majority of these studies focused on the use of bacteria to deliver and release chemical or biological payloads.^[^ ^26^, ^27^, ^28^, ^29^, ^30^, ^31^, ^32^ ^]^ However, engineered bacteria for metabolic modulation of immunosuppressive TME has been rarely reported.^[^ ^33^ ^]^

In this study, we designed and constructed a therapeutic *Ec*N strain, which is capable of reshape the immunosuppressive TME through extracellular adenosine depletion. In this *Ec*N strain, the ADA gene and the gene encoding a bacterial autotransporter domain (AIDA‐I) were fused and were expressed under the control of a hypoxia‐responsive *fnrS* promoter (P*_fnrS_*). The constructed *Ec*N strains (*Ec*N_ADA) spatiotemporally targets tumors, expresses and displays ADA on bacterial surface under hypoxic tumor condition. The surface‐exposed ADA then converts extracellular adenosine into inosine in situ to alleviate the immunosuppressive effects, thereby leading to intratumoral immune cells activation. In a subcutaneous colorectal cancer (CRC) mouse model, we demonstrated that *Ec*N_ADA strain substantially improved immune cell infiltration and had marked synergistic effects with low dose of chemotherapeutic in the clearance of solid tumors. Furthermore, the *Ec*N_ADA strain also ameliorated intestinal inflammation and tumor growth in an orthotopic CRC mouse model. The results demonstrate the potential to utilize synthetic biology technique for TME metabolite modulation. We anticipate that the bioengineered probiotics *Ec*N_ADA could provide a unique strategy for synergizing chemotherapeutics for combinational cancer treatment.

## 2 Results and Discussion

### 2.1 Adenosine Deaminase Reverses Adenosine‐Mediated Immunosuppression of Immune Cells In Vitro

ADA is one of the key enzymes involved in purine metabolism. There are mainly two isoforms in humans: a predominantly intracellular monomeric ADA1 (35 kDa) and a serum dimeric ADA2 (100 kDa).^[^ ^34^ ^]^ Since the protein with lower molecular weight is more compatible for bacterial surface display, the monomeric ADA1 was chosen for further studies. In vitro catalytic results showed that recombinant human ADA1 had a high activity toward adenosine with a *K*_cat_ value of 143 and 138 s^−1^ at pH 7.4 and 6.8, respectively (Figure S1, Supporting Information), which are consistent with previous studies.^[^ ^35^ ^]^ Extensive investigations have demonstrated that high concentrations of extracellular adenosine inhibit the activation and effector functions of various immune cell populations.^[^ ^36^, ^37^, ^38^ ^]^ Therefore, we subsequently sought to investigate whether ADA could reverse adenosine‐mediated immunosuppression of CD8^+^ T cells and macrophages in vitro.

**Figure 1:**
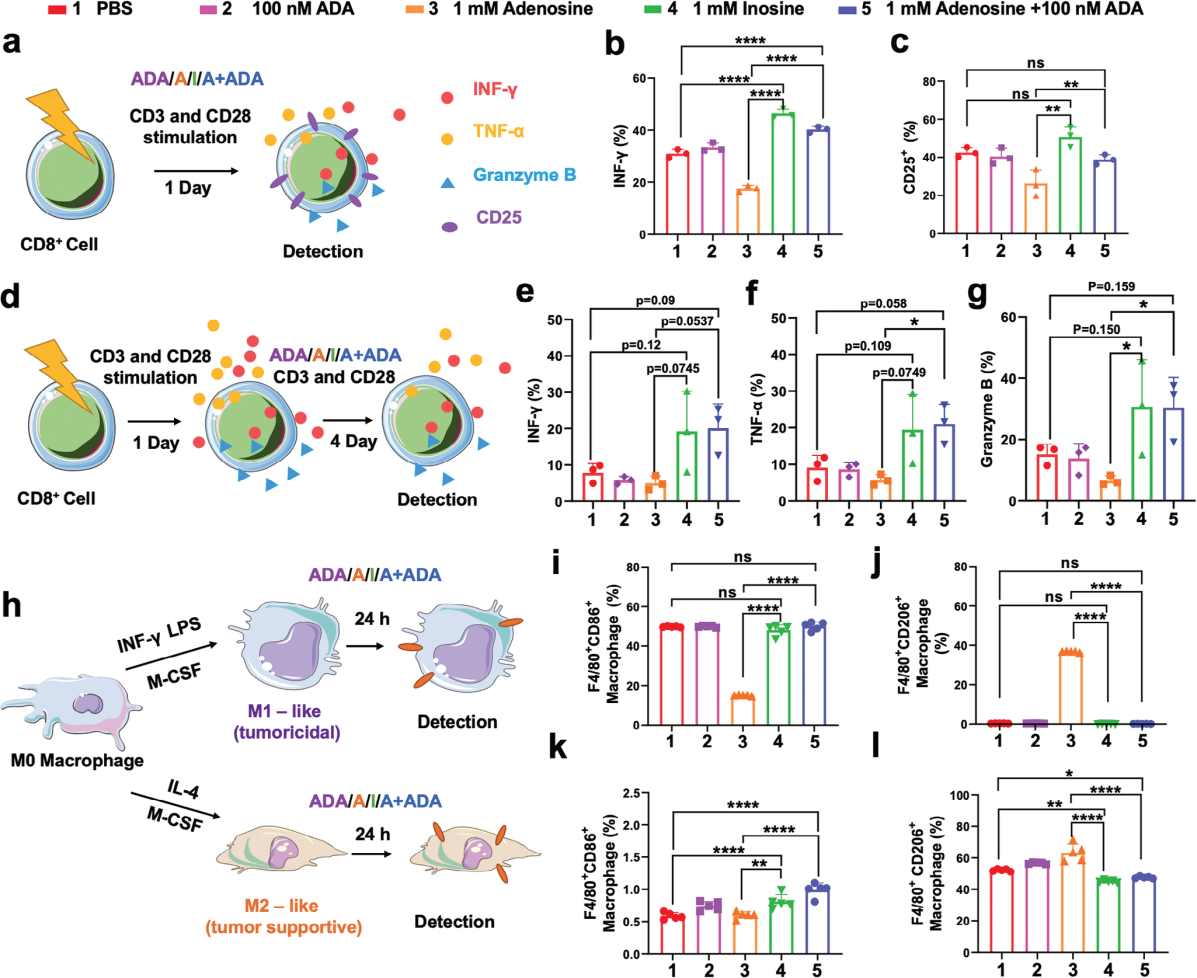
Adenosine deaminase alleviates adenosine‐mediated suppression of immune cells in vitro. a) Schematic illustration of the impacts of purine nucleotides on the activation of CD8^+^ T cells. b,c) Flow cytometry analysis of intracellular INF‐γ and cell surface CD25^+^ marker expression levels in CD8^+^ T cells after the indicated treatments. d) Schematic illustration of purine nucleotides as alternative carbon source to sustain the function of effector CD8^+^ T cells in glucose‐depleted medium. e–g) Flow cytometry analysis of the persistent expression of INF‐γ, TNF‐α, and Granzyme B in effector CD8^+^ T cells after the indicated treatments in glucose‐depleted condition. Error bars indicate standard deviation of three independent experiments (n = 3). h) Schematic diagram illustration of the impacts of purine nucleotides on phenotype switch of M1 and M2 macrophages. Flow cytometry analysis of the expression levels of F4/80, CD86 and CD206 markers on M1‐like i,j) and M2‐like macrophages k,l) after the indicated treatments. Error bars indicate standard deviation of five independent experiments (n = 5). Differences between groups were assessed using one‐way ANOVAs, followed by Bonferroni's correction for multiple comparisons. Significance levels were indicated as follows: *p < 0.05, **p < 0.01, ***p < 0.001, ****p < 0.0001.

To investigate the impact of ADA on CD8^+^ T cells activation, isolated mouse spleen CD8^+^ T cells were stimulated with anti‐CD3 and anti‐CD28 antibodies in the presence of PBS, 100 nM ADA, 1 mM adenosine, 1 mM inosine, and 1 mM adenosine + 100 nM ADA (**Figure** **1**a). After 24 h incubation, flow cytometry analysis was applied to access IFN‐γ and CD25 activation marker in CD8^+^ T cells. As shown in Figure 1b,c, and Figure S2 (Supporting Information), 100 nM ADA alone exhibited negligible effects on CD8^+^ T cells activation. Whereas, CD8^+^ T cells activation was significantly suppressed in the presence of 1 mM adenosine, as evidenced by substantially lower CD25 and IFN‐γ expression compared with the control group. Conversely, 1 mM inosine enhanced the activation of CD8^+^ T cells, leading to elevated IFN‐γ levels. Administration of 1 mM adenosine + 100 nM ADA also resulted in augmented IFN‐γ production comparable to the inosine‐treated group, suggesting that 100 nM ADA efficiently catalyzed the conversion of 1 mM adenosine to inosine. Moreover, previous study have indicated that inosine could serve as an alternative carbon source, supporting the survival and function of effector CD8^+^ T cells under glucose‐depleted conditions.^[^ ^39^ ^]^ Therefore, we subsequently examined whether the enzymatically catalyzed product by ADA could similarly sustain the function of effector CD8^+^ T cells. After activation with anti‐CD3 and anti‐CD28 antibodies for 24 h, the effector CD8^+^ T cells were transferred into glucose‐free medium and cultured for an additional four days under various treatments, including PBS buffer, 100 nM ADA, 1 mM adenosine, 1 mM inosine, and 1 mM adenosine + 100 nM ADA (Figure 1d). Flow cytometry data showed that 100 nM ADA or 1 mM adenosine treatment had similar expression levels of IFN‐γ, TNF‐α and Granzyme B in CD8^+^ T cells compared with the control group. Although not statistically significant, a clear trend toward higher expression of IFN‐γ, TNF‐α and Granzyme B was observed in the presence of 1 mM inosine and 1 mM adenosine + 100 nM ADA (Figure 1e–g; Figure S3, Supporting Information). These results indicated that the enzymatically generated inosine by ADA had the capacity to sustain the function of effector CD8^+^ T cells in the glucose‐depleted conditions.

Adenosine receptor activation promotes the macrophages switching from a pro‐inflammatory M1 to an anti‐inflammatory M2 phenotype. Therefore, we further examined the impact of ADA on the adenosine‐mediated M1‐M2 macrophage phenotype switch. The mouse bone‐marrow‐derived macrophages (BMDMs) were isolated and polarized into M1 and M2 macrophages. The obtained M1‐like macrophages were subsequently incubated with PBS, 100 nm ADA, 1 mM adenosine, 1 mM inosine, or 1 mM adenosine + 100 nm ADA for 24 h (Figure 1h). Exposure to 1 mM adenosine induced a transition in macrophage phenotype from M1 to M2, characterized by a significant reduction in CD86 levels and elevation of CD206 biomarker expression. In contrast, 1 mM adenosine + 100 nm ADA treatment has no effects on M1 phenotype (Figure 1i, j; Figure S4, Supporting Information). For M2‐like macrophages, 1 mM inosine and 1 mM adenosine + 100 nm ADA treatment resulted in an obvious increase of CD80 levels and reduction of CD206, indicative of a distinct shift toward pro‐inflammatory M1 phenotype (Figure 1k,l; Figure S5, Supporting Information). Collectedly, all these results demonstrated that ADA could effectively catalyzed adenosine into inosine, reversing the adenosine‐mediated immunosuppression on CD8^+^ T cells and macrophages in vitro.

### 2.2 Peritumoral Injection of Adenosine Deaminase Promotes Immune Cell Infiltration into Solid Tumor

**Figure 2:**
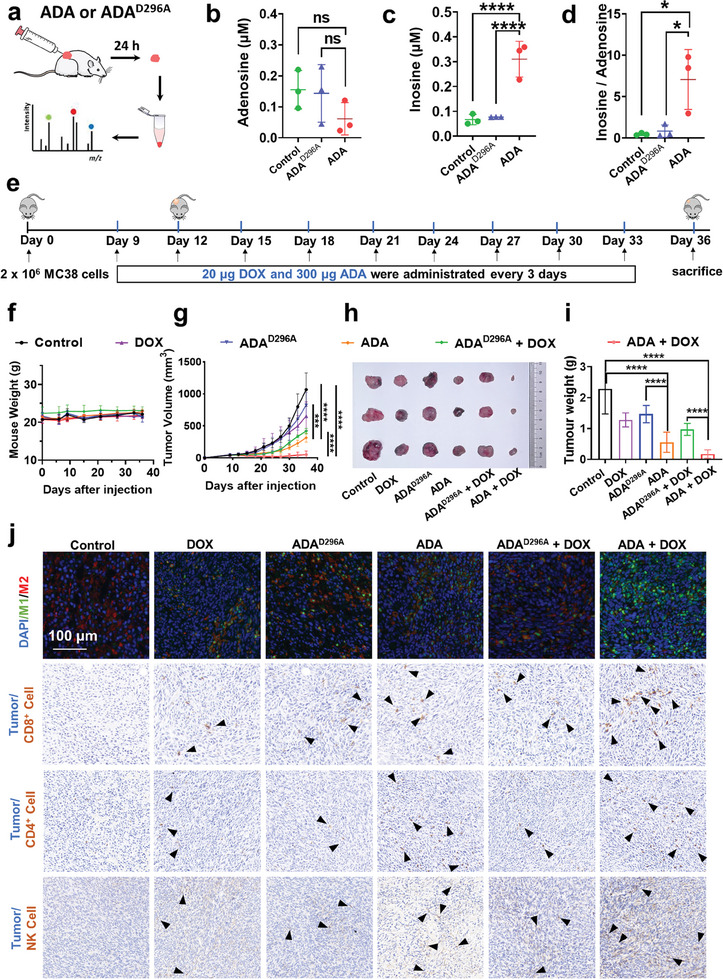
Adenosine deaminase in combination with doxorubicin exhibits synergistic anti‐tumor effect in mouse subcutaneous tumor model. A) Schematic illustration of ADA activity evaluation in a mouse MC38 subcutaneous tumor model. Intratumoral concentrations of adenosine b) and inosine c), along with the inosine‐to‐adenosine ratio d), were measured in MC38 tumor‐bearing mice after different treatments and plotted (n = 3). e) Schematic illustration of the combinational therapy of adenosine deaminase and doxorubicin in a mouse MC38 subcutaneous tumor model. Body weight f) and tumor volume changes g) of MC38 tumor‐bearing mice after different treatments were plotted (n = 6). Representative tumor figures h) and tumor weights i) of MC38 tumors in mice from different treatment groups (n = 3). Error bars indicate standard deviation of three mice in each experimental group. Differences between groups were assessed using one‐way ANOVAs, followed by Bonferroni's correction for multiple comparisons. Significance levels were indicated as follows: *p < 0.05, **p < 0.01, ***p < 0.001, ****p < 0.0001. j) Immunohistochemical staining of tumor sections reveals infiltration of M1 (green) and M2 (green) macrophages, CD8^+^ T cells, CD4^+^ T cells, and NK cells. The black triangles indicate the infiltrated immune cells. Scale bar: 50 µm.

Previous studies demonstrated that D296 is a key residue for the catalytic function of ADA.^[^ ^40^ ^]^ Therefore, an ADA^D296A^ variant was constructed as a negative control. Deficiency in the catalytic activity of ADA^D296A^ toward adenosne was confirmed by HPLC analysis. (Figure S6, Supporting Information). Initially, in vivo experiment using C57BL/6 subcutaneous tumor models (MC38) were conducted to examine the catalytic activity of ADA in the TME. After intratumoral injection of 50 µg ADA or ADA^D296A^, the inosine concentration in mice tumor significantly increased in the ADA‐treated group within 24 h, while no significant change was observed in the ADA^D296A^‐treated group (**Figure** **2**a–d). The in vivo experiment confirmed the catalytic function of ADA in the TME.

Doxorubicin (DOX), a clinically used chemotherapy drug for CRC and an inducer of immunogenic cell death, was combined with ADA to enhance the efficacy of chemo‐immunotherapy. The potential synergistic effect of ADA and DOX was initially validated at the cellular level. Equal numbers of polarized M1 macrophages and MC38 tumor cells were co‐cultured with 100 µM adenosine under three conditions: DOX alone, ADA alone, and ADA + DOX, for 4 h. Fluorescence microscopy revealed that, in the presence of 100 nM ADA and 50 µM DOX, tumor cell phagocytosis by M1 macrophages increased significantly to ≈80%, surpassing the levels observed with either DOX or ADA alone (Figure S7, Supporting Information). These results provide strong evidence for the synergistic effect of ADA and DOX.

We next sought to evaluate the clinical efficacy of the recombinant ADA when paired with the chemotherapy agent DOX in cancer therapy using a subcutaneous tumor mouse model. To determine the optimal dosages of ADA and DOX, C57BL/6 mice bearing MC38 tumors in the flank region were treated with PBS, DOX, ADA, and ADA + DOX every three days. At 70 µg DOX, tumor suppression was effective in both DOX and DOX + ADA groups, with no significant difference observed, indicating that DOX alone overshadowed the combination's synergy. To reduce DOX's cardiotoxicity, its dose was lowered to 20 µg in subsequent experiments to enhance synergy while minimizing toxicity (Figure S8, Supporting Information). The 100 µg ADA group showed no antitumor effect, likely due to poor tumor targeting and loss during peritumoral injection. To address this, the ADA dose was increased to 300 µg in subsequent studies.

In the formal experiments, C57BL/6 mice bearing MC38 tumors in the flank region was treated with PBS, DOX, ADA^D296A^, ADA, ADA^D296A^ + DOX and ADA + DOX every three days. Notably, ADA^D296A^ and ADA (300 µg) were administered via peritumoral injection, while low‐dose DOX (20 µg) was delivered intravenously via mice tail vein. On the 36th day post tumor implantation, the mice were sacrificed for tumor collection (Figure 2e). No significant changes in mice body weight were observed during the treatments, indicative that the treatments had no substantial toxicity in the mice (Figure 2f). Administration of either DOX or ADA^D296A^ alone did not exhibit significant effects on tumor growth compared to the PBS‐treated mice. By contrast, ADA and ADA^D296A^ + DOX administration substantially attenuated tumor growth. In particular, the combination of ADA and DOX even resulted in complete suppression of tumor growth, suggesting synergistic anti‐tumor effects between ADA and DOX (Figure 2g–i). The collected tumor tissues were further subjected to immunofluorescence staining. A notable increase in M1 macrophage abundance were observed across all the treated group. In particular, the ADA + DOX group exhibited the highest M1 macrophage density alongside the lowest M2 macrophage count, indicative of the M2‐to‐M1 switch within the TME and enhancement of anti‐tumor immune response. Furthermore, notable augmentation of CD4^+^, CD8^+^ T cells and natural killer (NK) cells infiltration within the TME was evident in both the ADA and ADA + DOX groups, with the ADA + DOX combination therapy demonstrating the most pronounced enhancement of immune cell infiltration (Figure 2j; Figure S9a–d, Supporting Information). These data collectively suggested that peritumoral ADA administration facilitates immune cell infiltration into the TME, while ADA + DOX combination therapy provides highly potent synergistic anti‐tumor effects.

### 2.3 Construction and Characterization of Engineered Probiotic *E. coli* for Adenosine‐conversion

**Figure 3:**
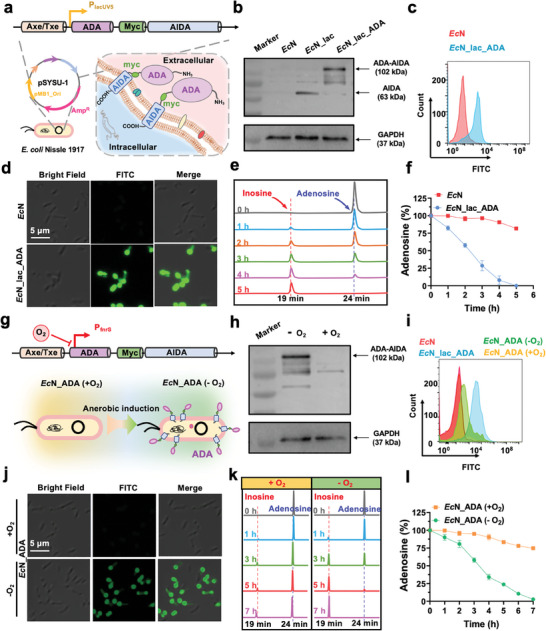
Construction and characterization of engineered probiotic. a) Schematic illustration of *E. coli* Nissle 1917 (*Ec*N) surface‐display system of adenosine deaminase (ADA) with a lacUV5 promoter (P_lacUV5_). b) Western blot analysis to verify the expression of ADA‐AIDA fusion protein. Flow cytometry c) and immunofluorescence imaging d) analysis of the surface‐displayed ADA on engineered *E. coli* Nissle 1917 (*Ec*N_lac_ADA). e) HPLC profiles of adenosine after 5 h incubation with *Ec*N_lac_ADA. The elution times for adenosine and inosine are indicated. f) Quantification of adenosine conversion by wild‐type *Ec*N and *Ec*N_lac_ADA within 5 h. g) Schematic illustration of *Ec*N surface‐display system of ADA with a hypoxia‐controlled fnrS promoter (P_fnrS_). h) Western blot analysis to verify the expression of ADA‐AIDA fusion protein under anaerobic condition. Flow cytometry i) and immunofluorescence imaging j) analysis of hypoxia‐induced ADA surface display on engineered *E. coli* Nissle 1917 (*Ec*N_ADA). k) HPLC profiles of adenosine after 5 h incubation with *Ec*N_ADA under aerobic and anaerobic conditions. The elution times for adenosine and inosine are indicated. l) Quantification of adenosine conversion by *Ec*N_ADA under anaerobic or aerobic condition within 7 h.

To achieve the desired anti‐tumor effect, relatively high doses of ADA must be targeted administrated to the solid tumor site. However, peritumoral injection of ADA is impracticable for clinic use. This highlights the need to develop alternative approaches to locally and continuously deliver ADA to tumor. Previous studies showed that a probiotic *E. coli* Nissle 1917 (*Ec*N) could selectively colonize and thrive in hypoxic tumor site.^[^ ^20^ ^]^ Consequently, we sought to engineer the probiotic as a synthetic biology‐based cellular therapy for intratumoral ADA production. We envisioned that ADA display on the bacterial surface would optimize the catalysis of extracellular adenosine. Therefore, we initially evaluate the feasibility of displaying ADA on *Ec*N surface utilizing a bacterial surface display system that we had previously reported.^[^ ^31^ ^]^ In brief, the human ADA1 (40 kDa) was fused to the N‐terminus of a bacterial AIDA‐I autotransporter domain.^[^ ^25^ ^]^ The gene encoding the fusion protein was under control of a lacUV5 promoter (P_lacUV5_) for constitutive expression (**Figure** **3**a). The constructed plasmid was subsequently transformed into *Ec*N to prepare the *Ec*N_lac_ADA strain. Immunoblotting and flow cytometry analysis revealed that the ADA‐AIDA‐I fusion protein was successfully expressed on the *Ec*N surface and oriented toward the cell exterior (Figure 3b,c), which was further verified by the fluorescence bacterial staining analysis (Figure 3d). Bacterial growth curves revealed that *Ec*N*_*lac_ADA exhibits similar a growth rate compared to that of *Ec*N, indicating that the genetic modification did not adversely affect the bacterial proliferation (Figure S10, Supporting Information). We next sought to assess the enzymatic activity of bacterial surface‐displayed ADA. In brief, equal amount of *Ec*N or *Ec*N_lac_ADA (OD_600_ = 0.25) were incubated with 1 mM adenosine in 10 mM HEPES buffer at 37 °C. The adenosine and inosine contents in the buffer were measured by HPLC every hour. Notably, adenosine was completely converted to inosine by *Ec*N_lac_ADA within 5 h, whereas only 20% of adenosine was catalyzed by wild‐type *Ec*N (Figure 3e,f; Figure S11, Supporting Information). These data unequivocally underscore the remarkable adenosine conversion proficiency of the surface‐displayed ADA.

When utilized for tumor treatment, constitutive expression of ADA by *Ec*N_lac_ADA could potentially have adverse effect on normal tissues or provoke immune responses against the bacteria. In addition, sustained expression would also impose a metabolic burden on the bacteria, potentially compromising their viability and persistence during the treatment.^[^ ^41^ ^]^ The constitutive lacUV5 promoter was subsequently replaced with a hypoxia‐inducible fnrS promoter (P_fnrS_)^[^ ^42^ ^]^ to construct the *Ec*N_ADA strain. This modification should enable the engineered *Ec*N_ADA strain to selectively express ADA within the hypoxic regions of tumors, thereby enhancing treatment specificity and reducing adverse effects (Figure 3g). Tumor tissues exhibit lower oxygen levels (0.1%–2%, −200 to −300 mV) than normal tissues (5%–10%, −150 to −250 mV) due to rapid cell proliferation and poor blood supply.^[^ ^43^ ^]^ Therefore, we used 1% oxygen to mimic hypoxic conditions in tumors. Immunoblotting results showed that the ADA‐AIDA‐I fusion protein was only expressed in hypoxia conditions rather than normoxia (Figure 3h). Similarly, flow cytometry analysis revealed that the bacterial surface‐displayed ADA in the *Ec*N_ADA strain was only detectable under hypoxic conditions, although the level was relatively lower compared to that of the *Ec*N_lac_ADA strain (Figure 3i). Moreover, bacterial immunofluorescence staining further confirmed the successful surface display of ADA under hypoxic conditions rather than normoxic conditions (Figure 3j). In consistent, the *Ec*N_ADA strain could completely catalyzed 1 mM adenosine into inosine within 7 h under hypoxic condition, whereas only ≈25% adenosine could be converted under normoxic condition (Figure 3k,l). Furthermore, the impact of the promoter switch on enzymatic activity was also evaluated by comparing the catalytic efficiency of *Ec*N_ADA and *Ec*N_lac_ADA in converting 1 mM adenosine under 1% oxygen condition. HPLC analysis showed that *Ec*N_lac_ADA achieved almost 100% conversion at 300 min, compared to ≈70% for *Ec*N_ADA, indicative of slightly reduced catalytic activity (Figure S12, Supporting Information). Overall, these findings demonstrate that the engineered *Ec*N_ADA strain expresses and presents ADA on its surface in response to hypoxia, resulting in effective catalysis of adenosine into inosine in vitro.

### 2.4 Engineered Probiotic *E. coli* Synergizes with Chemotherapy to Facilitate the Regression of Mouse Subcutaneous Tumor

To assess the compatibility of *Ec*N_ADA with DOX in therapy, we first investigated the impact of DOX on the engineered probiotics. Intriguingly, experimental results showed that 400 µM DOX had no significant effect on bacterial ADA expression and catalytic activity (Figure S13, Supporting Information). Additionally, the catalytic activity of bacterial surface‐displayed ADA and purified ADA enzyme was compared in the presence of DOX. Enzyme‐linked immunosorbent assay (ELISA) results showed that ≈0.2 nmol of ADA enzyme was diplayed on 10^8^ CFU of *Ec*N_ADA. Based on this, the adenosine conversion rates of bacterial surface‐displayed ADA and purified ADA were calculated as 167 and 70 min^−1^, respectively (Figure S14, Supporting Information).

**Figure 4:**
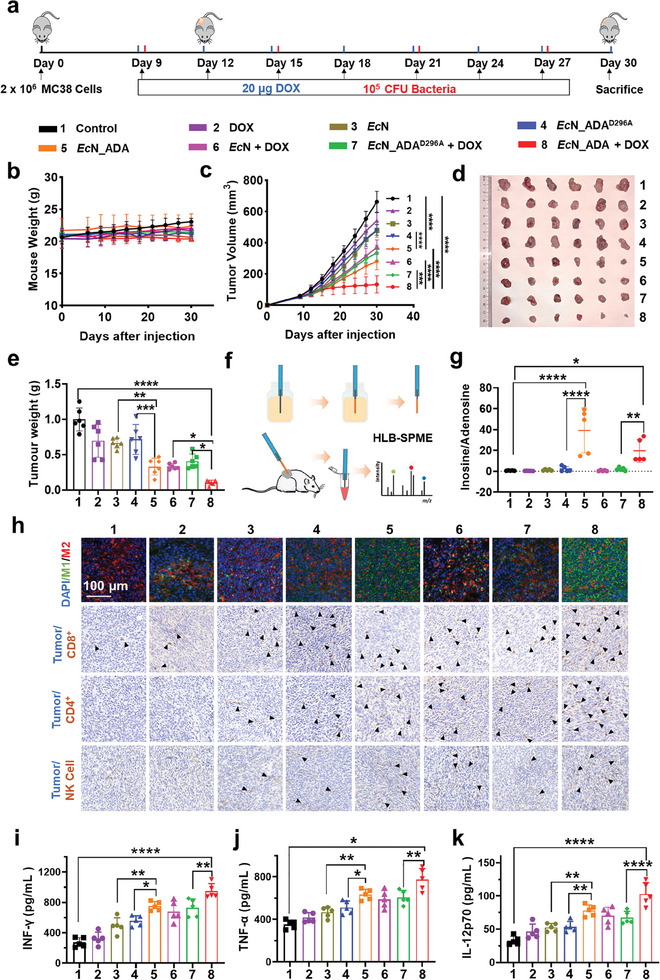
Combining engineered probiotics and doxorubicin inhibit solid tumor growth in mouse subcutaneous tumor model. a) Schematic illustration of the combinational therapy of engineered probiotics and doxorubicin in a mouse MC38 subcutaneous tumor model. Body weight b) and tumor volume changes c) of MC38 tumor‐bearing mice after different treatments were plotted (n = 6). Representative of tumor figures d) and tumor weights e) of MC38 tumors in mice from different treatment groups (n = 6). Error bars indicate standard deviation of three mice in each experimental group (n = 6). f) Schematic illustration of the experimental procedure to quantify adenosine and inosine in murine tumor tissues using the solid‐phase microextraction (SPME). g) Quantified inosine/adenosine ratios in murine tumor tissues in different experimental groups. h) Immunohistochemical staining of tumor sections reveals infiltration of M1 (green) and M2 (green) macrophages, CD8+ T cells, CD4+ T cells, and NK cells. The black triangles indicate the infiltrated immune cells. Scale bar: 50 µm. i–k) Secreted cytokine levels of INF‐γ i), TNF‐α j), IL12‐p70 k) in murine serum in different experimental groups. Error bars indicate standard deviation of three mice in each experimental group (n = 6).Differences between groups were assessed using one‐way ANOVAs, followed by Bonferroni's correction for multiple comparisons. Significance levels were indicated as follows: *p < 0.05, **p < 0.01, ***p < 0.001, ****p < 0.0001.

We subsequently investigated whether the *Ec*N_ADA could synergize with low‐dose DOX chemotherapy for cancer treatment using a subcutaneous tumor mouse model. In addition, a mutant *Ec*N_ADA^D296A^ strain without catalytic activity was also constructed as a negative control, and deficiency in the catalytic activity of *Ec*N_ADA^D296A^ strain toward adenosne was confirmed (Figure S15, Supporting Information). To determine the optimal dosage of engineered probiotics for treatment, a dose of 10⁶ CFU of bacteria was administered intravenously to mice, but this resulted in significant mortality within 10 days. While reducing the bacterial dose to 10⁵ CFU eliminated mortality throughout the treatment period (Figure S16, Supporting Information). Notably, a single intravenous administration of 10⁵ CFU engineered bacteria persisted in mouse tumors for over 6 days (Figure S17, Supporting Information). Therefore, a bacterial dose of 10⁵ CFU was established as the optimal dosage for both therapeutic efficacy and safety in subsequent animal studies. In brief, C57BL/6 mice bearing MC38 tumors the in the flank region were treated with mono‐ or combinational therapy, including PBS, DOX, *Ec*N, *Ec*N_ADA^D296A^, *Ec*N_ADA, *Ec*N + DOX, *Ec*N_ADA^D296A^ + DOX and *Ec*N_ADA + DOX. Typically, 20 µg of DOX in a total volume of 100 µL was administered via intravenous injection for each mouse every three days. Tail vein injections of bacteria were conducted at a concentration of 1 × 10^6^ CFU mL^−1^ in PBS, with a total volume of 100 µL injected per mouse every five days. On the 30th day post tumor implantation, the mice were sacrificed for tumor collection (**Figure** **4**a). Throughout the treatment period, no significant decreases in mice body weight were observed in any experimental groups, implying that both mono‐ and combination therapy exhibited no significant toxicity on the mice (Figure 4b). As shown in Figure 4c, administration of DOX, *Ec*N and *Ec*N_ADA^D296A^ alone exhibited no significant effects on tumor growth compared to the control group. In contrast, administration of *Ec*N_ADA resulted in a substantial slowdown in tumor growth, with the final tumor weight being only ≈50% of that in the control group. Intriguingly, the combination therapy of *Ec*N + DOX or *Ec*N_ADA^D296A^ + DOX also slowed down the tumor growth rate, despite the lack of significant impact on tumor growth observed with *Ec*N, *Ec*N_ADA^D296A^ or DOX alone, implying a synergistic effect between the probiotic‐based therapy and chemotherapy. Remarkably, the administration of *Ec* N_ADA + DOX led to a pronounced suppression in tumor growth rate and resulted in the lightest tumor weights (Figure 4d,e). After mice sacrifice, various tissues were collected, homogenized and analyzed for retained bacteria. The results showed that the engineered bacteria primarily accumulated at the tumor site, with minimal presence in the heart, liver, spleen, lungs, and kidneys, indicative of the targeted colonization ability of the engineered bacteria in tumors. Notably, both the engineered *Ec*N_ADA^D296A^ and *Ec*N_ADA colonized and persisted in tumors to a similar extent as *Ec*N, indicating that genetic manipulation did not alter the tumor colonization features of *Ec*N (Figure S18, Supporting Information).

In vivo hypoxic‐induced expression of ADA by the engineered probiotics was confirmed by myc‐FITC antibody staining of the tumor sections. Green fluorescence from the myc‐tag linked to ADA or ADA^D296^ was observed in tumor treated with *Ec*N_ADA, *Ec*N_ADA + DOX, *Ec*N_ADA^D296A^ or *Ec*N_ADA^D296A^ + DOX, but not in tumor treated with *Ec*N, confirming in situ expression of ADA or ADA^D296A^ by the engineered probiotics (Figure S19, Supporting Information). Moreover, immunohistochemistry (IHC) analysis of the mice heart, liver, spleen, lungs, and kidneys showed no significant damage in the *Ec*N + DOX group compared to others, further confirming the safety of the combinational therapy (Figure S20, Supporting Information).

To investigate the metabolic changes in the TME after various treatments, we employed hydrophilic‐lipophilic balanced solid‐phase microextraction (HLB‐SPME) method to measure the levels of adenosine and inosine in solid tumors (Figure 4f). The calculated concentration of adenosine in TME was 0.04 µM in the control group, which is in consistent with the values reported previously.^[^ ^44^ ^]^ Unexpectedly, no significant change on the adenosine levels were observed among all the treated groups (Figure S21, Supporting Information). In contrast, substantially increased levels of inosine were detected in *Ec*N_ADA and *Ec*N_ADA + DOX‐treated group, indicative of successful intratumoral enzymatic conversion of adenosine to inosine (Figure 4g; Figure S22, Supporting Information). One possible explanation for this discrepancy could be the rapid turnover of adenosine within the TME, wherein newly generated adenosine replenishes the converted adenosine, resulting in a relatively stable concentration.

Immunofluorescence analysis of tumor sections showed that most tumor‐associated macrophages in the control group exhibited an M2‐like phenotype, consistent with an immunosuppressive condition. Administration of DOX, *Ec*N, *Ec*N_ADA^D296A^, *Ec*N + DOX or *Ec*N_ADA^D296A^ + DOX led to a slight increase the number of M1‐like macrophage within tumors. In contrast, treatment with *Ec*N_ADA and *Ec*N_ADA + DOX substantially elevated the M1/M2 macrophage ratio in the TME, suggesting a shift toward a more immunostimulatory TME (Figure 4h; Figure S23, Supporting Information). Moreover, immunohistochemical analysis demonstrated a significant increase in infiltration of CD4^+^ cells, CD8^+^ cells, and NK cells in tumors from mice treated with *Ec*N_ADA and *Ec*N_ADA + DOX compared to other experimental groups. Particularly, the co‐administration of *Ec*N_ADA and DOX resulted in the highest infiltration of these immune cell populations, highlighting the synergistic immune‐stimulatory effects of *Ec*N_ADA and DOX combination therapy (Figure S24a–d, Supporting Information). In consistent, ELISA analysis of the mouse serum demonstrated that the EcN_ADA + DOX treatment resulted in the highest levels of INF‐γ, TNF‐α, and IL‐12p70 cytokines compared to other treated groups (Figure 4i–k). These data further confirmed the activation of immune cells such as T cells and macrophages, which are known to produce these cytokines upon activation.

### 2.5 Single‐Cell RNA (scRNA) Sequencing Profiling of Mouse Subucaneous Tumor

**Figure 5:**
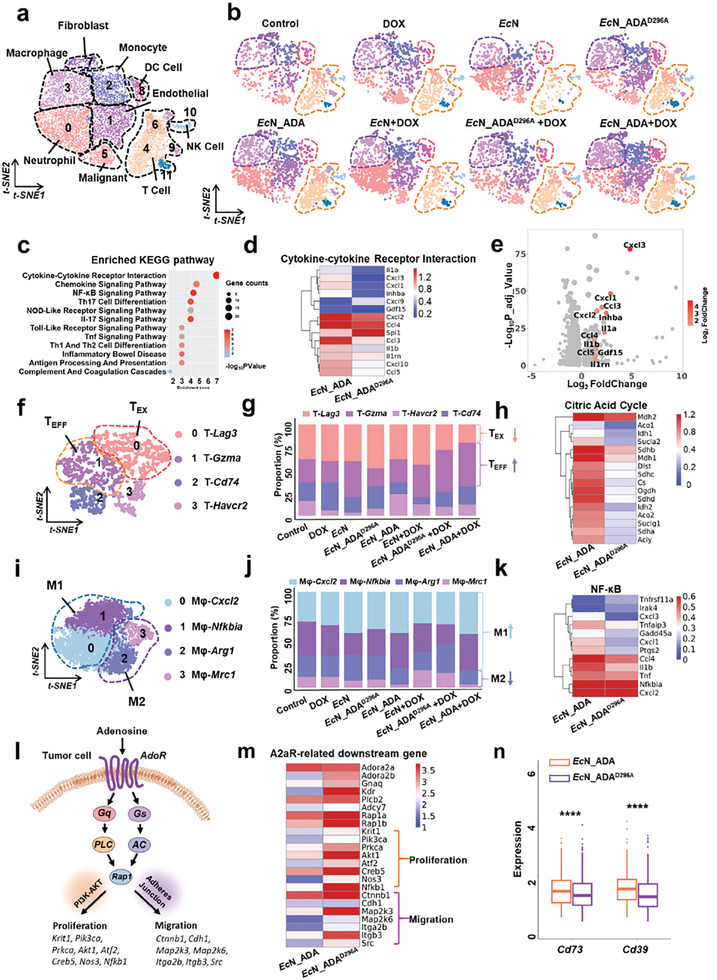
Single‐cell RNA (scRNA) sequencing analysis of the mice tumor tissues after treatment. a) t‐distributed stochastic neighbor embedding (t‐SNE) representation of all the identified cell populations in mice tumors. Labels have been added on the basis of the expression of marker genes. NK, natural killer cell. DC, dendritic cell. b) t‐SNE plots are shown separated by different treatment conditions. Macrophage are highlighted in purple, DC cells are highlighted in red and T cells are highlighted in orange. c) KEGG pathway enrichment analysis of differentially expressed genes (DEGs) of all the immune cells between *Ec*N_ADA and *Ec*N_ADA^D296A^‐treated group. Heatmap d) and volcano plot e) showing the differentially expressed genes in cytokine‐cytokine receptor interaction pathway of immune cells between *EcN*_ADA and *Ec*N_ADA^D296A^‐treated groups. f) t‐SNE plot of all identified T cell populations, with labels based on marker gene expression. The exhausted T cell cluster is highlighted in red, and the effector T cell cluster is highlighted in yellow. g) The proportions of the four populations of T cells in mice tumor tissues after different treatments. h) Heatmap depicting the differentially expressed genes in citric acid cycle pathway of T cells between *Ec*N_ADA and *Ec*N_ADA^D296A^‐treated group. i) t‐SNE plot of all identified macrophage populations, with labels based on marker gene expression. The M1 macrophage cluster is highlighted in blue, and the M2 macrophage cluster is highlighted in purple. j) The proportions of the four populations of macrophage in mice tumor tissues after different treatments. k) Heatmap depicting the differentially expressed genes in NF‐κB pathway of macrophage between *Ec*N_ADA and *Ec*N_ADA^D296A^‐treated group. l) Schematic illustration of downstream genes involved in tumor proliferation and migration in the adenosine receptor (A2aR) signaling pathway. m) Heatmap depicting the differentially expressed genes in A2aR signaling pathway of tumor cells between *Ec*N_ADA and *Ec*N_ADA^D296A^‐treated group. n) The average transcriptional levels of *Cd73* and *Cd39* genes in tumor cells from *Ec*N_ADA and *Ec*N_ADA^D296A^‐treated group. Statistical analyses were performed using the Gehan‐Wilcoxon test followed by Bonferroni's correction for multiple comparisons for all survival analyses. ****p < 0.0001.

To further investigate how *Ec*N_ADA enhances anti‐tumor immune responses in mouse subcutaneous tumor, the collected tumors were subjected to single‐cell RNA sequencing (scRNA) analysis. After quality control and batch correction, all the identified cells were classified into 5 different cell populations with a reference‐based type annotation method followed by manual curation of top marker genes. These populations include tumor cells, fibroblast, macrophage, myeloid cells, and T cells (Figure S25, Supporting Information). Globally, the combinational treatment of *Ec*N_ADA and *Ec*N_ADA + DOX induced substantial changes in the population of both myeloid cells and lymphocytes, leading to a notably higher abundance of T cells and myeloid cells (Figure S26, Supporting Information). Sub‐setting and re‐clustering of all immune cells further resulted in 12 clusters, which were classified into six immune cell subtypes after annotation, including T cells, NK cells, DC cells, monocytes, macrophages, and neutrophils (**Figure** **5**a; Figure S27, Supporting Information). Similarly, the results also revealed significant increase in the population of macrophages, DC cells and T cells after *Ec*N_ADA or *Ec*N_ADA + DOX treatment (Figure 5b). KEGG enrichment analysis on the differentially expressed genes (DEGs) of immune cells between *Ec*N_ADA and *Ec*N_ADA^D296A^‐treated groups revealed increased activation of cytokine‐cytokine interaction and inflammation regulatory pathways, such as NF‐κB, NOD‐like receptor and Toll‐like receptor signaling pathways in *Ec*N_ADA‐treated tumors, indicative of induced inflammation responses in *Ec*N_ADA‐treated tumors (Figure 5c). In particular, the upregulation of chemokine and cytokine genes such as *Cxcl1, Cxcl2, Cxcl3, Ccl3, Ccl4, Ccl5*, *Il1a, Il1b* and *Il1rn* indicates a significant enhancement in immune cell recruitment within *Ec*N_ADA‐treated tumors (Figure 5d,e). The unexpected downregulation of *Cxcl9* may be due to the reduced expression of *Spi1*, a key transcription factor that regulates *Cxcl9* expression.^[^ ^45^ ^]^ Overall, the results are in consistent with the enhancement in immune cells infiltration in tumor section analysis.

We subsequently sought to investigate the effects of *Ec*N_ADA treatment on T cells and macrophages individually. The sub‐setting of total T cells yielded four clusters, with a typical exhausted T cell cluster (T_EX_, cluster 0) and an effector T cell cluster (T_EFF_, cluster 1) (Figure 5f; Figure S28, Supporting Information). Notably, differential gene expression analysis showed a pronounced exhausted T cells reduction and a substantially increase in the antitumor effector T cells within tumors from *Ec*N_ADA + DOX‐treated mice relative to other experimental groups (Figure 5g). KEGG enrichment analysis of total T cells between *Ec*N_ADA and *Ec*N_ADA^D296A^‐treated tumors also revealed increases in pathways related to anti‐tumor immune responses, such as cytokine‐cytokine receptor interaction and natural killer cell mediated cytotoxicity (Figure S29a–c,Supporting Information). In particular, the tricarboxylic acid (TCA) cycle‐related genes exhibited significant upregulation in the *Ec*N_ADA‐treated compared to the *Ec*N_ADA^D296A^‐treated group (Figure 5h), indicative of T cell metabolic reprogramming and activation.^[^ ^46^, ^47^ ^]^ Indeed, flow cytometry analysis showed a significant increase in CD3+ T cell infiltration in tumors treated with *Ec*N_ADA + DOX (Figure S30, Supporting Information), indicating that the therapy promotes T cell infiltration into the TME.

The probiotic‐based therapy also affected intratumoral macrophage populations (Figure 5i; Figure S31, Supporting Information). Differential gene expression analysis unveiled that tumor from mice treated with *Ec*N_ADA or *Ec*N_ADA + DOX contained significantly fewer immunosuppressive M2‐like macrophages characterized by high expression levels of hallmark genes such as *Arg1* or *Mrc1* (clusters 2 and 3). Instead, there was a notable shift toward M1‐like macrophages predominantly expressing antitumoral effector molecules, such as *Il1b*, *Tnf* and *Cxcl2* (cluster 0 and 1; Figure 5j). In consistent, KEGG enrichment analysis further corroborated the M2 to M1 macrophage phenotypic switch in tumors treated with *Ec*N_ADA, evidenced by the elevation in pathways associated with M1 macrophage polarization, including the typical NF‐κB signaling pathway^[^ ^48^ ^]^ (Figure 5k; Figure S32a–c, Supporting Information). Flow cytometry analysis showed a significant increase in the M1/M2 macrophage ratio in tumors treated with *Ec*N_ADA + DOX (Figure S33, Supporting Information), supporting the notion that the combination therapy promotes macrophage reprogramming from the M2 to M1 phenotype within the TME.

In addition to immune cell inhibition, adenosine could also bind to the A2aR receptor in tumor cells and activate the downstream PI3K‐AKT pathway, thereby promoting tumor cell proliferation and migration^[^ ^49^, ^50^ ^]^ (Figure 5l). Given the efficient adenosine conversion by *Ec*N_ADA treatment, we also examined the impact of *Ec*N_ADA treatment on the A2aR signaling pathway in tumor cells. After sub‐setting, the transcriptional levels of genes associated with the A2aR signaling pathway in all tumor cells were assessed. Overall, *Ec*N_ADA treatment significantly downregulated genes related to the A2aR signaling pathway in tumor cells, including *Gnaq, Plcb2, Pap1b* in comparison with the *Ec*N_ADA^D296A^‐treated group (Figure 5m). Importantly, three key genes essential for tumor proliferation and metastasis, namely *Ccnd1*, *Vegfa*, and *Hif1a*, were also downregulated after *Ec*N_ADA treatment (Figure S34, Supporting Information). Collectively, all these results suggested that the *Ec*N_ADA treatment has the potential to inhibit tumor cell proliferation and metastasis by reducing the activity of the A2aR signaling pathway. Intriguingly, we observed a significant upregulation in the transcriptional levels of the two ecto‐nucleotidases Cd73 and Cd39, responsible for generating extracellular adenosine in the *Ec*N_ADA‐treated group compared to the *Ec*N_ADA^D296A^‐treated group (Figure 5n). This upregulation may be attributed to a compensatory feedback effect following the removal of extracellular adenosine.

### 2.6 Engineered Probiotic Together with Low‐Dose Chemotherapeutics Inhibits Tumor Growth in a Mouse Orthotopic CRC Model

**Figure 6:**
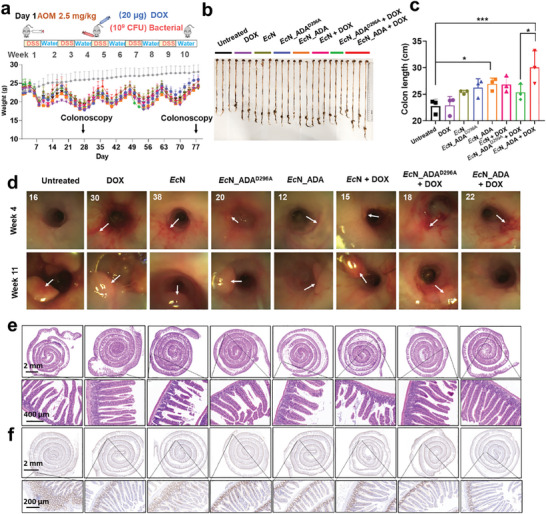
Combining engineered probiotics with low‐dose doxorubicin inhibit tumor growth in mouse orthotopic colorectal cancer model. a) Schematic illustration of the mouse orthotopic colorectal cancer model establishment and combinational therapy procedure. Mice body weights are recorded every three days. The days for the colonoscopy analysis are indicated. b) Representative colon figures of mice after different treatments. c) Statistical analysis of mice colon lengths from different treatment groups (n = 3). Error bars indicate standard deviation of three mice in each experimental group. Two‐tailed Student's t‐test was used to determine statistical significance. *p < 0.05, **p < 0.01, ***p < 0.001, ****p < 0.0001. d) Colonoscopy imaging shows the condition of colorectal carcinoma in mice at week 4 (baseline) and week 11 (post‐treatment) across different treatment groups. Tumors are marked with white arrows, and ear tag IDs are used to distinguish mice within each group: Control (16, 27, 33), DOX (6, 30, 39), *Ec*N (38, 36, 51), *Ec*N_ADA^D296A^ (20, 34, 37), *Ec*N_ADA (12, 8, 25), *Ec*N + DOX (19, 2, 15), *Ec*N_ADA^D296A^ + DOX (18, 7, 28), and *Ec*N_ADA + DOX (22, 46, 1). e) Hematoxylin and eosin (H&E) staining of rolled small intestine sections showing villous architecture integrity and crypt depth in mice from different treatment groups. f) Ki67 staining of rolled small intestine sections showing the oncogenic state of mice from different treatment groups.

The oral administration of probiotics presents a preferable and effective alternative to intravenous delivery, providing sustained benefits in mitigating systemic toxicities. Oral administration of 10^9^ CFU engineered *Ec*N_ADA persisted in mouse intestine for over 6 days, suggesting that the engineered probiotics hold the potential to provide long‐term therapeutic effects within the gut (Figure S35, Supporting Information). Inspired by the anti‐tumor efficacy of the engineered probiotic, we proceeded to evaluate its therapeutic potential using an orthotopic CRC mouse model. In brief, 3‐week‐old C57BL/6c mice underwent a 10‐week continuous induction period involving the initial administration of the carcinogenic agent azoxymethane (AOM) and the colitis‐inducing compound dextran sodium sulfate salt (DSS). To mitigate the risk of severe colitis and mortality associated with prolonged DSS exposure, mice were alternatively given a diet consisting of 2.5% DSS and water on a weekly basis.^[^ ^51^ ^]^ After confirming successful model establishment via colonoscopy in the fourth week, the mice were randomly assigned to eight groups. During the following six weeks, each group received various treatments, including PBS, DOX, *Ec*N, *Ec*N_ADA^D296A^, *Ec*N_ADA, *Ec*N + DOX, *Ec*N_ADA^D296A^ + DOX, and *Ec*N_ADA + DOX. Among these treatments, 20 µg of DOX in a total volume of 100 µL was administered via intravenous injection through the tail vein every three days. Meanwhile, 100 µL bacterial suspension in PBS with a concentration of 1 × 10^10^ CFU mL^−1^ was administrated via oral gavage every five days. At week 11, another colonoscopy was conducted to evaluate the progression of intestinal inflammation and colorectal tumor of mice in each experimental group and followed by euthanasia (**Figure** **6**a). Given the close correlation between the severity of colonic lesions and colon length, the colons of the mice were subsequently collected and measured. Notably, mice in the untreated group exhibited significant colonic lesions, characterized by a considerable reduction in colon length to ≈22.8 ± 1.3 cm. In contrast, mice receiving the *Ec*N_ADA + DOX treatment had the longest colon lengths among all the experimental groups with an average length of 30.1 ± 3.1 cm, indicative of substantial alleviation of colonic lesions post‐treatment (Figure 6b,c). Colonoscopy analysis showed that majority of mice treated with AOM/DSS exhibited colon inflammation or small‐sized tumors by week 4, indicating the successful establishment of the orthotopic CRC model. By week 11, colonoscopy examination revealed the presence of more and large tumors as well as severe colonic inflammation in all experimental groups except the *Ec*N_ADA + DOX‐treated group, implying that the combinational therapy could relieve intestinal inflammation and inhibit the growth of orthotopic colorectal tumor (Figure 6d; Figures S36 and S37 and Movie S1, Supporting Information).

In consistent, histopathologic analysis of the mouse colon sections revealed significant epithelial barrier damage and crypt abscesses in untreated mice, those receiving monotherapy, and those treated with *Ec*N_ADA^D296A^ + DOX. Notably, a substantial portion of the observed intestinal mucosal epithelial layer was shed, indicative of severe colonic inflammation or the presence of colonic tumors. In contrast, colon tissues from mice treated with *Ec*N_ADA + DOX largely maintained the integrity of the epithelial barrier (Figure 6e). Immunohistochemically analysis of small intestine sections showed that mice treated with *Ec*N_ADA + DOX had significantly longer villi and decreased expression of Ki‐67, a proliferation biomarker for tumor, compared to other experimental groups, indicative of an improvement in intestinal lesions (Figure 6f; Figure S38, Supporting Information). Furthermore, staining with FITC anti‐Myc antibodies confirmed the presence of bioengineered bacteria surrounding the villi, indicating successful colonization of the small intestine villous walls by the orally administered bacteria (Figure S39, Supporting Information). Notably, high levels of INF‐γ, TNF‐α, and IL‐12p70 cytokines were detected in all treated groups, which were likely caused by severe colitis induced by prolonged DSS administration (Figure S40, Supporting Information).

The impact of *Ec*N_ADA on intestinal microbiota was evaluated in light of its therapeutic potential. Species abundance analysis of the intestinal microbiota across the PBS, *Ec*N_ADA^D296A^, and *Ec*N_ADA treatment groups revealed notable differences (Figure S41a, Supporting Information). The *Ec*N_ADA treatment significantly increased *Parabacteroides* abundance and showed trends of elevated *Bacteroides acidifaciens*, *Bacteroides sartorii*, and *Lactobacillus johnsonii*, all of which are associated with intestinal health (Figure S42b, Supporting Information). Meanwhile, no significant changes in Chao1 and Shannon indices were observed, indicating that oral administration of engineered bacteria did not significantly affect overall microbial diversity or richness (Figure S42c,d, Supporting Information). Collectively, all these results demonstrated that the combination therapy of *Ec*N_ADA and low‐dose DOX could efficiently inhibit tumor growth in an orthotopic CRC mouse model.

## 3 Conclusion

Immunotherapy has revolutionized cancer treatment, leveraging the patient's immune system to combat tumors. Despite its success, the immunosuppressive TME, largely mediated by adenosine metabolites, often hinders its efficacy. Targeting the adenosinergic pathway to mitigate this suppression holds great promise. In this study, we constructed a probiotic strain of *E. coli* Nissle 1917 (*Ec*N) to express human ADA on its surface under hypoxic conditions typical of TME. This approach capitalizes on the unique capabilities of bacteria, particularly their ability to selectively colonize hypoxic tumor environments, self‐propel, and trigger anti‐tumor immune responses due to their intrinsic immunostimulatory nature. The engineered strain of *Ec*N (*Ec*N_ADA) not only enhances the local concentration of ADA, converting immunosuppressive adenosine into inosine and thus alleviating immune suppression, but also synergizes effectively with chemotherapeutic agents such as DOX. Our preclinical studies have demonstrated that *Ec*N_ADA, particularly when used in combination with low‐dose DOX, markedly improves immune cell infiltration into tumors, reprograms macrophages from an anti‐inflammatory (M2) to a pro‐inflammatory (M1) phenotype, and substantially suppresses tumor growth in both subcutaneous and orthotopic mouse tumor models. While this study primarily validated the therapeutic efficacy of *Ec*N_ADA using the MC38 CRC model. Nonetheless, the strategy of utilizing probiotics to convert adenosine into inosine within the TME holds promise for treating a wide range of solid tumors, particularly those with high CD73 and CD39 expression. These findings suggest that the synthetic biology approach of using engineered probiotics can effectively remodel the TME, enhancing the efficacy of existing cancer therapies and potentially leading to better clinical outcomes. Moreover, the adaptability of bacteria for genetic and chemical modifications opens up numerous possibilities for further enhancements. Future research should focus on optimizing the delivery and persistence of these engineered bacteria within the TME. Ensuring stable and controlled expression of therapeutic enzymes in response to specific tumor conditions remains a critical challenge. Additionally, combining this bacterial therapy with other immunomodulatory treatments, such as checkpoint inhibitors, could further enhance antitumor responses and improve patient outcomes.

In summary, the development of engineered *Ec*N_ADA represents a novel approach in cancer treatment, leveraging the natural properties of bacteria and the principles of synthetic biology to overcome the challenges of tumor‐induced immunosuppression. We believed that this strategy holds significant potential to enhance the effectiveness of cancer immunotherapies.

## 4 Experimental Section

### Protein Expression and Purification

Codon optimized full‐length ADA genes from *Homo sapiens*, *Bifidobacterium pseudolongum* and *Lactobacillus johnsonii* were synthesized by Genewiz and sub‐cloned into pET‐47b vector using restriction sites of BamHI and SacI, yielding pET47b‐ADA, pET47b‐*Bp*ADA and pET47b‐*Lj*ADA. Plasmid of inactive mutant ADA^D296A^ was generated via site‐directed mutagenesis using wild‐type pET47b‐ADA plasmid as DNA template. Primers for DNA amplification and site‐directed mutagenesis were listed in Table S1 (Supporting Information). The constructed plasmids were transformed into BL21(DE3) cells for expression after sequencing. For ADA, *Bp*ADA and *Lj*ADA protein expression, transformed bacteria were cultured in Luria‐Bertani (LB) medium containing 30 µg mL^−1^ kanamycin. 5 mL overnight culture was transferred into 1 L fresh LB medium supplemented with respective antibiotics at 37 °C with agitation until OD_600_ reaches 0.6. Isopropyl β‐D‐1‐thiogalactopyranoside (IPTG) was added to a final concentration of 0.5 mM to induce protein expression. Bacteria were further incubated at 25 °C for 16 h and collected by centrifugation at 4000 g for 10 min. Ni‐NTA buffer A (40 mM Tris‐HCl, pH 8.0, 500 mM NaCl, 10 mM imidazole, 1 mM phenylmethanesulfonyl fluoride (PMSF) and 1 mM Tris(2‐carboxyethyl) phosphine (TCEP) was used to resuspend the bacteria cell pellet. The cell pellet was lysed by the high‐pressure homogenizer. The bacterial lysate was centrifuged at 20 000 g for 30 min, then the supernatant was filtered by a 0.22 µm filter and loaded onto the Ni‐NTA column pre‐equilibrated by Ni‐NTA buffer A. Bound proteins were eluted by Ni‐NTA buffer B (40 mM Tris‐HCl, pH 8.0, 500 mM NaCl, 500 mM imidazole, 1 mM PMSF and 1 mM TCEP). The eluted proteins were dialyzed against dialysis buffer (40 mM Tris‐HCl, pH 8.0, 250 mM NaCl and 1 mM TCEP) and Precision protease was added to cleave the His6‐tag at 4 °C overnight. The dialyzed sample was then purified using a Superdex increase 200 10/300 column (Cytiva) pre‐equilibrated with GF buffer (20 mM Tris‐HCl, pH 7.5, 300 mM NaCl and 1 mM TCEP).

### In Vitro Enzyme Activity Assay

In vitro enzyme activity of ADA was determined by measuring the enzymatic generated NH_4_^+^ using the indophenol blue method as described previously.^[^ ^52^ ^]^ In brief, 10 mL of reagent A (1 mM adenosine in 20 mM HEPES buffer, pH 6.8) was mixed with 10 µL of purified enzyme (100 µM ADA) and incubated at 37 °C. An aliquot of 50 µL of reaction sample was collected every 5 min. Subsequently, 50 µL of reagent B (106 mM phenol and 0.17 mM sodium nitroprusside) and 50 µL of reagent C (11 mM NaOCl and 125 mM NaOH) were added into the 50 µL reaction sample and vortexed immediately. The mixture was then incubated for an additional 30 min at 37 °C. Finally, reaction mixture was transferred to a 96‐well plate, and the absorbance was measured at 628 nm using a plate reader (BioTek, USA).

### Activation of Mouse CD8^+^ T Cells

Mouse CD8^+^ T cells were enriched from spleen of 8‐week‐old female mouse (C57BL/6) by negative selection using an EasySep mouse Naïve CD8^+^ T cell isolation kit (STEMCELL Technologies) according to the manufacturer's instructions. For CD8^+^ T cells activation analysis, freshly isolated naïve mouse CD8^+^ T cells were maintained in RPMI 1640 medium (Gibco) supplemented with 10% FBS (Gibco) and mouse IL‐2 (100 U mL^−1^) (Pepro Tech). The cells were activated using plate‐bound anti‐mouse CD3 (clone 145‐2C11) (BioLegend) and anti‐mouse CD28 (clone 37.51) (BioLegend) antibodies under the indicated experimental conditions for 24 h. For the analysis of surface marker CD25, live cells were stained with PerCP/cyanine5.5 anti‐mouse CD25 (eBioscience); After fixation and permeabilization, the treated CD8^+^ T cells were stained with PerCP/Cyanine5.5 anti‐mouse IFN‐γ antibodies (BioLegend), followed by flow cytometry analysis. For the analysis of sustained effector T cell function under glucose‐depleted conditions, freshly isolated naive mouse CD8^+^ T cells in RPMI 1640 medium supplemented with 10% FBS and 5 ng mL^−1^ mouse IL‐2 (100 U mL^−1^) and IL‐12 (5 ng mL^−1^) were pre‐activated by plate‐bound anti‐mouse CD3 and anti‐mouse CD28 antibodies for 24 h. Then CD8^+^ T cells were then transferred into Glucose‐free RPMI 1640 medium (10% FBS) under the indicated experimental conditions and cultured for an additional four days. After fixation and permeabilization, the T cells were stained by PC5.5 anti‐mouse INF‐γ, FITC anti‐mouse TNF‐α and APC anti‐ mouse Granzyme B antibodies (BioLegend), followed by flow cytometry analysis.

### Mouse Macrophage Polarization Analysis

Mouse bone marrow cells from thigh bone of 8‐week‐old female mouse (C57BL/6) were harvested and cultured as previously described.^[^ ^53^ ^]^ In brief, the mouse thigh bone was isolated and cut open. The bone marrow cells were washed out with PBS buffer supplemented with 10% FBS. Red blood cells were then removed by incubating the bone marrow cells in red blood cell lysis buffer (Beyotime). The retained bone marrow cells were cultured in sodium pyruvate free‐DMEM (Gibco) medium supplemented with 10% FBS and 100 ng mL^−1^ GM‐CSF (Sino biological) for another 6 days to differentiate into mature bone‐marrow derived‐macrophages (BMDMs). The BMDMs were either activated with 100 ng mL^−1^ IFN‐γ (Sino biological) and 100 ng mL^−1^ LPS (Sigma–Aldrich) (M1‐like macrophages), or activated by 100 ng mL^−1^ IL‐4 (PeproTech) (M2‐like macrophages) for another 2 days. For macrophage polarization analysis, macrophage from indicated experimental conditions were stained with PE/Cyanine7 anti‐mouse F4/80, PE anti‐mouse CD86, and APC anti‐mouse CD206 antibodies (BioLegend), followed by flow cytometry analysis to distinguish between M1 and M2 subsets.

### Assessment of ADA Enzymatic Activity in Mouse Subcutaneous Tumor

C57BL/6 mice aged six to eight weeks were subcutaneously inoculated with 10^6^ MC38 cells. After the tumor volume reached ≈100 mm^3^, the mice were administered a single injection of 100 nM ADA, ADA^D296A^, or PBS. Twenty‐four hours post‐injection, the mice were euthanized, and tumors were excised and digested with collagenase IV (Sigma–Aldrich) into single‐cell suspensions. Following centrifugation, the supernatant was collected for HPLC analysis of adenosine and inosine concentrations. 100 nM lamivudine was added as an internal reference. Quantification was conducted using an Agilent 1290 Infinity II UHPLC system coupled to an Agilent 6475 Triple Quad tandem mass spectrometer in multiple reaction monitoring mode (MRM), operating in positive mode. Chromatographic separation was achieved on a ZORBAX RRHD Eclipse Plus C18 column (150 mm × 3 mm, 1.8 µm), with a 5 µL injection volume. Gradient elution utilized water with 0.1% FA as mobile phase A and acetonitrile as mobile phase B. The flow rate was maintained at 300 µL min^−1^. Additional instrument parameters included a nebulizer pressure of 28 psi, gas flow of 8.5 L min^−1^, sheath gas flow of 11.5 L min^−1^, capillary voltage of 3200 V, nozzle voltage of 400 V, and temperatures set to 300 °C for the gas and 280 °C for the sheath gas. The parent ion/product ion pairs of adenosine (268.1/135.9), inosine (269.1/136.8) and lamivudine (230.1/94.8) were used for quantification, and the parent ion/product ion pairs 268.1/118.9, 269.1/109.9 and 230.1/44.9 were used for qualification.

### Macrophage Phagocytosis Assay

Approximately 10^5^ MC38 cells were seeded on a glass dish and stained with 10 ng mL^−1^ Fluorescein Diacetate (Sigma–Aldrich) for 30 min. After washing with PBS to remove excess dye, 10^5^ pre‐activated M1‐type RAW264.7 macrophages were co‐cultured with the MC38 cells under various drug treatments as indicated. After 2 h, APC anti‐mouse F4/80 antibody (BioLegend) was added to label the macrophages, and the phagocytosis was visualized using confocal imaging.

### Bacterial Strain Construction

The plasmid for *E. coli* surface‐display system was constructed as described previously.^[^ ^31^ ^]^ In brief, genes encoding the plasmid stabilization system Axe/Txe cassette, human ADA 1 with a lacUV5 promoter (P_lacUV5_), and the bacterial surface‐display system AIDA were synthesized by Genewiz. A pUC19 plasmid containing an ampicillin resistant gene (amp^R^) and a high copy number origin of replication ColE1 was chosen as plasmid backbone. The three gene fragments and the pUC19 plasmid backbone were subsequently seamlessly assembled by Gibson assembly method to construct the plasmid pSYSU‐lac. For hypoxia‐induced protein expression, the lacUV5 promoter was replaced with the fnrS promoter by restriction enzyme digestion and ligation method, resulting the plasmid pSYSU‐fnrs. These plasmids were used to electroporate *E. coli* Nissle 1917. After electroporation, the transformed bacterial cells were selected as colonies on LB agar containing carbenicillin antibiotic. The plasmid maps, DNA sequences, and corresponding primers were available in Figure S42, Tables S2 and S3 (Supporting Information).

### Western‐Blot Analysis

To investigate the ADA expression level in engineered bacteria, cultured bacteria in log phrase (OD_600_ = 0.6) was collected. Dilute each group to the same OD_600_ value, then add SDS‐PAGE loading buffer and heat in a 95 °C metal bath for 30 min for bacterial lysis. Bacterial total protein sample were separated by SDS‐PAGE and transferred to PVDF membranes. After blocking with 5% (wt/vol) non‐fat dry milk solution, the membranes were individually incubated with primary antibody, including anti‐myc (1:1000) (Proteintech) and anti‐GAPDH (1:2000) (Bioworld) polyclonal rabbit antibodies. After overnight incubation at 4 °C, the membranes were washed with TBST buffer and then incubated with goat anti‐rabbit HRP‐conjugated second antibody (1:1000) (Proteintech) for 1 h at 25 °C.

### Flow Cytometry Analysis

For the analysis of surface markers of immune cells, the cells were stained in PBS containing 3% (wt/vol) BSA and the appropriate antibodies. For intracellular cytokine staining of immune cells, the cells were fixed with 4% paraformaldehyde at room temperature for 30 min, followed by permeabilization with 0.1% Triton X‐100 at room temperature for 10 min. The cells were then stained with the indicated antibodies. For the detection of bacterial ADA surface‐display, 500 µL of bacterial culture in log‐phrase (OD_600_ = 0.7) were blocked in PBS buffer with 3% BSA for 20 min at 37 °C. After centrifugation at 4000 rpm for 10 min, the bacterial pellets were resuspended in 100 µL PBS buffer containing FITC anti‐Myc‐ antibody (10 µg mL^−1^) (Abcam) and incubated at room temperature for 30 min on a tube rotator. Subsequently, the bacterial pellets were collected by centrifugation and washed in 500 µL ice‐cold PBS buffer for three times, and followed by flow cytometer analysis. All the data were acquired on a CytoFLEX S flow cytometry (Beckman Coulter) and were analyzed with FlowJo 10.8.1 software.

### HPLC Quantification of Purine Nucleosides

For analysis of adenosine‐to‐inosine conversion, the engineered bacteria with an OD_600_ value of 0.25 were incubated with 1 mM adenosine in 10 mM HEPES buffer (pH 7.4) in a 37 °C shaker. An aliquot of 10 µL of the solution was collected every 1 h. After centrifugation and filtration, the concentrations of adenosine and inosine in the supernatant were quantitatively analyzed by HPLC (Thermo Fisher Ultimate 3000) equipped with a C18‐H column (4.6 × 250 mm, 5 µm, 120 Å) (Thermo Fisher) at a flow rate of 0.7 mL min^−1^ at 25 °C. The mobile phase consisted of a linear gradient between 0.1% TFA in water (Buffer A) and 0.1% TFA in acetonitrile (Buffer B). The HPLC gradient program maintained 99% Buffer B for the first 5 min, then linearly decreased from 99% to 90% over the next 20 min (5–25 min). The peaks for inosine and adenosine appeared at 19 and 24 min, respectively. The chromatogram was recorded via UV absorption at 254 nm.

### Immunofluorescence

All the fluorescence images of the cells and bacteria were taken on a Carl Zeiss confocal microscope. To examine the effect of adenosine and inosine on M1 and M2 macrophage, polarized M1‐like or M2‐like macrophages were cultured in 35 mm confocal culture dishes for 24 h. Subsequently, the cells were incubated in the indicated experimental conditions for another 24 h and washed with PBS. After cell fixation with 4% paraformaldehyde and blocking, the cells were then stained by FITC anti‐mouse CD206 and APC anti‐mouse CD80 antibody (Biolegend) at 4 °C for 12 h. The cell nuclei were stained with Hoechst 33 342 (Beyond). To investigate the ADA expression level on bacterial surface, 500 µL of bacterial culture in log‐phrase (OD_600_ = 0.7) were blocked in PBS buffer with 3% BSA for 20 min at 37 °C. After centrifugation at 4000 rpm for 10 min, the bacterial pellets were incubated with 100 µL PBS buffer containing FITC anti‐Myc antibody (10 µg mL^−1^) at room temperature for 30 min on a tube rotator. Subsequently, the bacterial pellets were collected by centrifugation and washed in 500 µL ice‐cold PBS buffer for three times, and followed by fluorescence images collection.

### Subcutaneous Tumor Models and In Vivo Studies

The animal experiment procedures were approved by the Institutional Animal Care and Use Committee of Sun Yat‐sen University (approval number: 2024001413) and performed in accordance with the ethical guidelines of Sun Yat‐sen University. All the experiments were conducted in the Laboratory Animal Center of Sun Yat‐sen University. 8‐week‐old SPF‐grade female C57BL/6J mice were purchased from GemPharmatech Co., Ltd., Nanjing, Jiangsu, China. MC38 tumor cells (2 × 10^6^ cells in 100 µL PBS) were subcutaneously injected into the left mouse flank. Nine days after tumor implantation, mice with similar tumor sizes were randomly divided into different experimental groups. To evaluate the clinical efficacy of the purified ADA enzyme combined with the chemotherapy agent DOX in cancer therapy, the mice were randomly assigned to 6 groups of 6 mice each: Control, DOX, ADA^D296A^, ADA, ADA^D296A^ + DOX, and ADA + DOX. Approximately 300 µg of ADAD^296A^ or ADA were administered via peritumoral injection every three day, while low‐dose DOX (20 µg) was delivered intravenously via the tail vein every three days. To investigate the synergistic anti‐cancer effect of *Ec*N_ADA combined with low‐dose DOX chemotherapy, mice were randomly grouped into 8 groups of 6 mice each: Control, DOX, *Ec*N, *Ec*N_ADA^D296A^, *Ec*N_ADA, *Ec*N + DOX, *Ec*N_ADA^D296A^ + DOX, and *Ec*N_ADA + DOX. 20 µg of DOX in a total volume of 100 µL was administered via intravenous injection for each mouse every three days. Tail vein injections of bacteria were conducted at a concentration of 1 × 10^6^ CFU mL^−1^ in PBS, with a total volume of 100 µL injected per mouse every five days. The length (L), width (W), and height (H) of the tumors and the body weight of mice were measured and recorded every 3 day. The tumor volume (V) was calculated using the formula V (mm^3^) = L (mm) × W (mm) × H (mm) × 0.5. The tumor‐bearing mice were euthanized at the indicated day after tumor implantation, and the tumors were stripped out for photograph and weight. For immunohistochemistry (IHC) analysis, the tumors were fixed with 4% paraformaldehyde at 4 °C and embedded in paraffin, and then the CD3^+^ cells, CD8^+^ cells, NK cells and macrophage (M1 and M2) were visualized by immunohistochemically staining. For quantitative assessment of resident bacteria in different organs, the heart, liver, spleen, lungs, and kidneys of the mice were harvested, weighed, and ground in sterile saline. The homogenates were then subjected to plate spreading (ampicillin‐resistant) to enumerate the remaining bacteria. For cytokine quantification, mice serum was collected, and the levels of INF‐γ, TNF‐α, and IL‐12p70 were measured using commercial ELISA kits according to the manufacturer's instructions.

### Quantification of Adenosine and Inosine in Murine Tumor Tissues

Adenosine and inosine were quantified using the solid‐phase microextraction (SPME) technique by referring to the literature.^[^ ^54^ ^]^ Home‐made HLB (hydrophile‐lipophile balance) SPME fibers were used to extract adenosine and inosine from tumor tissues. Briefly, medical‐grade stainless steel wires (SSWs, 400 µm diameters) were sonicated in concentrated hydrochloric acid (1 mol/L) to ≈10 min. The HLB (hydrophile‐lipophile balance) particles (20% of the total volume) were combined with polyacrylonitrile (PAN, average molecular weight 250 000, 15% w/w in DMF solvent) under continuous stirring for 24 h. Utilizing a dip‐coating technique. Kinetic Calibration^[^ ^55^ ^]^ was employed to quantify free adenosine and inosine in the vicinity of tumor cells within mice models. Lamivudine (Thermo Scientific, USA) was served as the internal standard owing to its structural similarity to adenosine and inosine. Mice were anesthetized with isoflurane, and the HLB‐PAN fiber was then introduced into the tumor, penetrating ≈1 cm deep. After the designated 30 min sampling duration, the fiber was carefully retracted under vortex at 550 rpm for 30 min. Quantification was conducted using an Agilent 1290 Infinity II UHPLC system coupled to an Agilent 6475 Triple Quad tandem mass spectrometer in multiple reaction monitoring mode (MRM), operating in positive mode. Chromatographic separation was achieved on a ZORBAX RRHD Eclipse Plus C18 column (150 mm × 3 mm, 1.8 µm), with a 5 µL injection volume. Gradient elution utilized water with 0.1% FA as mobile phase A and acetonitrile as mobile phase B. The flow rate was maintained at 300 µL min^−1^. Additional instrument parameters included a nebulizer pressure of 28 psi, gas flow of 8.5 L min^−1^, sheath gas flow of 11.5 L min^−1^, capillary voltage of 3200 V, nozzle voltage of 400 V, and temperatures set to 300 °C for the gas and 280 °C for the sheath gas. The parent ion/product ion pairs of adenosine (268.1/135.9), inosine (269.1/136.8) and lamivudine (230.1/94.8) were used for quantification, and the parent ion/product ion pairs 268.1/118.9, 269.1/109.9 and 230.1/44.9 were used for qualification.

### In Vivo Bioluminescence Imaging of Engineered Probiotics

To monitor the retention time of engineered probiotics in subcutaneous tumors or intestines of mice, the LuxCDABE reading frame fragment was inserted into the pSYSU‐fnrs plasmid and electroporated into *Ec*N to generate *Ec*N_Lux_ADA. A total of 10^5^ CFU of *Ec*N_Lux_ADA was intravenously injected into tumor‐bearing (MC38) mice, and bacterial accumulation was tracked daily. After isoflurane anesthesia, luminous signals were measured using a chemiluminescent imager (Fx6, Vilber, France) to determine bacterial numbers and distribution. For intestinal retention analysis, 10 mice were gavaged with 10^9^ CFU of *Ec*N_Lux_ADA. At predetermined intervals (12 h and 24 h, 1 day, 2 days, 3 days, 4 days, and 6 days post‐gavage), one mouse was euthanized per time point. The intestines, along with the heart, liver, spleen, lungs, and kidneys, were harvested to evaluate bacterial distribution.

### Single‐Cell RNA Sequencing

To prepare the single‐cell RNA sequencing samples, tumor tissues were rinsed three times with Hank's balanced salt solution (HBSS) and then minced into fragments measuring 1–2 mm in diameter. The tissue fragments were digested using 2 mL of GEXSCOPE tissue dissociation solution (Singleron) at 37 °C for 15 min with continuous agitation. Following digestion, the resulting cell suspension was filtered through a 40‐micron sterile filter and centrifuged at 1000 rpm for 5 min. The supernatant was discarded, and the cells were resuspended in 1 mL of PBS (HyClone). The suspension was then centrifuged at 500 g for 5 min, and the pellet was resuspended in 1 mL of PBS. Cell viability and counts were determined using Trypan blue staining (Sigma) under a microscope. Single‐cell suspensions were prepared in PBS (HyClone) at a concentration of 1 × 10^5^ cells/mL. The suspensions were loaded onto microfluidic chips, and single‐cell RNA sequencing libraries were constructed using the GEXSCOPE single‐cell RNA library kit (Singleron Biotechnologies) in accordance with the manufacturer's instructions. Libraries were diluted to 4 nM and sequenced using an Illumina NovaSeq6000 platform with 150 bp paired‐end reads. Raw sequencing data were processed using Singleron's internal analysis pipeline to generate a gene expression matrix. Briefly, reads lacking poly T sequences were removed, and valid cell barcodes and unique molecular identifiers (UMIs) were extracted. Reads were filtered to remove adapters and poly A tails (using fastp V1), and aligned to the reference genome from the Ensemble database for quantification (using STAR 2.5.3a and featureCounts 1.6.2). Reads, UMIs, and genes with the same cell barcode were grouped, and the UMI counts per gene in each cell were calculated for downstream analysis. The Seurat package^[^ ^56^ ^]^ was employed for cell type identification and clustering analysis of the RNA sequencing data. Expression matrices were imported into R using the read. Table function, and cell clusters were determined using the FindClusters function with a resolution parameter of 0.6. Differentially expressed genes (DEGs) between different samples or across clusters were identified using the FindMarkers function. GO enrichment analysis of gene sets was conducted using the clusterProfiler package to identify biological functions or pathways significantly associated with specific gene expressions.

### Orthotopic Colorectal Cancer Model and Colonoscopy Analysis

The animal experiment procedures were approved by the Institutional Animal Care and Use Committee of Sun Yat‐sen University (approval number: 2024001414) and performed in accordance with the ethical guidelines of Sun Yat‐sen University. All the experiments were conducted in the Laboratory Animal Center of Sun Yat‐sen University. C57BL/6 mice (4–6 weeks) were treated with 7.5 mg kg^−1^ azoxymethane (AOM) (Sigma) by intraperitoneal injection at the beginning of the experiment. After 7 days, the mice were given 5 cycles of 2.5% DSS in drinking water for 7 days, followed by regular water for 14 days. Mice intestinal endoscopy equipment (ENDOQ) was used to detect the colon cancer lesions in mice on day 29 and day 78 to assess the therapeutic effects of different treatments. At 78 day, the mice were sacrificed, and blood and tissues were collected for subsequent analysis and experiments. The length of colon was measured to assess the severity of CRC. Tissue samples of colon cross‐sections and Swiss rolls were preserved using paraformaldehyde, subsequently encased in paraffin blocks, sliced into thin sections, and then subjected to H&E staining and ki‐67 immunofluorescent staining. For H&E staining, histological pathology evaluation for acute colitis and CRC was performed based on the severity, extent of damage, inflammation, and regeneration of the colon. For immunofluorescent staining, DAPI and FITC anti‐Myc antibody was used to visualize the distribution of engineered bacterial in the colon.

## Conflict of Interest

The authors declare no conflict of interest.

## Author Contributions

W.X., Z.‐W.M. and J.‐H.W. conceived and designed the study. J.‐H.W., J.W., Z.Y. and J.‐Q.W. conducted the animal model experiments. C.Z., Y.L. and J.‐H.W. conducted the T Cells experiments. H.W. and J.X. participated in solid‐phase microextraction experiment and data analysis. J‐H.W., J.Z. and C.X. participated in scRNA‐seq analysis. W.X. and J‐H.W. wrote the manuscript.

## Supporting information



Supporting Information

Supplemental Movie 1

## Data Availability

The data that support the findings of this study are available from the corresponding author upon reasonable request.
