# Supplementary material for: Targeting the Adenosine‐Mediated Metabolic Immune Checkpoint with Engineered Probiotic for Enhanced Chemo‐Immunotherapy
Source: Adv Sci (Weinh). 2025 Feb 22;12(15):2411813. doi: 10.1002/advs.202411813 (PMC12005768; doi:10.1002/advs.202411813)
Supplement: Supplementary file 1 — Supporting Information [file ADVS-12-2411813-s002.pdf]

ADVANCED SCIENCE
Open Access

Supporting Information

for *Adv. Sci.*, DOI 10.1002/advs.202411813

Targeting the Adenosine-Mediated Metabolic Immune Checkpoint with Engineered Probiotic for Enhanced Chemo-Immunotherapy

*Jinhui Wang, Jing Wang, Zhijie Yu, Hongyu Wen, Chensi Zhao, Jiayong Zhong, Chuanle Xiao, Yingqiu Li, Jianqiao Xu, Jinquan Wang, Zong-Wan Mao and Wei Xia**

Supplementary Information

Targeting the Adenosine-mediated Metabolic Immune Checkpoint with Engineered Probiotic for Enhanced Chemo-immunotherapy

Jinhui Wang[1], Jing Wang[1], Zhijie Yu[2], Hongyu Wen[1], Chensi Zhao[4], Jiayong Zhong[3], Chuanle Xiao[3], Yingqiu Li[4], Jianqiao Xu[1], Jinquan Wang[2], Zong-Wan Mao[1] and Wei Xia[1,*]

[1] MOE Key Laboratory of Bioinorganic and Synthetic Chemistry, IGCME, School of Chemistry, Sun Yat-Sen University, Guangzhou 510006, China.

[2] Guangdong Key Laboratory of Advanced Drug Delivery, School of Bioscience and Biopharmaceutics, Guangdong Pharmaceutical University, Guangzhou 510006, China.

[3] State Key Laboratory of Ophthalmology, Zhongshan Ophthalmic Center, Guangdong Provincial Key Laboratory of Ophthalmology and Visual Science, Sun Yat-sen University, Guangzhou 510060, China.

[4] MOE Key Laboratory of Gene Function and Regulation, State Key Laboratory of Biocontrol, School of Life Sciences, Sun Yat-sen University, Guangzhou, 510006, China

Email: xiawei5@mail.sysu.edu.cn

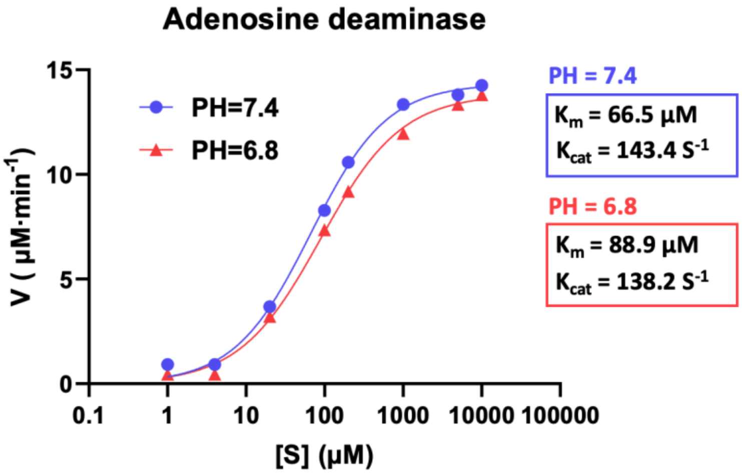

Supplementary Fig. 1 The kinetic activity of adenosine deaminase was assessed under various pH conditions.

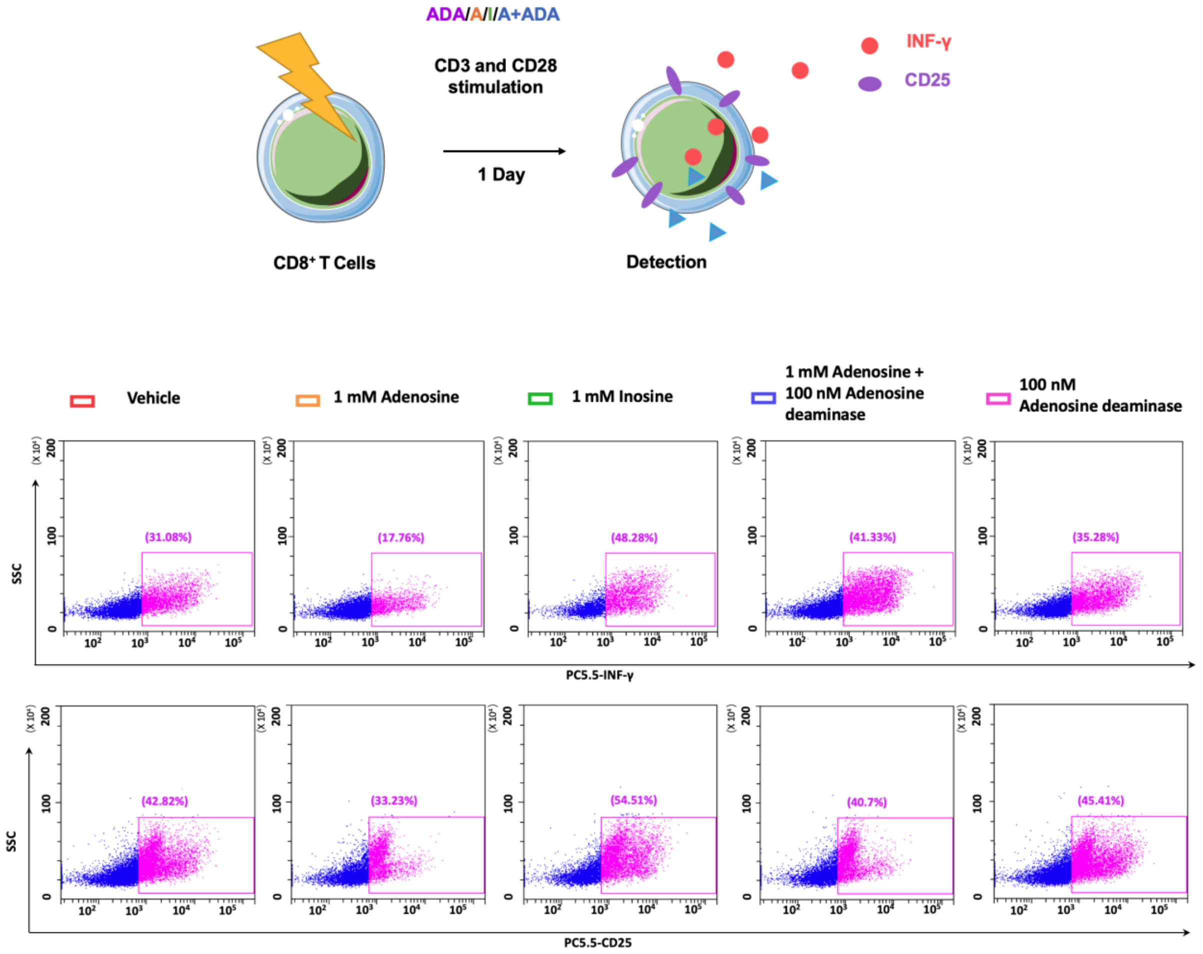

Supplementary Fig. 2. The proportions of $CD8^+$ $INF\text{-}\gamma^+$ T cells and $CD8^+$ $CD25^+$ T cells within the $CD8^+$ T cell population were measured by flow cytometry in different treatment groups. All experiments were performed in triplicate (n=3). Representative flow cytometry plots are shown.

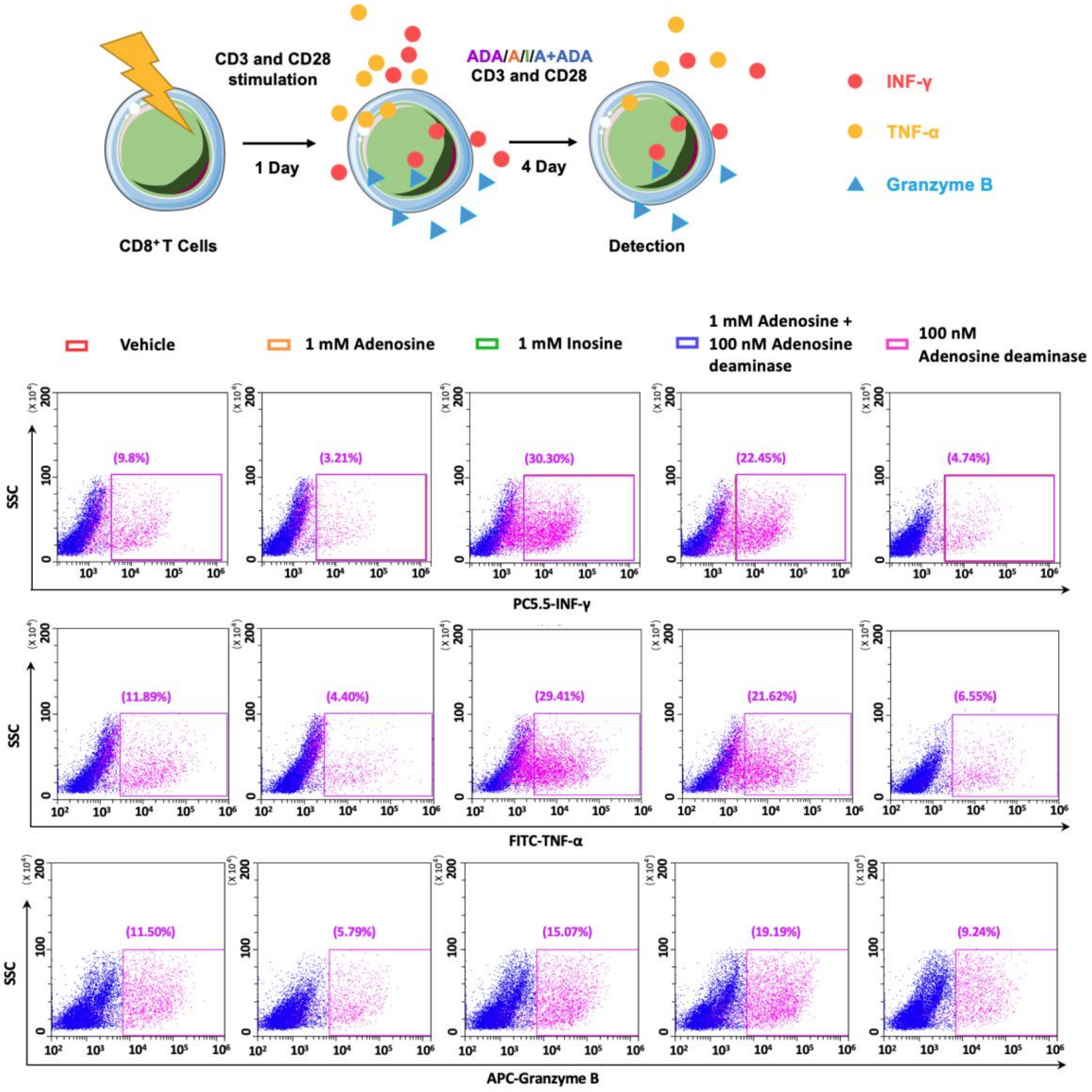

Supplementary Fig. 3 The proportion of $CD8^+$ INF-γ^+ T cells, $CD8^+$ TNF-α^+ T cells and $CD8^+$ $GZMB^+$ T cells within the $CD8^+$ T cell population were measured by flow cytometry in different treatment groups. All experiments were performed in triplicate (n=3). Representative flow cytometry plots are shown.

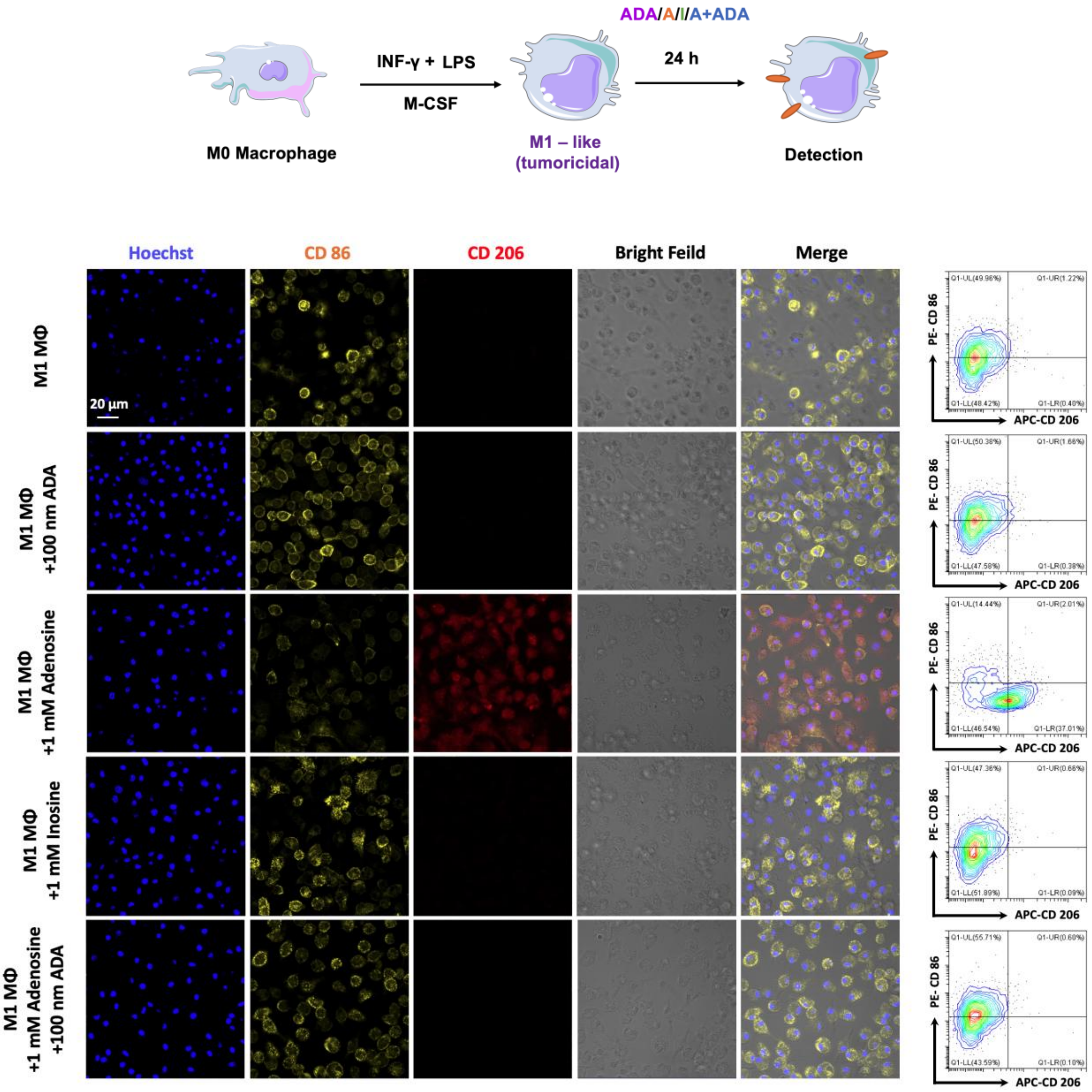

Supplementary Fig. 4 Immunofluorescence staining was performed to identify M1 macrophages in different treatment groups. Cell nuclei were stained with Hoechst (blue), M1 macrophages were stained with PE-CD86 (yellow), and M2 macrophages were stained with APC-CD206 (red). Flow cytometry analysis was conducted to determine the proportions of M1 and M2 macrophages in different treatment groups by co-staining with PE-CD86 and APC-CD206. All experiments were performed in triplicate (n=3) and the data are expressed as mean ± s.d.

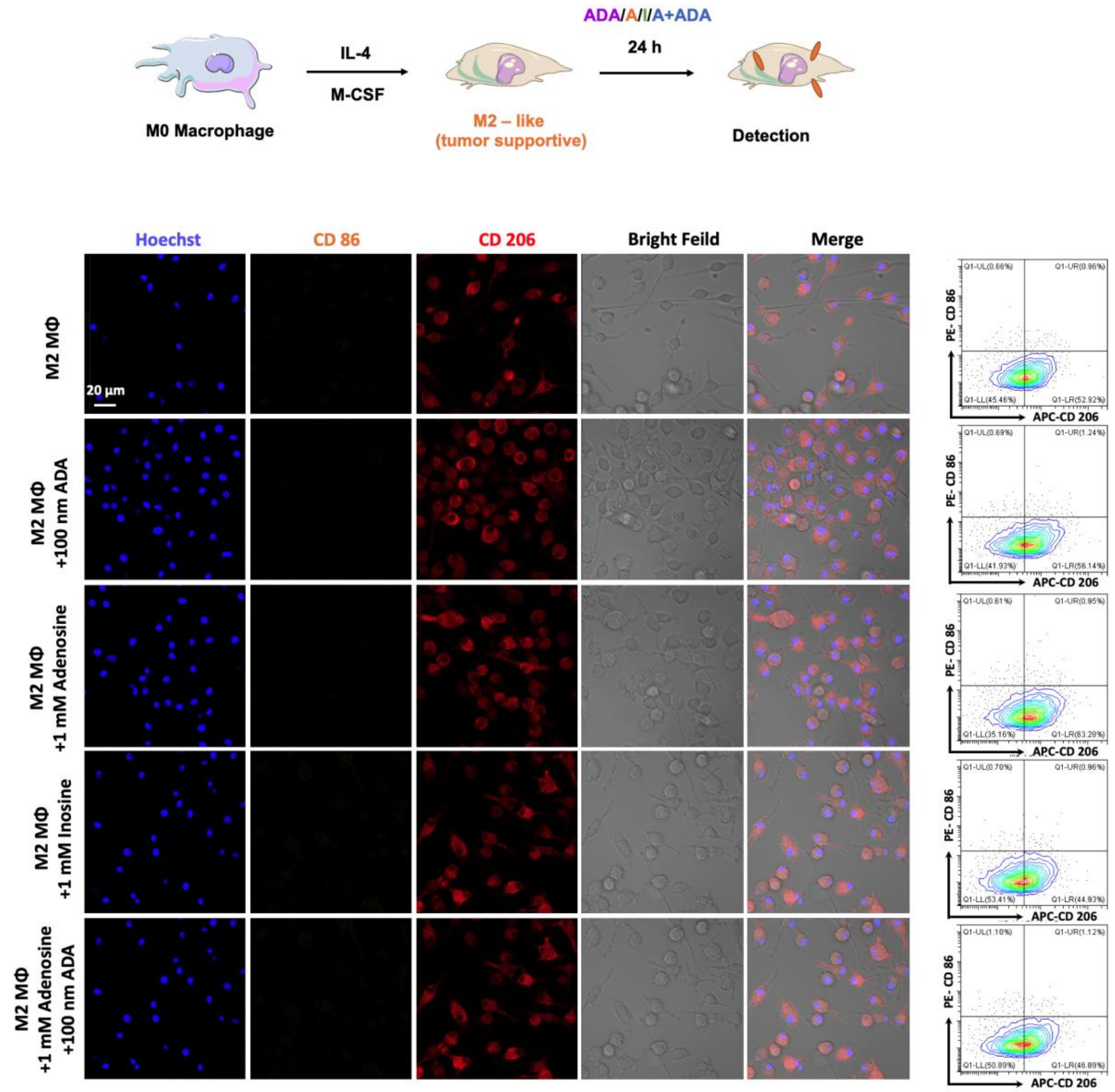

Supplementary Fig. 5 Immunofluorescence staining was conducted to visualize M2 macrophages in various treatment groups. Cell nuclei were stained with Hoechst (blue), M1 macrophages were stained with PE-CD86 (yellow), and M2 macrophages were stained with APC-CD206 (red). Flow cytometry analysis was performed to quantify the proportions of M1 and M2 macrophages in different treatment groups using co-staining with PE-CD86 and APC-CD206. All experiments were performed in triplicate (n=3) and the data are expressed as mean ± s.d.

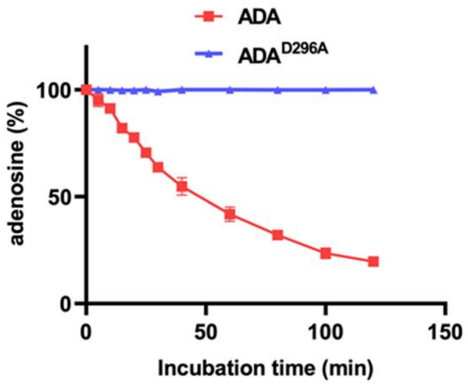

Supplementary Fig. 6 The adenosine conversion capability of ADA and mutant ADA^{D296A} were quantified by HPLC. All experiments were performed in triplicate, and data are presented as mean ± s.d.

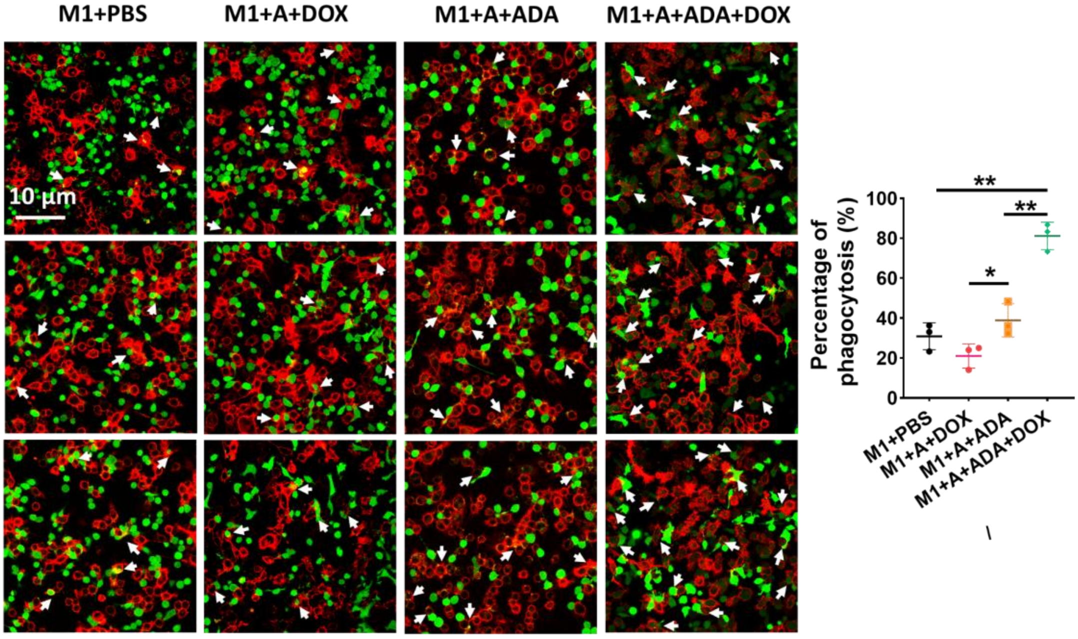

Supplementary Fig. 7 Immunofluorescence imaging was used to visualize the modulation of macrophage phagocytic capacity in response to various treatment as indicated. MC38 cell was pre-incubated with 10 ng/mL fluorescein diacetate. While M1 macrophages were labeled with APC anti-mouse F4/80 antibody. All experiments were performed in triplicate, and data are presented as mean ± s.d. Statistical differences between groups were assessed using one-way ANOVAs followed by Bonferroni's correction for multiple comparisons. *$p < 0.05$, **$p < 0.01$.

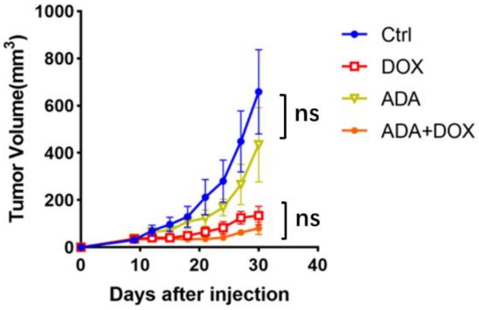

Supplementary Fig. 8 Tumor volume changes of MC38 tumor-bearing mice after different treatments were plotted (n=5). 100 μg ADA was administered via subcutaneous injection, while 70 μg DOX was administered through intravenous injection.

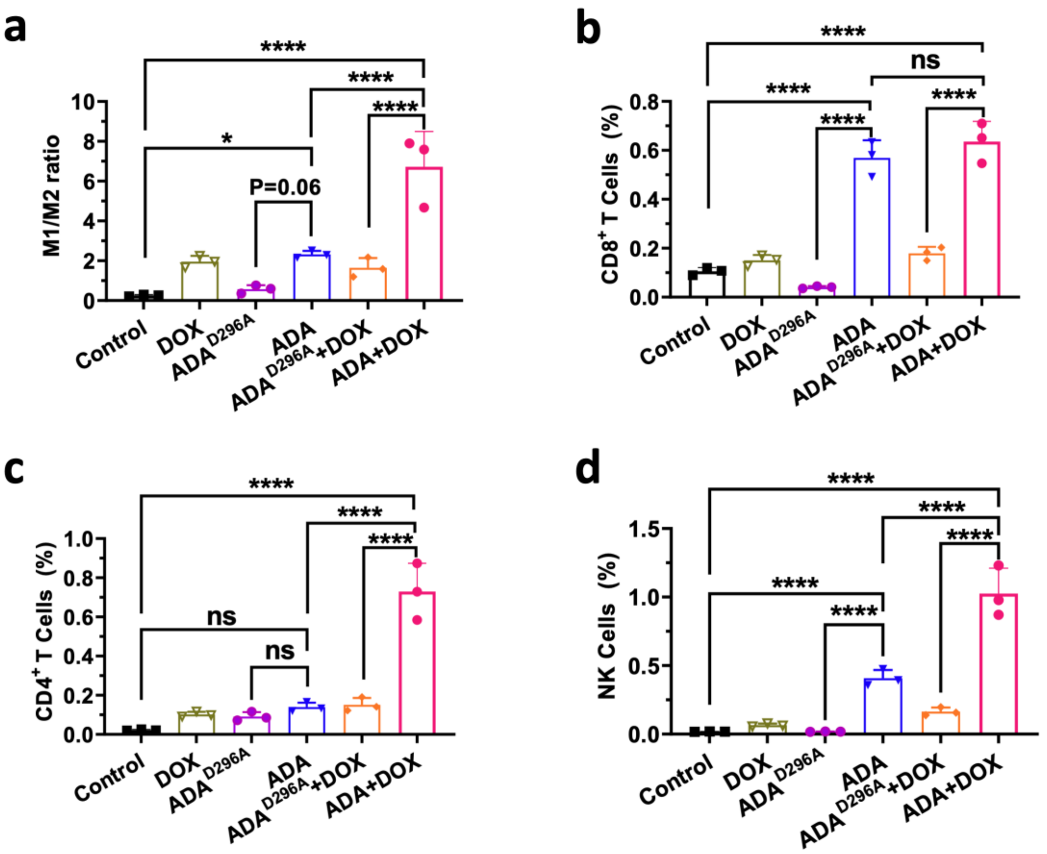

Supplementary Fig. 9 Quantitative analysis of (**a**) the M1/M2 macrophage ratio and measurement of the percentage of infiltrated (**b**) CD8$^+$ T cells, (**c**) CD4$^+$ T cells, and (**d**) NK cells in immunohistochemically stained murine tumor sections. All experiments were performed in triplicate, and data are presented as mean ± s.d. Statistical differences between groups were assessed using one-way ANOVAs followed by Bonferroni's correction for multiple comparisons. Significance were indicated as ns, not significant, *$p < 0.05$, **$p < 0.01$, ***$p < 0.001$, and ****$p < 0.0001$.

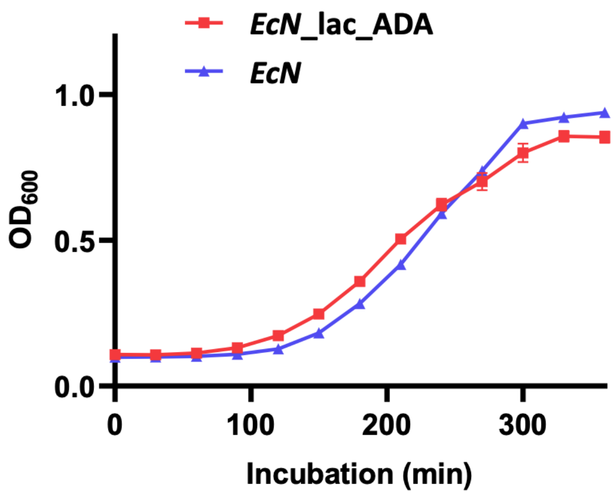

Supplementary Fig. 10 The growth curves of wild-type *E. coli* Nissle 1917 (*Ec*N) and engineered *Ec*N expressing surface-displayed adenosine deaminase (*Ec*N_ADA) were plotted.

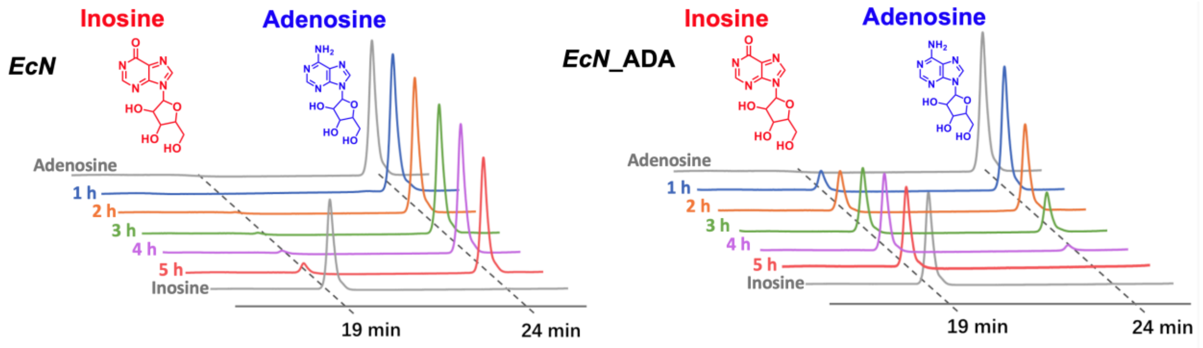

Supplementary Fig. 11 The HPLC profiles of adenosine and inosine were plotted after incubating wild-type *E. coli* Nissle 1917 (*Ec*N) and engineered *Ec*N expressing surface-displayed adenosine deaminase (*Ec*N_ADA) in HEPES buffer containing 1 mM adenosine for 5 hours.

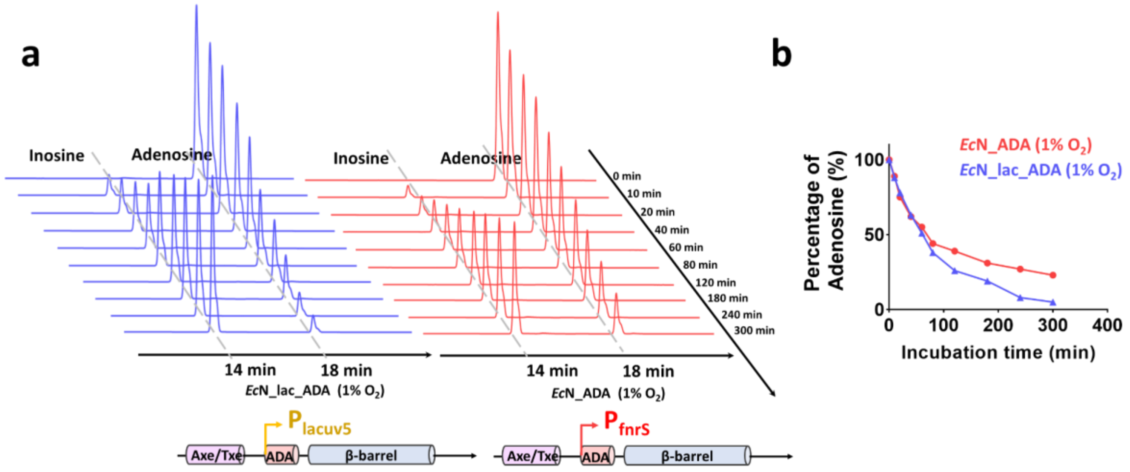

Supplementary Fig. 12 a. The HPLC profiles of adenosine and inosine were analyzed after incubation with *Ec*N_lac_ADA and *Ec*N_ADA under hypoxia conditions (1% O_2) in HEPES buffer containing 1 mM adenosine for 5 hours. **b.** The catalytic rate was quantified based on the reduction in adenosine peak area measured by HPLC.

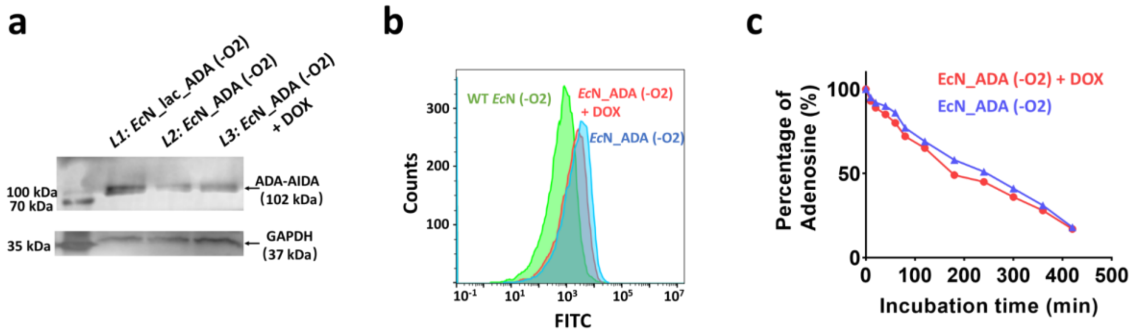

Supplementary Fig. 13 Western blot (a), flow cytometry (b), and enzymatic activity assays (c) were used to investigate the effects of DOX on ADA expression, surface display, and catalytic activity under hypoxia conditions.

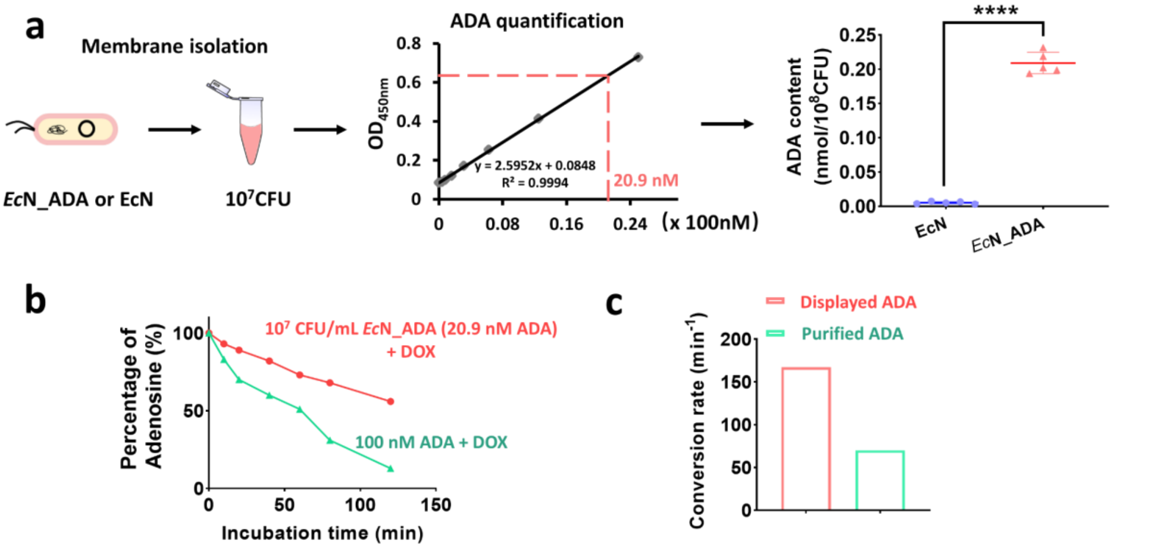

Supplementary Fig. 14 a. Quantification of the surface-displayed ADA enzyme on *Ec*N_ADA using ELISA. **b.** Catalytic activity of 10^7 CFU/mL *Ec*N_ADA and 100 nM ADA in the presence of 400 μM DOX, measured by HPLC. **c.** Calculated adenosine conversion rates for surface-displayed ADA and purified ADA enzyme.

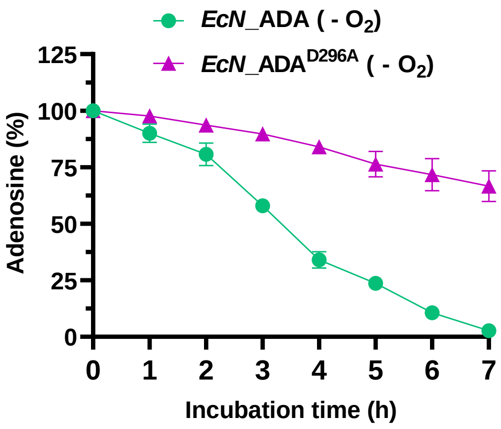

Supplementary Fig. 15 The adenosine conversion capability of engineered *Ec*N expressing surface-displayed adenosine deaminase (*Ec*N_ADA) and mutant (*Ec*N_ADA^{D296A}) under hypoxia condition were quantified by HPLC. All experiments were performed in triplicate, and data are presented as mean ± s.d.

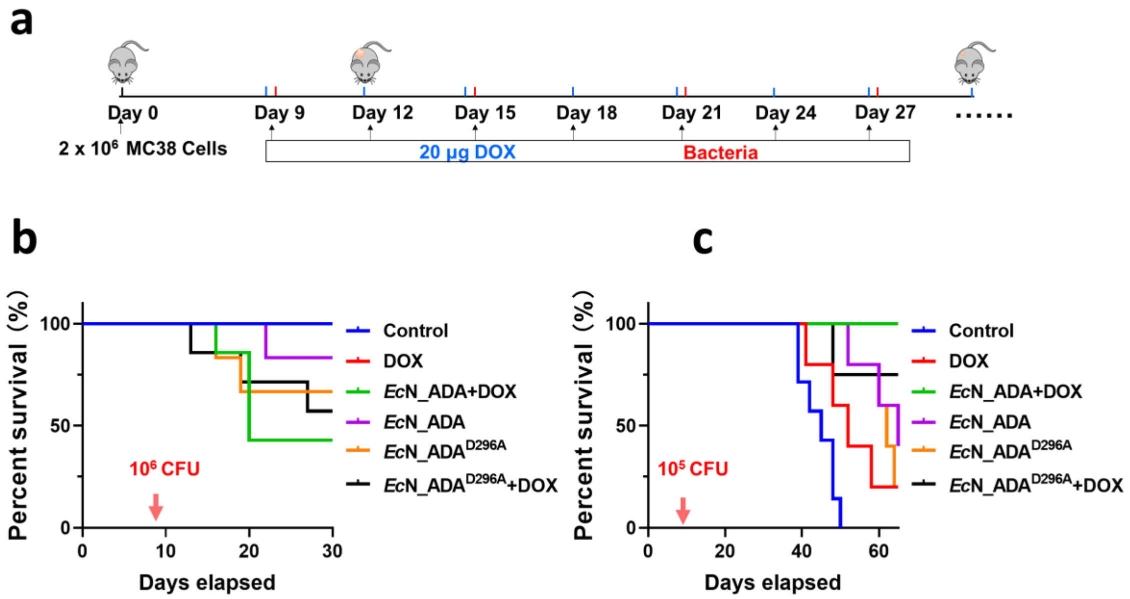

Supplementary Fig. 16 a. Schematic illustration of the therapy using engineered probiotics in the mouse MC38 subcutaneous tumor model. Survival curves of mice injected intravenously with 10^6 CFU (**b)** and 10^5 CFU (**c**) of engineered probiotics.

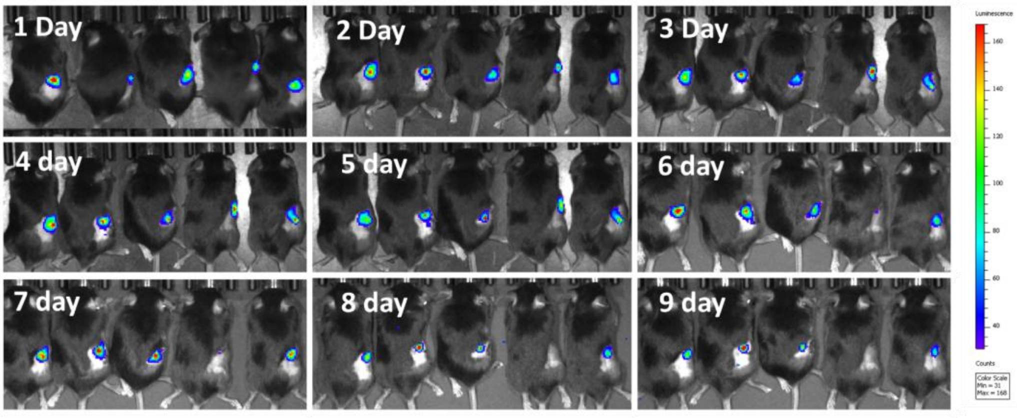

Supplementary Fig. 17 Bioluminescence imaging was used to track bacterial presence in the subcutaneous tumors of mice after intravenous administration over 9 days. A total of 10^5 CFU of *Ec*N_Lux_ADA was administered intravenously, and bioluminescence imaging tracking was performed daily.

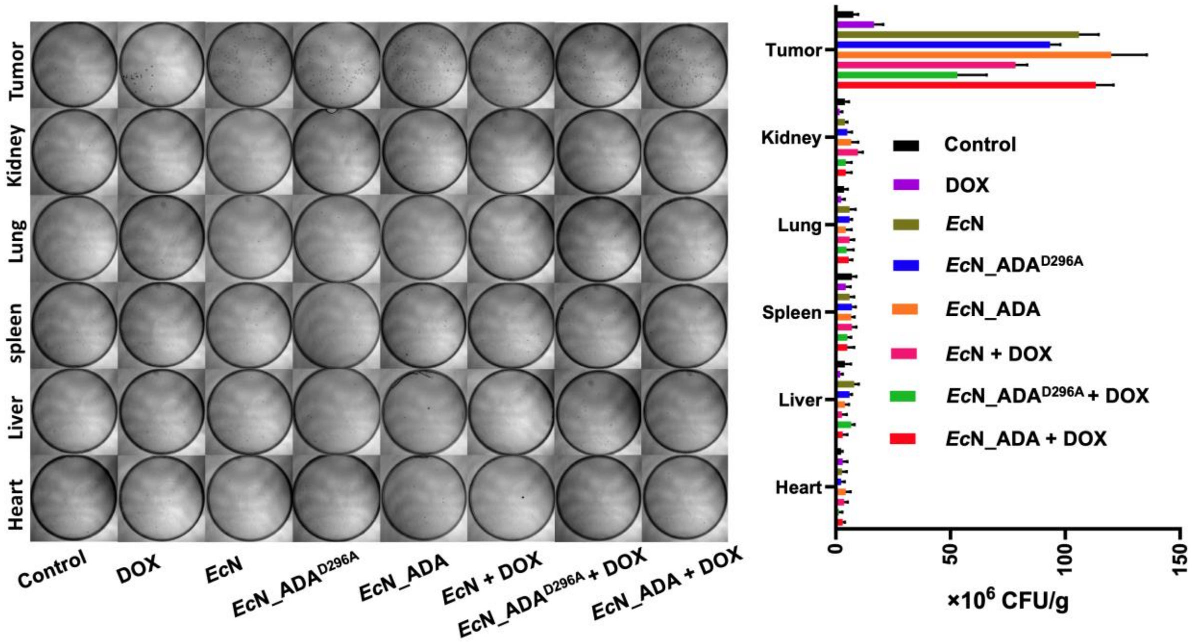

Supplementary Fig. 18. Quantitative assessment of bacterial colonization in the heart, liver, spleen, lungs, kidneys, and tumor tissues of mice from different treatment groups was performed using the plate spreading method.

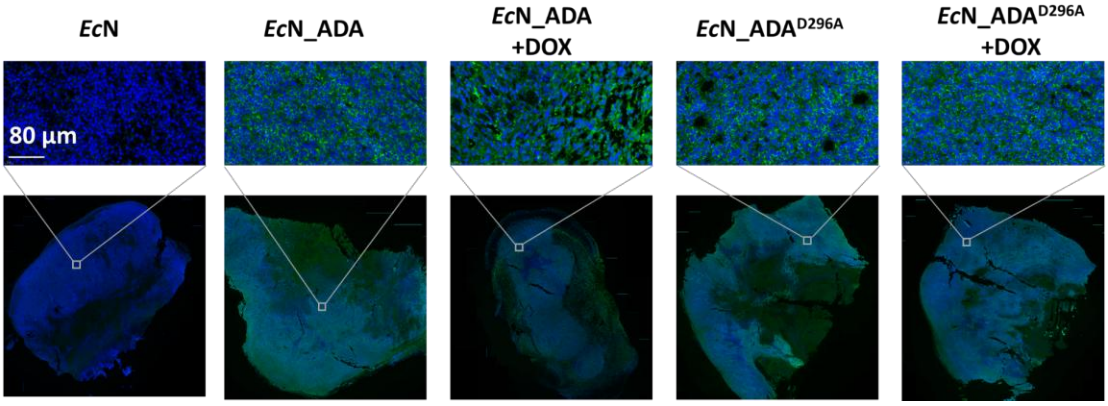

Supplementary Fig. 19 Fluorescent staining of mouse tumor section samples with FITC-anti-Myc antibody confirmed the *in situ* expression of ADA or ADAD296A by engineered probiotics in the tumor microenvironment. Tumor cells were stained blue, while bacteria expressing the Myc tag on their membrane surface were stained green. Scale bar: 80 μm.

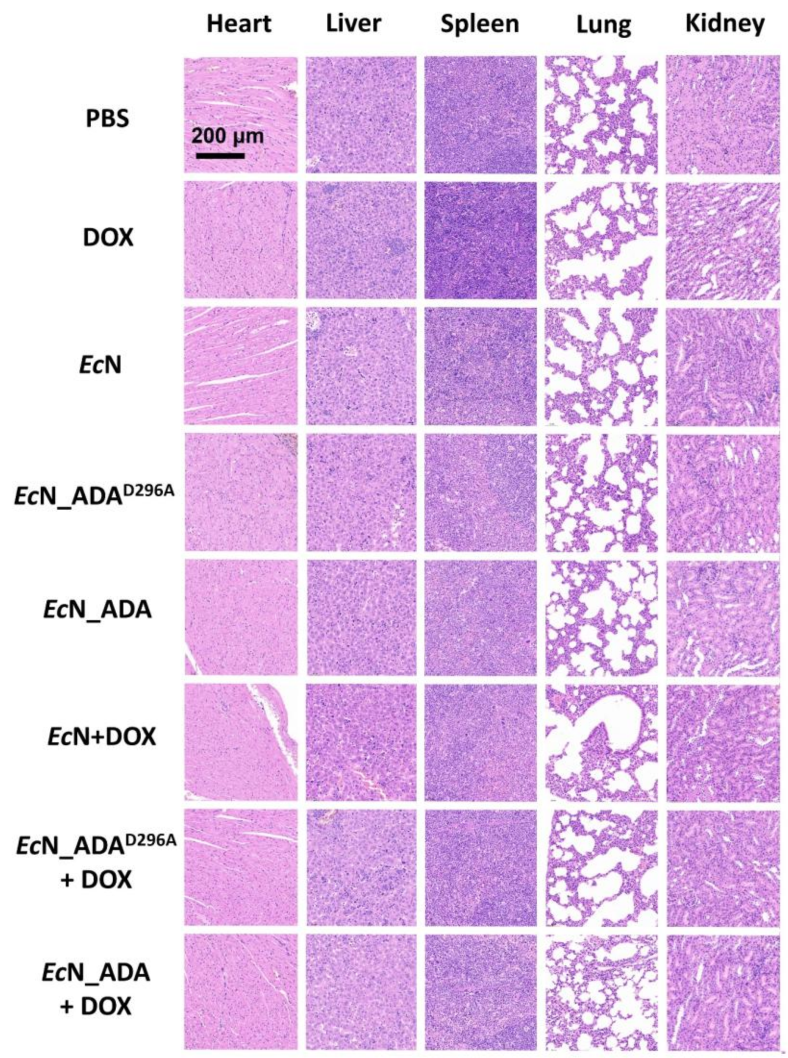

Supplementary Fig. 20 Immunohistochemistry (IHC) analysis on the heart, liver, spleen, lungs, and kidneys from mice in different treatment groups

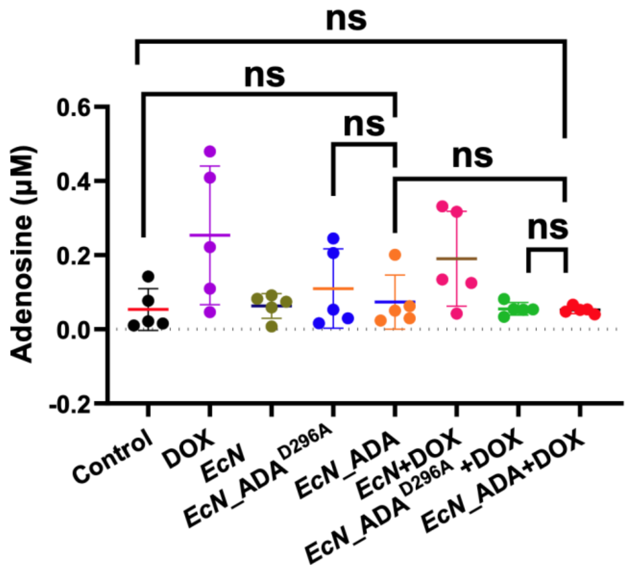

Supplementary Fig. 21 The concentrations of adenosine in murine tumor tissues from various treatment groups were quantified. All experiments consisted of five replicates;, and data are presented as mean ± s.d. Statistical differences between groups were evaluated using one-way ANOVAs followed by Bonferroni's correction for multiple comparisons. ns: not significant

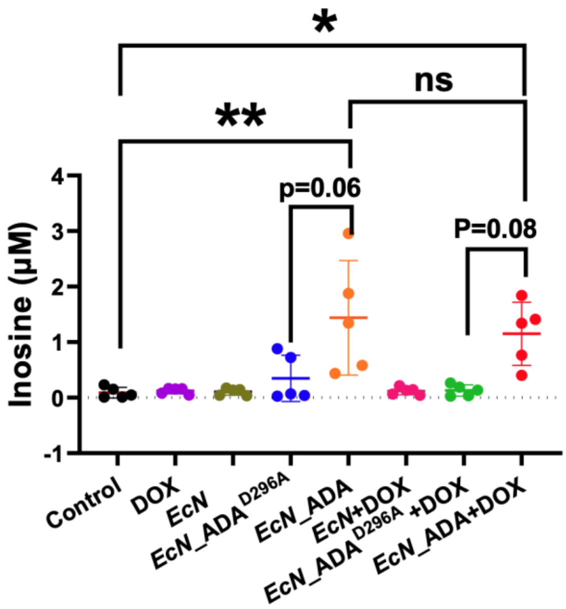

Supplementary Fig. 22 The concentrations of inosine in murine tumor tissues from various treatment groups were quantified. All experiments consisted of five replicates; and data are presented as mean ± s.d. Statistical differences between groups were evaluated using one-way ANOVAs followed by Bonferroni's correction for multiple comparisons. ns, not significant, * $p < 0.05$, ** $p < 0.01$.

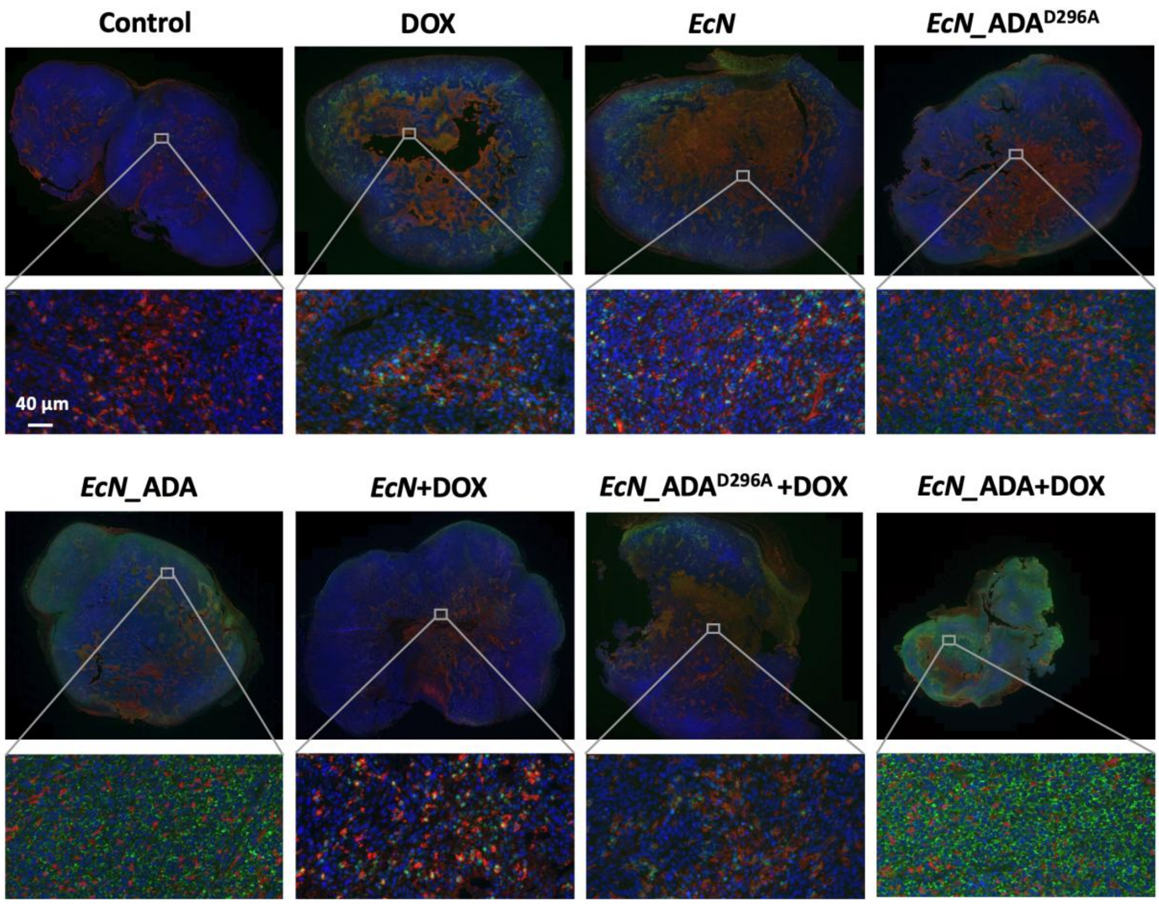

Supplementary Fig. 23 Immunohistochemical staining of tumor sections was performed to visualize the infiltration of M1 and M2 macrophages. M1 macrophages were stained in green, M2 macrophages were stained in red, and the nuclei were stained in blue. Scale bar = 40 μm.

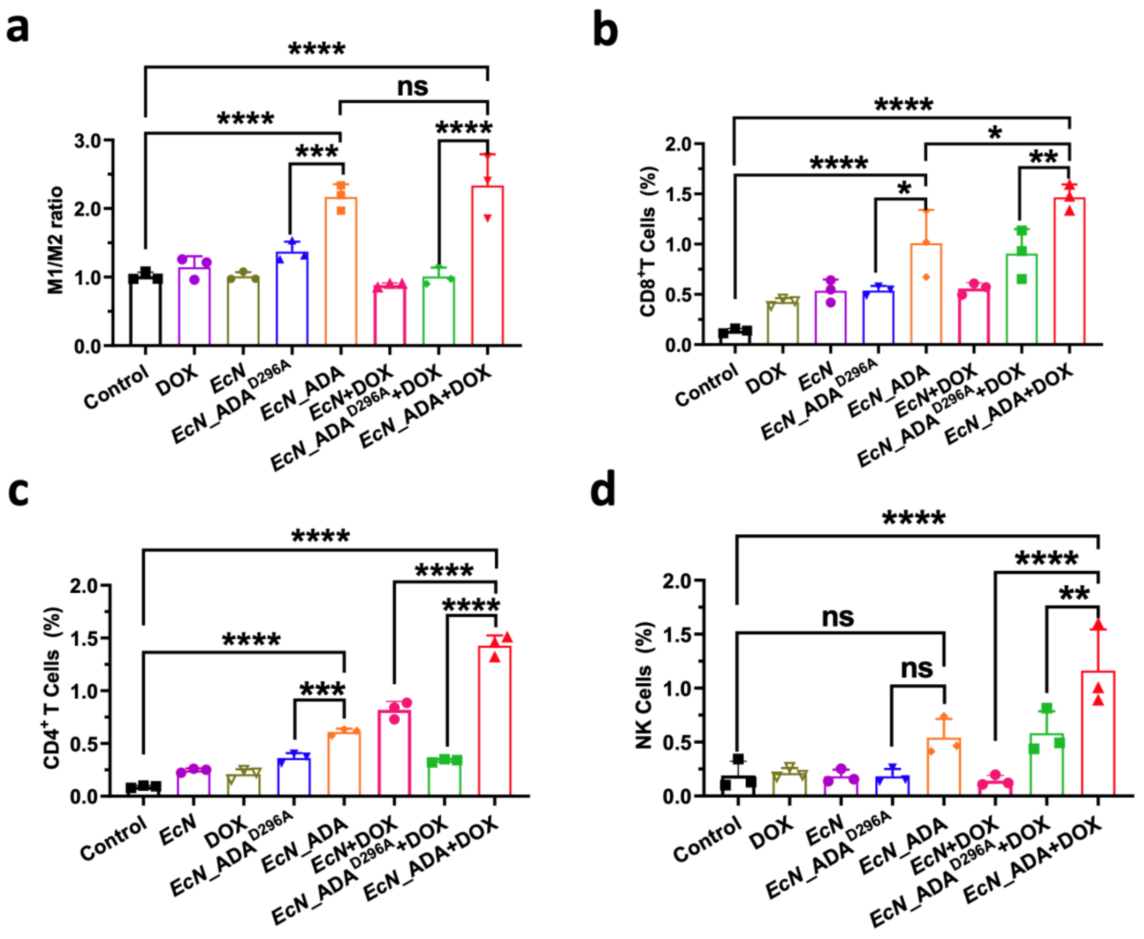

Supplementary Fig. 24 Quantitative analysis of (**a**) the M1/M2 macrophage ratio and measurement of the percentage of infiltrated (**b**) $CD8^+$ T cells, (**c**) $CD4^+$ T cells, and (**d**) NK cells in immunohistochemically stained murine tumor sections. All experiments were performed in triplicate, and data are presented as mean ± s.d. Statistical differences between groups were assessed using one-way ANOVAs followed by Bonferroni's correction for multiple comparisons. Significance were indicated as ns, not significant, *$p < 0.05$, **$p < 0.01$, ***$p < 0.001$, and ****$p < 0.0001$.

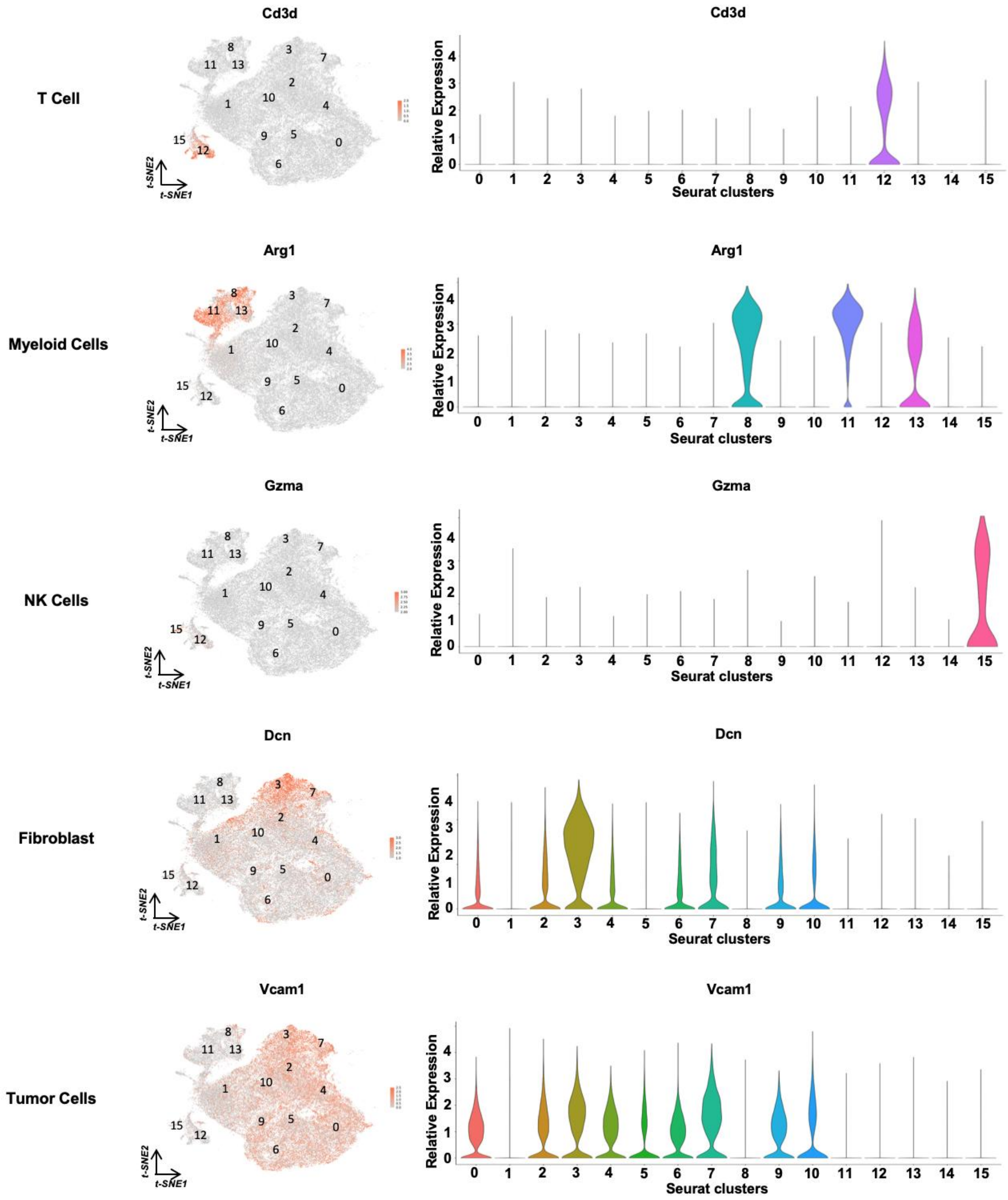

Supplementary Fig. 25 The single-cell RNA gene transcription data were processed and sorted into 16 clusters, visualized in a t-distributed stochastic neighbor embedding (T-SNE) plot. The plot illustrates the expression of curated feature genes used for identifying cell populations such as T cells, Myeloid Cells, NK Cells, Fibroblasts, and Tumor Cells. Expression levels of individual markers are depicted as color gradients from grey (low expression) to orange (high expression). Violin plots were generated to show the expression distribution of typical cell marker genes (*Cd3d*, *Arg1*, *Gzma*, *Dcn and Vcam1*) across each cluster.

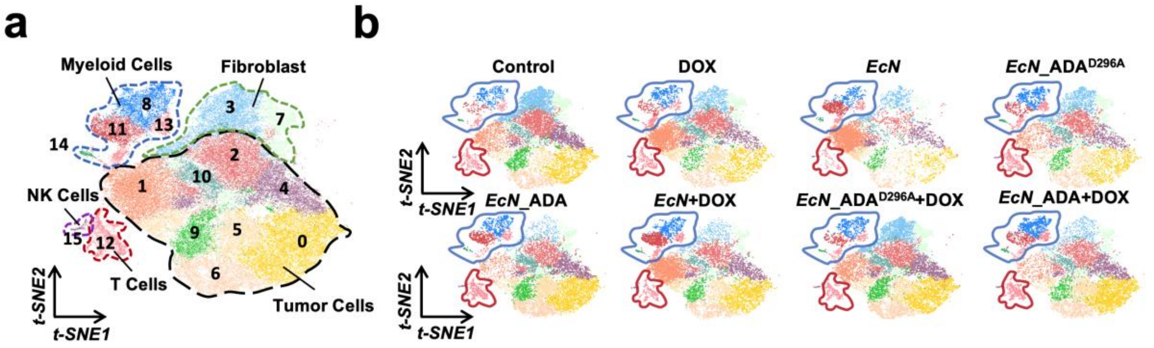

Supplementary Fig. 26 (a) A T-SNE plot illustrating the 5 identified cell populations, namely tumor cells, fibroblasts, myeloid cells, NK cells, and T cells, sorted into clusters. **(b)** T-SNE projections separated by condition, with myeloid cells highlighted in blue and T cells highlighted in red.

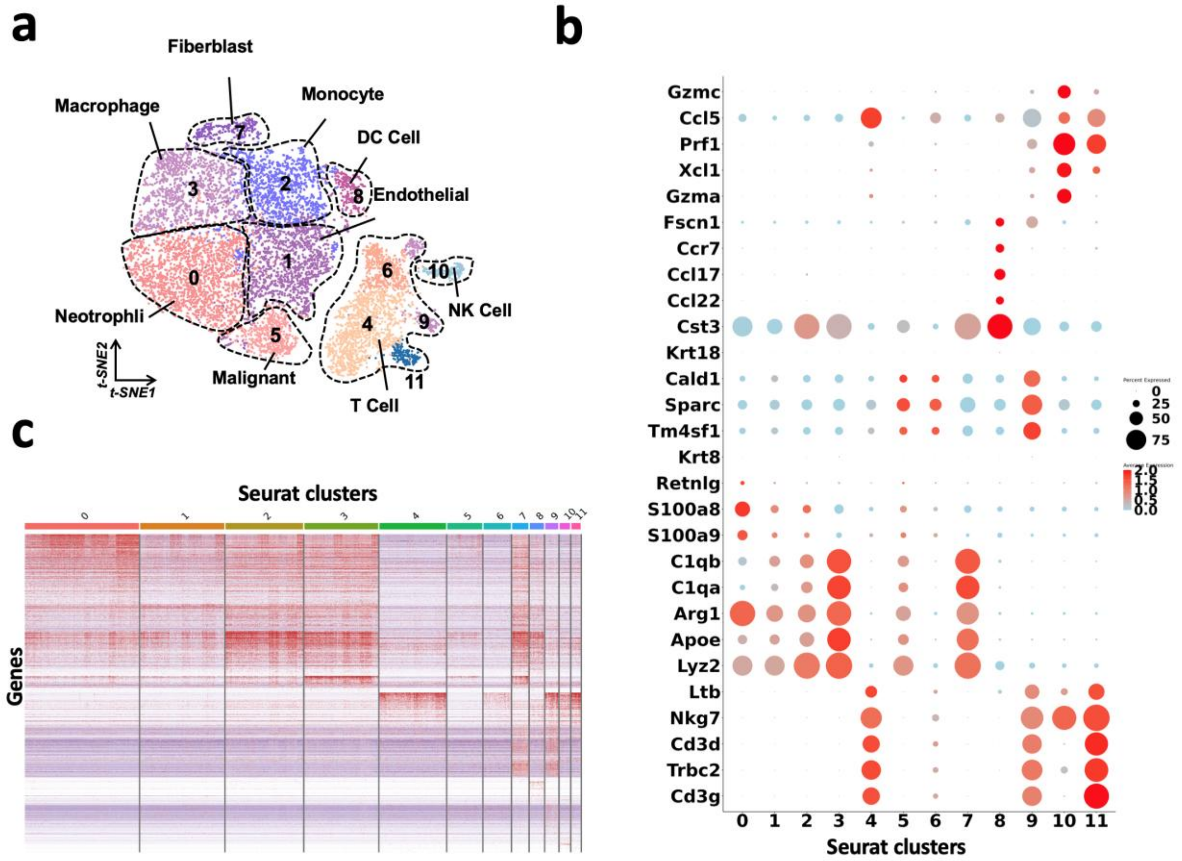

Supplementary Fig. 27 (a) A T-SNE plot depicting the 9 identified cell populations, including T cells, NK cells, dendritic cells (DCs), monocytes, macrophages, neutrophils, fibroblasts, endothelial cells, and malignant cells, after sub-setting and re-clustering of all immune cells. **(b)** Known markers used for clustering analysis of immune cells, where the size of markers reflects the percentage of each cluster expressing a given gene, with average scaled expression indicated on a color gradient. **(c)** Heatmap showing the expression of all marker genes across 12 clusters, with average scaled expression represented on a color gradient.

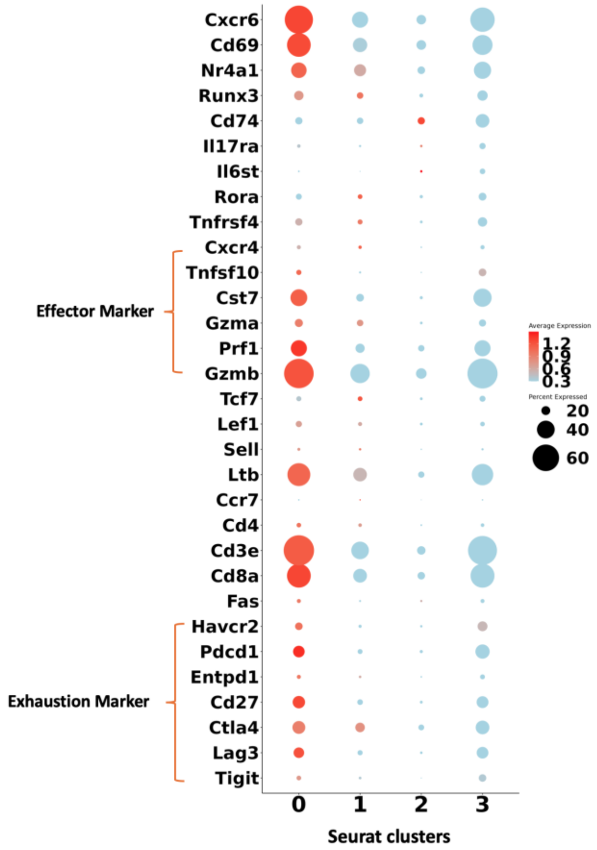

Supplementary Fig. 28 Known markers for clustering analysis of T cells are shown. The size of each marker reflects the percentage of each cluster expressing the respective gene, and the average scaled expression is indicated on a color gradient.

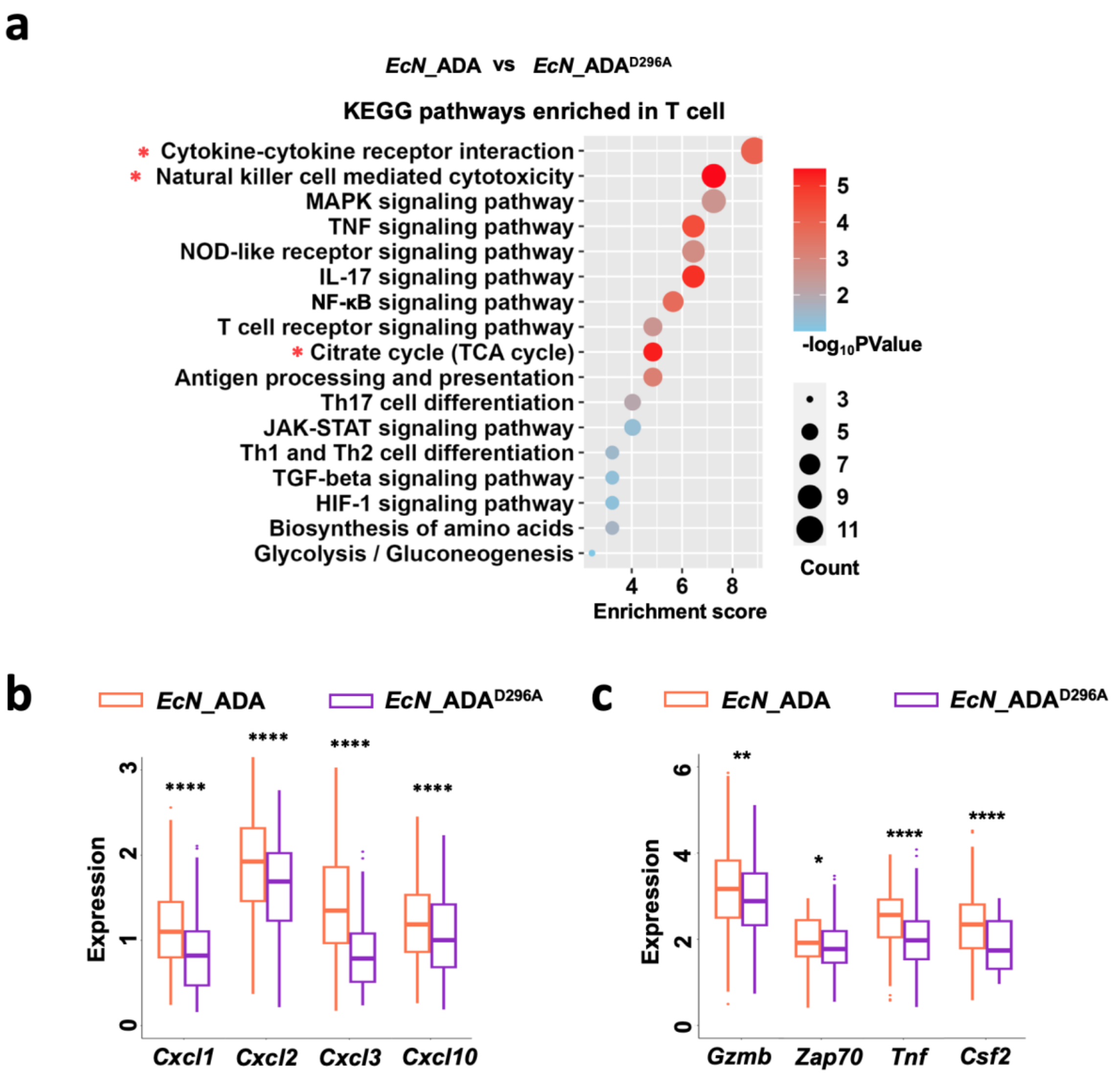

Supplementary Fig. 29 (a) KEGG pathway enrichment analysis was performed on T cells between *Ec*N_ADA and *Ec*N_ADA^{D296A}-treated group. **(b)** A boxplot illustrating the gene expression in the cytokine-cytokine receptor interaction pathway within T cells between the *Ec*N_ADA and *Ec*N_ADA^{D296A}-treated group. **(c)** A boxplot demonstrating the expression of genes related to the natural killer cell-mediated cytotoxicity pathway within T cells between the *EcN*_ADA and *EcN*_ADA^{D296A}-treated group. Statistical analyses were performed using the Gehan-Wilcoxon test followed by Bonferroni's correction for multiple comparisons for all survival analyses. Significance levels were denoted as *p < 0.05, **p < 0.01, and ****p < 0.0001.

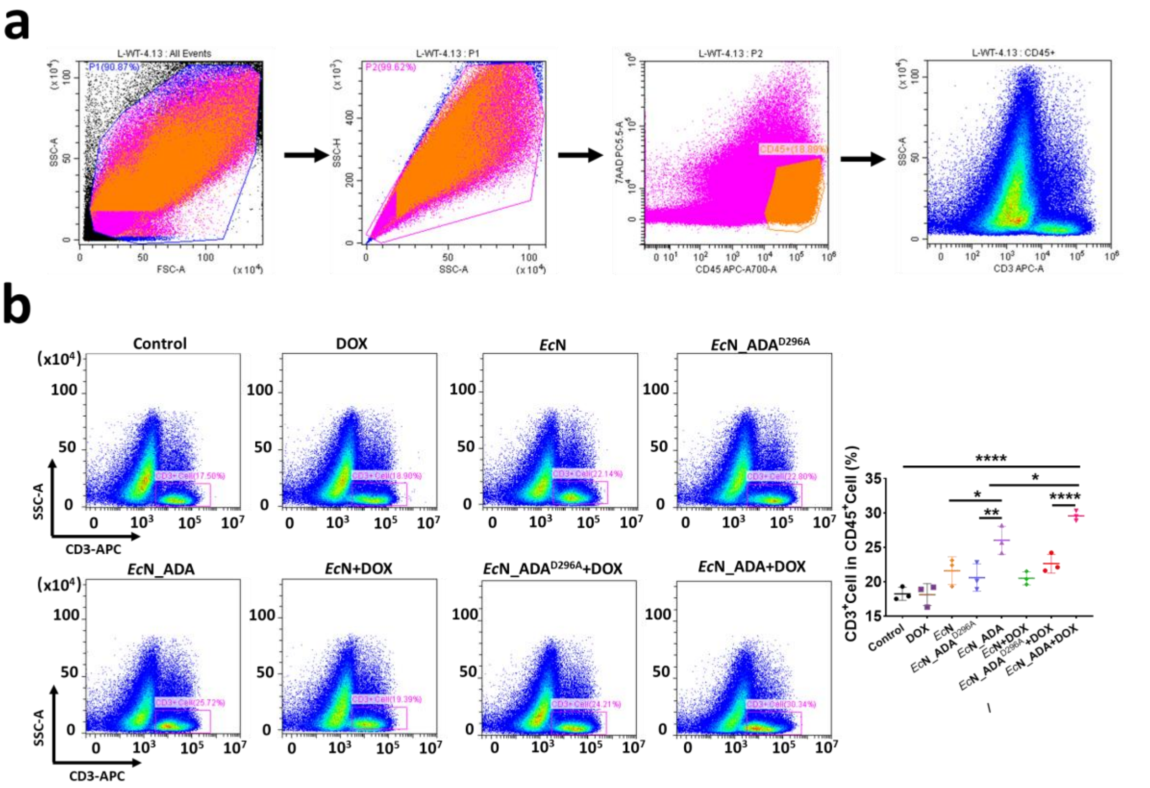

Supplementary Fig. 30 Flow cytometry analysis depicting the enhanced penetration of T cells in the *Ec*N_ADA + DOX treatment group. **a.** The protocol for $CD3^+$ T cell subset analysis. **b**. Two-dimensional flow cytometry plots showing the infiltration levels of $CD3^+$ T cells in different treatment groups.

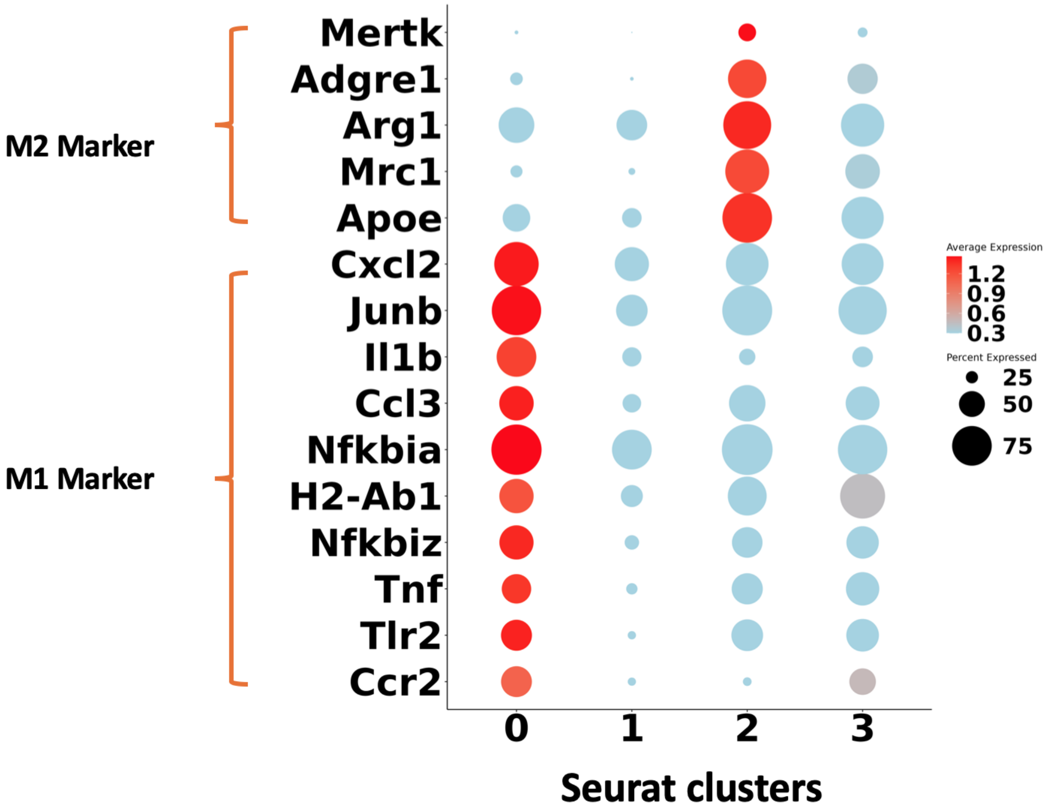

Supplementary Fig. 31 Known markers for clustering analysis of macrophages are displayed. The size of each marker indicates the percentage of each cluster expressing the respective gene, with the average scaled expression shown on a color gradient.

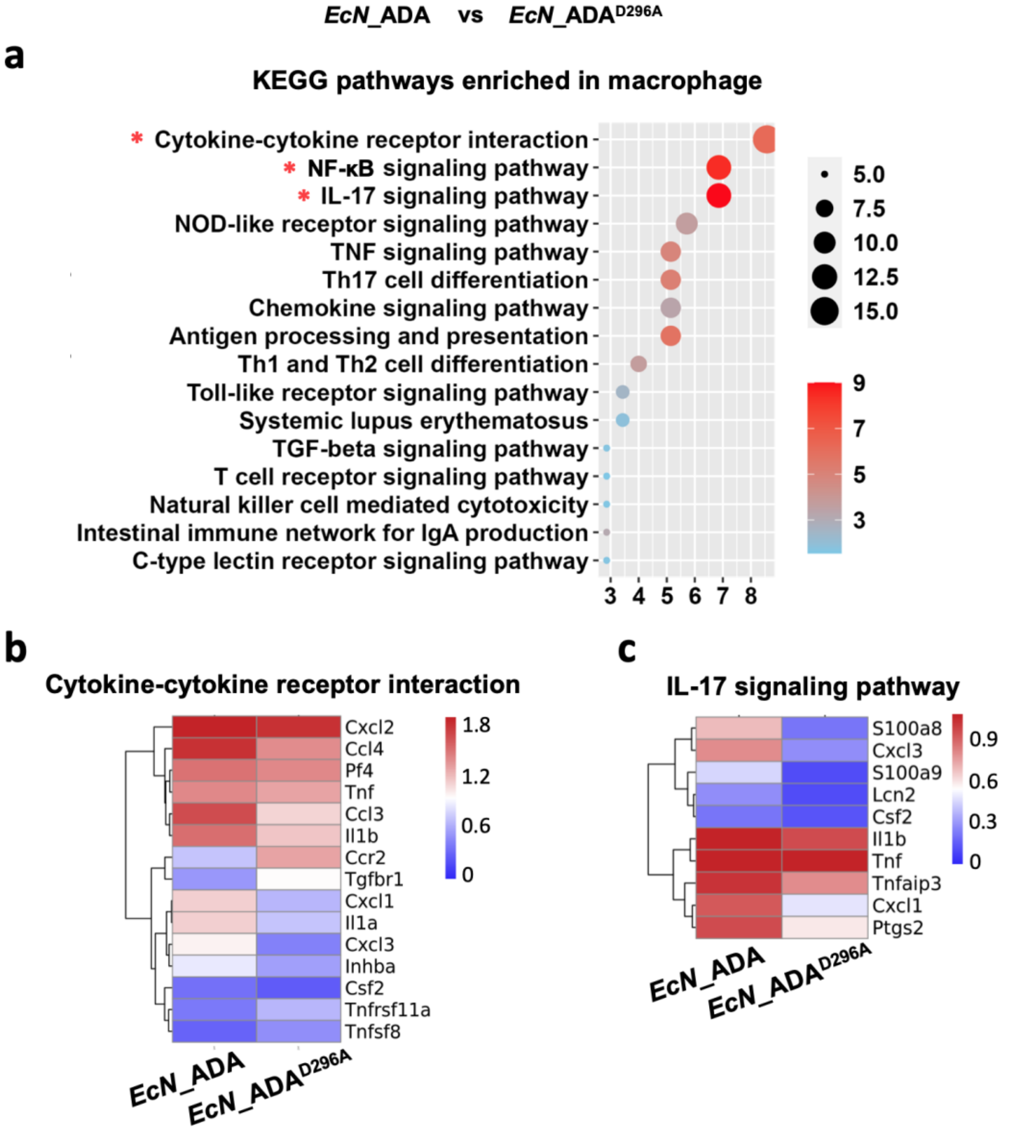

Supplementary Fig. 32 (a) KEGG pathway enrichment analysis was performed on macrophage between the *Ec*N_ADA and *Ec*N_ADA^{D296A}-treated group. **(b)** Heatmap depicting the gene expression levels in the cytokine-cytokine receptor interaction pathway within macrophages between the *Ec*N_ADA and *Ec*N_ADA^{D296A}-treated group. **(c)** Boxplot illustrating the expression of genes related to the natural killer cell-mediated cytotoxicity pathway within macrophages between the *Ec*N_ADA and *Ec*N_ADA^{D296A}-treated group.

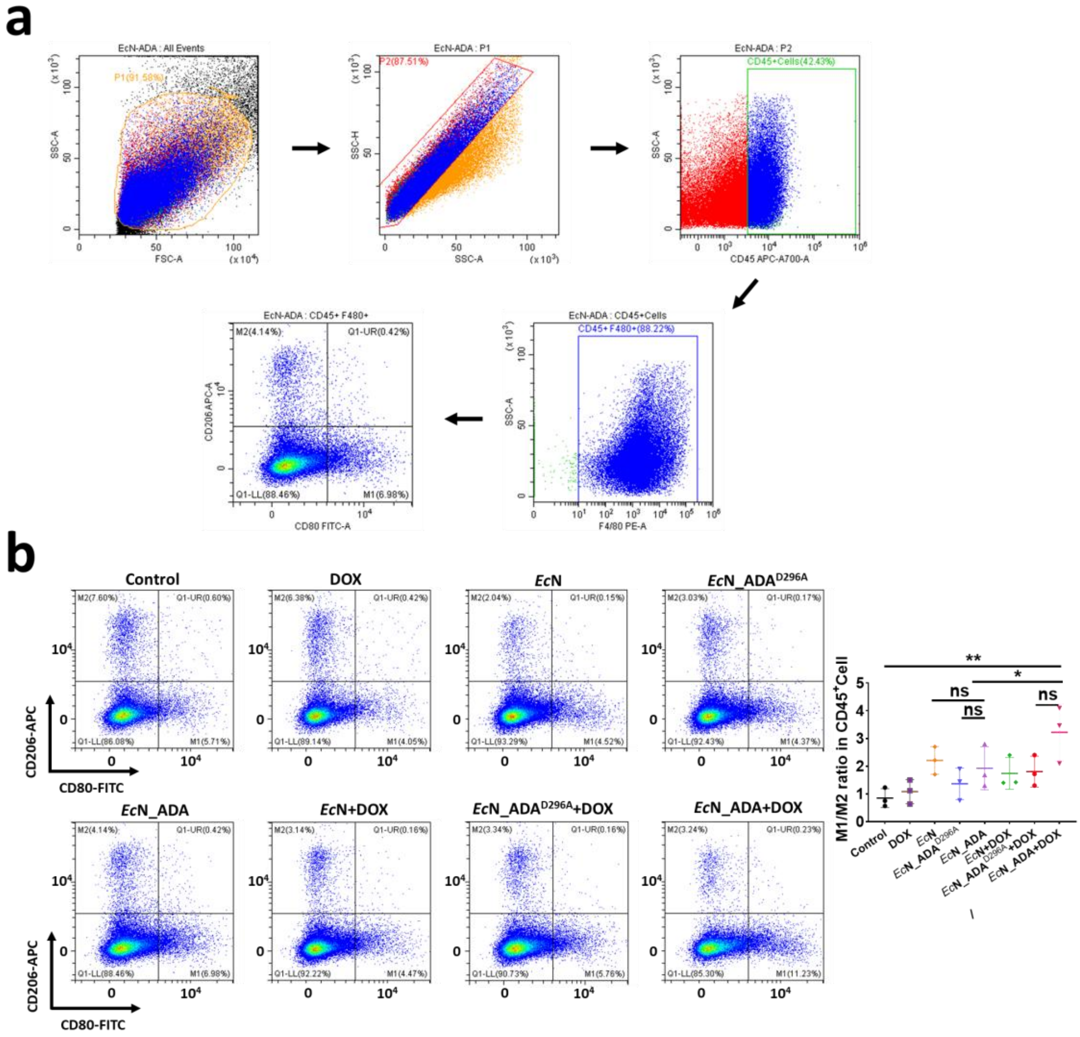

Supplementary Fig. 33 Flow cytometry analysis depicting the shift from M1 to M2 microphages in the *Ec*N_ADA + DOX treatment group. **a.** The protocol for macrophage subset analysis. **b**. Two-dimensional flow cytometry plots showing the ratio of M1 and M2 macrophages in different treatment groups.

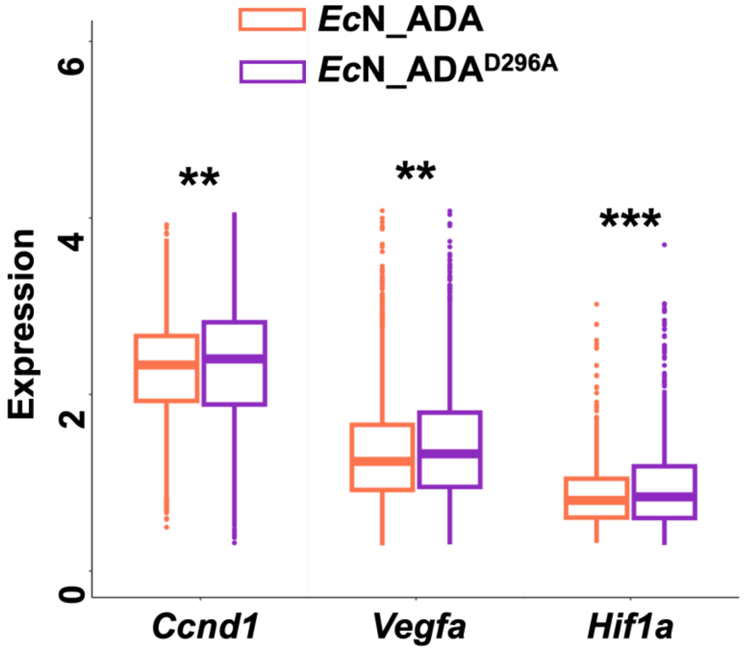

Supplementary Fig. 34 The average expression of genes (*Ccnd1*, *Vegfa*, and *Hif1a*) in the *Ec*N_ADA and *Ec*N_ADA^{D296A}-treated groups were compared. Statistical analyses were conducted using the Gehan-Wilcoxon test followed by Bonferroni's correction for multiple comparisons for all survival analyses. Significance levels were denoted as *p < 0.05, **p < 0.01, ***p < 0.001, and ****p < 0.0001.

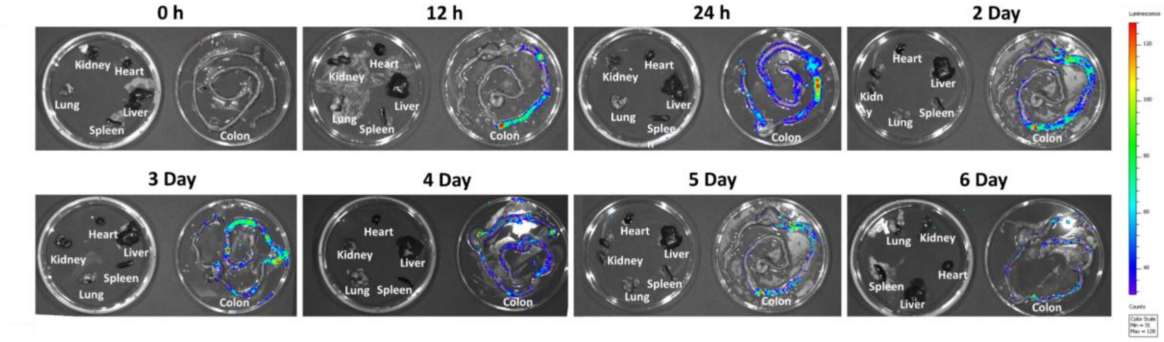

Supplementary Fig. 35 Bioluminescence imaging was used to track bacterial presence in the colons of mice after oral administration over 9 days. A total of 10^9 CFU of *Ec*N_Lux_ADA was administered orally, and bioluminescence imaging tracking was performed. After euthanasia, the colons, along with the heart, liver, spleen, lungs, and kidneys, were harvested for further analysis.

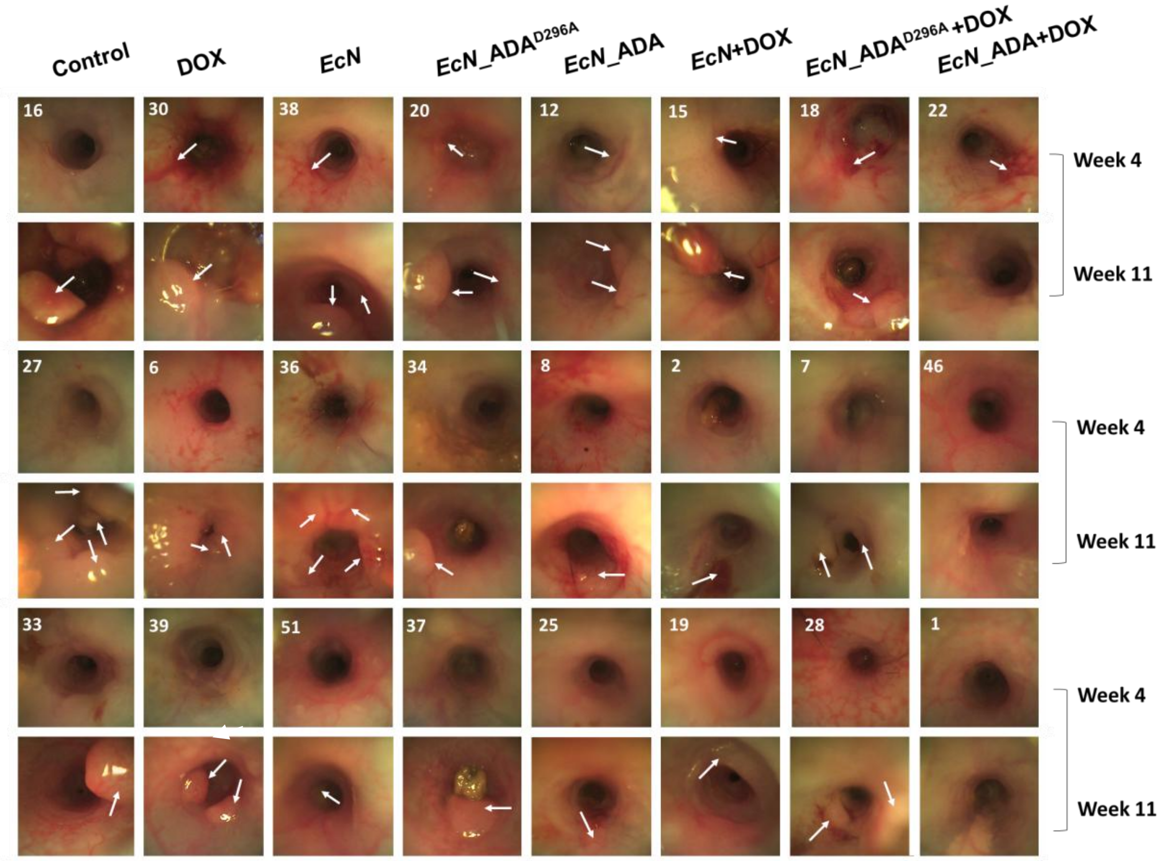

Supplementary Fig. 36 Colonoscopy examinations were conducted at weeks 4 and 11 for different treatment groups. Three murine colonoscopy photographs were displayed for each treatment group. The numbers for the mice are labeled and the tumors are indicated by white arrows.

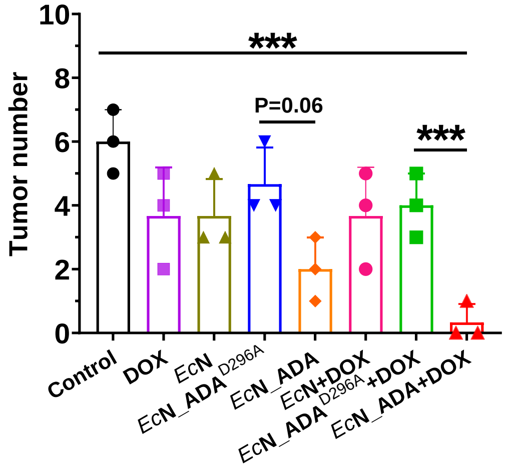

Supplementary Fig. 37 The number of intestinal tumors observed in colonoscopy examinations of mice from different treatment groups. Statistical differences between groups were assessed using one-way ANOVA followed by Bonferroni's correction for multiple comparisons. Significance levels were denoted as ***$p < 0.001$.

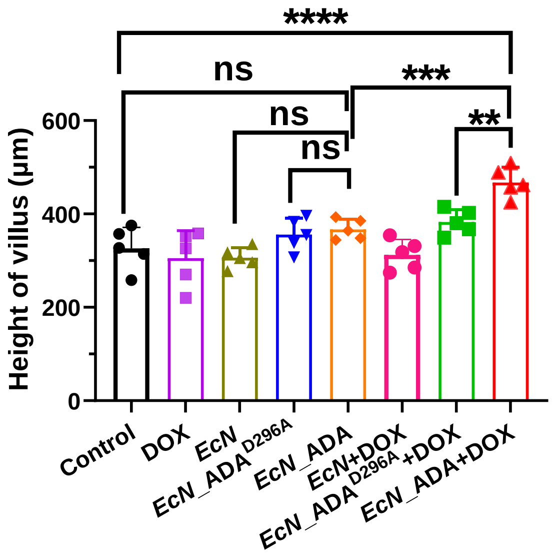

Supplementary Fig. 38 The height of villi in the mouse colon was measured across different treatment groups. Statistical differences between groups were assessed using one-way ANOVAs followed by Bonferroni's correction for multiple comparisons. Significance levels were denoted as *p < 0.05, **p < 0.01, ***p < 0.001, and ****p < 0.0001.

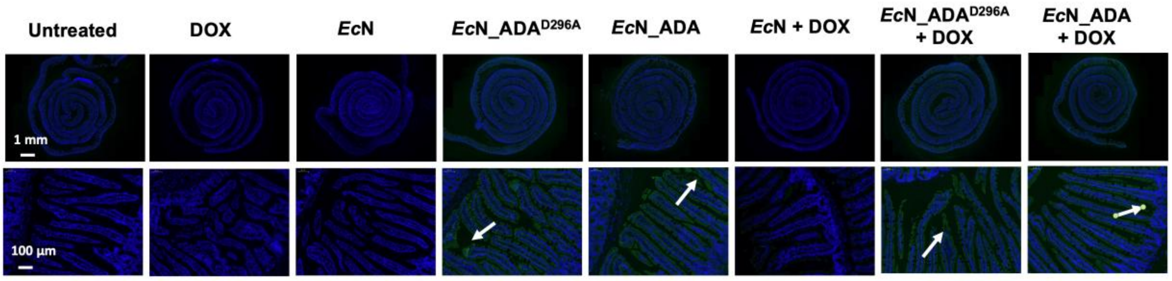

Supplementary Fig. 39 Immunofluorescence staining with anti-Myc-FITC antibodies was performed to visualize the distribution of engineered bacteria in the mouse colon. The labeled bacteria in green are indicated by white arrows. The scale bar represents 100 µm.

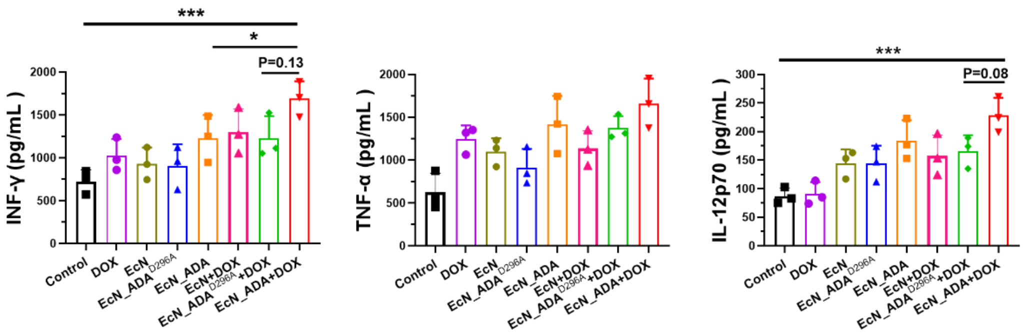

Supplementary Fig. 40 Secreted cytokine levels of IFN-γ, TNF-α, and IL-12p70 in murine serum from different experimental groups in a mouse orthotopic colorectal cancer model. Error bars represent the standard deviation of three mice per group (n=3). Statistical differences between groups were assessed using one-way ANOVA followed by Bonferroni's correction for multiple comparisons. Significance levels are denoted as *$p < 0.05$ and ***$p < 0.001$.

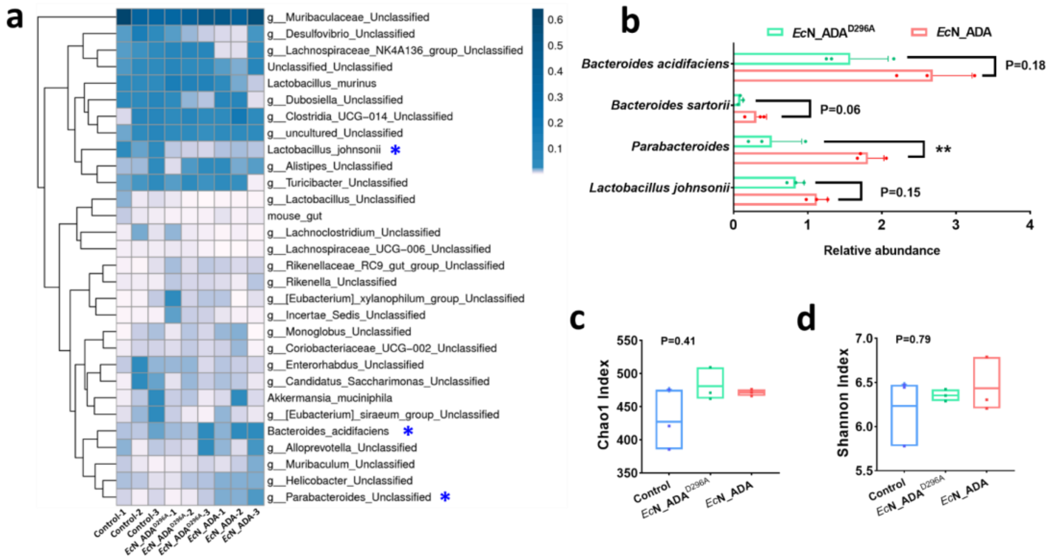

Supplementary Fig. 41 The effects of oral administration of engineered bacterial strains on the gut microbiota in mice. **a.** Heatmap illustrating the expression levels of different gut microbiota across various treatment groups. **b.** The abundance of *Parabacteroides*, *Bacteroides acidifaciens*, *Bacteroides sartorii*, and *Lactobacillus johnsonii* across different treatment groups. **c-d.** Chao1 index and Shannon index across different treatment groups.

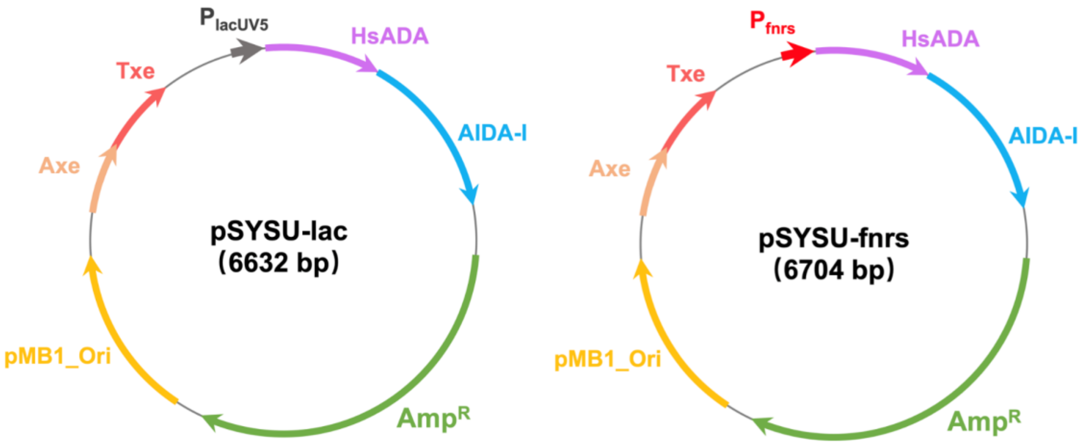

Supplementary Fig. 42 The plasmid maps of pSYSU-lac and pSYSU-fnrs.

Supplementary Table. S1

**Primers**	**Function**	**Sequence**
BamHI-ADA_fwd	PCR amplification of ADA sequence	AAAGGATCCGTATGGCGCAGACCCCGGC
SacI-ADA_rev		AAAGAGCTCTTACAGGTTCTGACCCGCGC
BamHI-*Bp*ADA_fwd	PCR amplification of *Bp*ADA sequence	AAAGGATCCGATGGTGGATGAAGCGATGGAAC
SacI-*Bp*ADA_rev		AAAGAGCTCTTACAGGCCGCGTTCCATCTG
BamHI-*Lj*ADA_fwd	PCR amplification of *Lj*ADA sequence	AAAGGATCCGATGAAAAACTTTATTGATCTGCATCTGC
SacI-*Lj*ADA_rev		AAAGAGCTCTTAGTTAAAAATCGCCAGCAGGCGGTTTT

Supplementary Table. S2

DNA sequence	Comments
cgatactctctcctttcatcgaaataaccctatgaaaaatatggctgcaatcatcttgaatagtag<br>actcacaagggctaatacaaagcgccaaaagaccaaacaaaataccagtattgccaaaaata<br>ctacgaagtttaaatgcacagctgcagcatggcgcataagtatttcgccaacaaataggactg<br>acaatagaggaatgattttattcatatggatctccttctttctcttaggttatcgggtgttgcccgctt<br>atttcgcaccacctaagcggcgggggcggttggagcgattggtgcgaaataagaaccattcc<br>cttagttactaggtttttttgtccttatttcacacattctcacttttcggtataaatgattattgcctttttttc<br>ctctcaaatcgttgatatggcattcgtttacggtcaagttcaggatccataagcgactaatgcca<br>attgcaataggaaataaacggttacccgttacgttggatgaaaagagacaaaaagaattgcag<br>caactaaagcagaagtacggcaaaagtgaatccaggattatgtgtattgcgttagatttattgatt<br>gcccaagaaaaagcaggatttgaggtaccagcactcaaaaagtgacgtcaccttttatcctaa<br>aaactaaaagtgatagcacttttaattataagaagttagaatattaatcatttgcttaattgtacaat<br>ataatgtacaattgttttatagaaataaataaggggtgaaaggaatggaagcagtagcttattca<br>aatttccgccaaaatttacgtagttatatgaaacaagttaatgaggatgctgaaacacttattgta<br>acaagtaaagatgtagaagatacagttgttgtattatcaaaaagagattatgattctatgcaaga<br>aacgttgagaacactttctaataattacgtcatggaaaaaattcgtcgaggagatgaacaattct<br>ccaaaggtgcatttaaaacacatgacttaatcgaggttgaatctgatgattaaggcttggtctgat<br>gatgcttgggatgattatctttattggcatgagcaaggaaacaaaagcaatataaaaaagattaa<br>caagttaataaaagatatcgatcgttccccctttgctggattaggaaaacctgagccattaaagc<br>atgatttatctggaaaatggtccagaagaattacagatgaacatagactgatatatagagttgaa<br>aatgaaacgatatttatttattctgcaaaagatcactattaaccaatcggaagtaaggaaagggt<br>cagaaacttaaaagtttttgatccttattttatttaccctagtcatttaaaaagctaatatagcttagt<br>gttgattgttattaatgaatgtgtttgttacgcgtattacggatataaggttagtaaaatcatttctaa<br>agttgaggaaaagtaaatataaatggcttaaatttcaacaatttgaagttgaatagatatgttataa<br>tactattgtagtgtgggatgttagttactaaggatgacgcttatatatatgactgaatagaataag<br>caataggtttaataatctattttaaattttttgtactagttttagtcaattagcaaaaacaacaaaaat<br>aaacttctcatagaatttagctaaaaattaatgatttatttacatattaaatttggatacagttaagta<br>atttttatatattggaggagaagtaatggaatataaatttaacttgaatttgaaagaagtatcgctg<br>ataccatggattctcactcattaggcaccccaggctttacactttatgcttccggctcgtataatgt<br>gtggaattgtgagcggataacaatttcacacaggaaagcttcatatgaataaggcctacagtat<br>catttggagccactccagacaggcctggattgtggcctcagagttagccagaggacatggtttt<br>gtccttgcaaaaaatacactgctggtattggcggttgtttccacaatcggaaatgcatttgcagtc<br>gacatggcgcagaccccggcgtttgataaaccgaaagtggaactgcatgtgcatctggatgg<br>cagcattaaaccggaaaccattctgtattatggccgccgtcgcggcattgcgctgccggcgaa<br>caccgcggaaggcctgctgaacgtgattggcatggataaaccgctgaccctgccggattttct<br>ggcgaaatttgattattatatgccggcgattgcgggctgccgcgaagcgattaaacgcattgc<br>gtatgaatttgtggaaatgaaagcgaaagaaggcgtggtgtatgtggaagtgcgctatagccc<br>gcatctgctggcgaacagcaaagtggaaccgattccgtggaaccaagcggaaggcgatctg<br>accccggatgaagtggtggcgctggtgggccaaggcctgcaagaaggcgaacgcgattttg<br>gcgtgaaagcgcgcagcattctgtgctgcatgcgccatcagccgaactggagcccgaaagt<br>ggtggaactgtgcaaaaaatatcagcaacagaccgtggtggcgattgatctggcgggcgatg<br>aaaccattccgggcagcagcctgctgccgggccatgtgcaagcgtatcaagaagcggtgaa<br>aagcggcattcatcgcaccgtgcatgcgggcgaagtgggcagcgcggaagtggtgaaaga<br>agcggtggatattctgaaaaccgaacgcctgggtcatggctatcataccctggaagatcaagc<br>gctgtataaccgcctgcgccaagaaaacatgcattttgaaatttgcccgtggagcagctatctg<br>accggcgcgtggaaaccggataccgaacatgcggtgattcgcctgaaaaacgatcaagcga<br>actatagcctgaacaccgatgatccgctgatttttaaaagcaccctggataccgattatcagatg<br>accaaacgcgatatgggctttaccgaagaggaatttaaacgcctgaacattaacgcggcgaa<br>aagcagctttctgccggaagatgaaaaacgcgaactgctggatctgctgtataaagcgtatgg	Full sequence of pSYSU-lac. Black indicates plasmid backbone, purple indicates Axe/Txe plasmid stabilization system, grey indicates lacUV5 promoter, pink indicates hsADA sequence, green indicates myc-tag sequence, blue indicates AIDA-I sequence, orange indicates PUC19 backbone.

catgccgccgagcgcgagcgcgggtcagaacctggagctcgaaaacctgtacttccagggt<br>gaacagaaactgattagcgaagaagatctgtctagagtgaataacaatggaagcattgtcatta<br>ataacagcattataaacgggaatattacgaatgatgctgacttaagttttggtacagcaaagctg<br>ctctctgctacagtgaatggtagtcttgttaataacaaaaatatcattcttaatcctacaaaagaaa<br>gtgcggccgctataggtaatactcttaccgtgtcaaattatactgggacaccgggaagtgttatt<br>tctcttggtggtgtgcttgaaggagataattcacttacggaccgtctggtggtgaaaggtaatac<br>ctctggtcaaagtgacatcgtttatgtcaatgaagatggcagtggtggtcagacgagagatggt<br>attaatattatttctgtagagggaaattctgatgcagaattctctctgaagaaccgcgtagttgcc<br>ggagcttatgattacacactgcagaaaggaaacgagagtgggacagataataagggatggta<br>tttaaccagtcatcttcccacatctgatacccggcaatacagaccggagaacggaagttatgct<br>accaatatggcactggctaactcactgttcctcatggatttgaatgagcgtaagcaattcaggg<br>ccatgagtgataatacacagcctgagtctgcatccgtgtggatgaagatcactggaggaataa<br>gctctggtaagctgaatgacgggcaaaataaaacaacaaccaatcagtttatcaatcagctcg<br>gggggatatttataaattccatgctgaacaactgggtgattttaccttagggattatgggagga<br>tacgcgaatgcaaaaggtaaaacgataaattacacgagcaacaaagctgccagaaacacact<br>ggatggttattctgtcggggtatacggtacgtggtatcagaatggggaaaatgcaacagggct<br>ctttgctgaaacttggatgcaatataactggtttaatgcatcagtgaaaggtgacggactggaa<br>gaagaaaaatataatctgaatggtttaaccgcttctgcaggtgggggatataacctgaatgtgc<br>acacatggacatcacctgaaggaataacaggtgaattctggttacagcctcatttgcaggctgt<br>ctggatggggttacaccggatacacatcaggaggataacggaacggtggtgcagggagc<br>agggaaaaataatattcagacaaaagcaggtattcgtgcatcctggaaggtgaaaagcaccct<br>ggataaggataccgggcggaggttccgtccgtatatagaggcaaactggatccataacactc<br>atgaatttggtgttaaaatgagtgatgacagccagttgttgtcaggtagccgaaatcagggaga<br>gataaagacaggtattgaaggggtgattactcaaaacttgtcagtgaatggcggagtcgcatat<br>caggcaggaggtcacgggagcaatgccatctccggagcactggggataaaatacagcttct<br>gataatgatcctggcacgcggcgcgccccttggtgcgcaaactattaactggcgaactactta<br>ctctagcttcccggcggttaatgtcatgataataatggtttcttagacgtcaggtggcacttttcg<br>gggaaatgtgcgcggaacccctatttgtttatttttctaaatacattcaaatatgtatccgctcatg<br>agacaataaccctgataaatgcttcaataatattgaaaaaggaagagtatgagtattcaacattt<br>ccgtgtcgcccttattcccttttttgcggcattttgccttcctgtttttgctcacccagaaacgctgg<br>tgaaagtaaaagatgctgaagatcagttgggtgcacgagtgggttacatcgaactggatctca<br>acagcggtaagatccttgagagttttcgccccgaagaacgttttccaatgatgagcacttttaaa<br>gttctgctatgtggcgcggtattatcccgtattgacgccgggcaagagcaactcggtcgccgc<br>atacactattctcagaatgacttggttgagtactcaccagtcacagaaaagcatcttacggatgg<br>catgacagtaagagaattatgcagtgctgccataaccatgagtgataacactgcggccaactt<br>acttctgacaacgatcggaggaccgaaggagctaaccgcttttttgcacaacatgggggatca<br>tgtaactcgccttgatcgttgggaaccggagctgaatgaagccataccaaacgacgagcgtg<br>acaccacgatgcctgtagcaatggcaacaacgttgcgcaaactattaactggcgaactactta<br>ctctagcttcccggcaacaattaatagactggatggaggcggataaagttgcaggaccacttct<br>gcgctcggcccttccggctggctggtttattgctgataaatctggagccggtgagcgtgggtct<br>cgcggtatcattgcagcactggggccagatggtaagccctcccgtatcgtagttatctacacg<br>acggggagtcaggcaactatggatgaacgaaatagacagatcgctgagataggtgcctcact<br>gattaagcattggtaactgtcagaccaagtttactcatatatactttagattgatttaaaacttcattt<br>ttaatttaaaaggatctaggtgaagatcctttttgataatctcatgaccaaaatcccttaacgtgag<br>ttttcgttccactgagcgtcagaccccgtagaaaagatcaaaggatcttcttgagatcctttttttc<br>tgcgcgtaatctgctgcttgcaaacaaaaaaaccaccgctaccagcggtggtttgtttgccgga<br>tcaagagctaccaactctttttccgaaggtaactggcttcagcagagcgcagataccaaatact<br>gttcttctagtgtagccgtagttaggccaccacttcaagaactctgtagcaccgcctacatacct<br>cgctctgctaatcctgttaccagtggctgctgccagtggcgataagtcgtgtcttaccgggttgg<br>actcaagacgatagttaccggataaggcgcagcggtcgggctgaacggggggttcgtgcac	

acagcccagcttggagcgaacgacctacaccgaactgagatacctacagcgtgagctatga<br>gaaagcgccacgcttcccgaagggagaaaggcggacaggtatccggtaagcggcagggt<br>cggaacaggagagcgcacgagggagcttccagggggaaacgcctggtatctttatagtcct<br>gtcgggtttcgccacctctgacttgagcgtcgatttttgtgatgctcgtcaggggggcggagcc<br>tatggaaaaacgccagcaacgcggcctttttacggttcctggccttttgctggccttttgctcaca<br>tgttctttcctgcgttat	
cgatactctctcctttcatcgaaataaccctatgaaaaatatggctgcaatcatcttgaatagtag<br>actcacaagggctaatacaaagcgccaaaagaccaaacaaaataccagtattgccaaaaata<br>ctacgaagtttaaatgcacagctgcagcatggcgcataagtatttcgccaacaaataggactg<br>acaatagaggaatgattttattcatatggatctccttctttctcttaggttatcgggtgttgcccgctt<br>atttcgcaccacctaagcggcgggggcggttggagcgattggtgcgaaataagaaccattcc<br>cttagttactaggtttttttgtccttatttcacacattctcacttttcggtataaatgattattgcctttttc<br>ctctcaaatcgttgatatggcattcgtttacggtcaagttcaggatccataagcgactaatgcca<br>attgcaataggaaataaacggttacccgttacgttggatgaaaagagacaaaaagaattgcag<br>caactaaagcagaagtacggcaaaagtgaatccaggattatgtgtattgcgttagatttattgatt<br>gcccaagaaaaagcaggatttgaggtaccagcactcaaaaagtgacgtcaccttttatcctaa<br>aaactaaaagtgatagcacttttaattataagaagttagaatattaatcatttgcttaattgtacaat<br>ataatgtacaattgttttatagaaataaataaggggtgaaaggaatggaagcagtagcttattca<br>aatttccgccaaaatttacgtagttatatgaaacaagttaatgaggatgctgaaacacttattgta<br>acaagtaaagatgtagaagatacagttgttgtattatcaaaaagagattatgattctatgcaaga<br>aacgttgagaacactttctaataattacgtcatggaaaaaattcgtcgaggagatgaacaattct<br>ccaaaggtgcatttaaaacacatgacttaatcgaggttgaatctgatgattaaggcttggtctgat<br>gatgcttgggatgattatctttattggcatgagcaaggaaacaaaagcaatataaaaaagattaa<br>caagttaataaaagatatcgatcgttcccccctttgctggattaggaaaacctgagccattaaagc<br>atgatttatctggaaaatggtccagaagaattacagatgaacatagactgatatatagagttgaa<br>aatgaaacgatatttatttattctgcaaaagatcactattaaccaatcggaagtaaggaaagggt<br>cagaaacttaaaagtttttgatccttattttatttaccctagtcatttaaaaagctaatatagcttagt<br>gttgattgttattaatgaatgtgtttgttacgcgtattacggatataaggttagtaaaatcatttctaa<br>agttgaggaaaagtaaatataaatggcttaaatttcaacaatttgaagttgaatagatatgttataa<br>tactattgtagtgtgggatgttagttactaaaggatgacgcttatatatatgactgaatagaataag<br>caataggtttaataatctattttaaatttttgtactagttttagtcaattagcaaaaacaacaaaaat<br>aaacttctcatagaatttagctaaaaattaatgatttatttacatattaaatttggatacagttaagta<br>atttttatatattggaggagaagtaatggaatataaatttaacttgaatttgaaagaagtatcgctg<br>ataccatggagttgttcttattggtggtgttgctttatggttgcatcgtagtaaatggttgtaacaaa<br>agcaatttttccggctgtctgtatacaaaaacgccgcaaagtttgagcgaagtcaataaactctc<br>tacccattcagggcaatatctctcttgaagaagcgatgcctaagcttcatatgaataaggcctac<br>agtatcatttggagccactccagacaggcctggattgtggcctcagagttagccagaggacat<br>ggttttgtccttgcaaaaaatacactgctggtattggcggttgtttccacaatcggaaatgcatttg<br>cagtcgacatggcgcagaccccggcgtttgataaaccgaaagtggaactgcatgtgcatctg<br>gatggcagcattaaaccggaaaccattctgtattatggccgccgtcgcggcattgcgctgccg<br>gcgaacaccgcggaaggcctgctgaacgtgattggcatggataaaccgctgaccctgccgg<br>attttctggcgaaatttgattattatatgccggcgattgcgggctgccgcgaagcgattaaacgc<br>attgcgtatgaatttgtggaaatgaaagcgaaagaaggcgtggtgtatgtggaagtgcgctata<br>gcccgcatctgctggcgaacagcaaagtggaaccgattccgtggaaccaagcggaaggcg<br>atctgaccccggatgaagtggtggcgctggtgggccaaggcctgcaagaaggcgaacgcg<br>attttggcgtgaaagcgcgcagcattctgtgctgcatgcgccatcagccgaactggagcccg<br>aaagtggtggaactgtgcaaaaaatatcagcaacagaccgtggtggcgattgatctggcggg<br>cgatgaaaccattccgggcagcagcctgctgccgggccatgtgcaagcgtatcaagaagcg	Full sequence of pSYSU-fnrs. Black indicates plasmid backbone, purple indicates Axe/Txe plasmid stabilization system, red indicates fnrs promoter, pink indicates hsADA sequence, green indicates myc-tag sequence, blue indicates AIDA-I sequence, orange indicates PUC19 backbone.

gtgaaaagcggcattcatcgcaccgtgcatgcgggcgaagtgggcagcgcggaagtggtg<br>aaagaagcggtggatattctgaaaaccgaacgcctgggtcatggctatcataccctggaagat<br>caagcgctgtataaccgcctgcgccaagaaaacatgcattttgaaatttgcccgtggagcagc<br>tatctgaccggcgcgtggaaaccggataccgaacatgcggtgattcgcctgaaaaacgatca<br>agcgaactatagcctgaacaccgatgatccgctgatttttaaaagcaccctggataccgattat<br>cagatgaccaaacgcgatatgggctttaccgaagaggaatttaaacgcctgaacattaacgcg<br>gcgaaaagcagctttctgccggaagatgaaaaacgcgaactgctggatctgctgtataaagc<br>gtatggcatgccgccgagcgcgagcgcgggtcagaacctggagctcgaaaacctgtacttc<br>cagggtgaacagaaactgattagcgaagaagatctgtctagagtgaataacaatggaagcatt<br>gtcattaataacagcattataaacgggaatattacgaatgatgctgacttaagttttggtacagca<br>aagctgctctctgctacagtgaatggtagtcttgttaataacaaaaatatcattcttaatcctacaa<br>aagaaagtgcggccgctataggtaatactcttaccgtgtcaaattatactgggacaccgggaa<br>gtgttatttctcttggtggtgtgcttgaaggagataattcacttacggaccgtctggtggtgaaag<br>gtaatacctctggtcaaagtgacatcgtttatgtcaatgaagatggcagtggtggtcagacgag<br>agatggtattaatattatttctgtagagggaaattctgatgcagaattctctctgaagaaccgcgt<br>agttgccggagcttatgattacacactgcagaaaggaaacgagagtgggacagataataagg<br>gatggtatttaaccagtcatcttcccacatctgatacccggcaatacagaccggagaacggaa<br>gttatgctaccaatatggcactggctaactcactgttcctcatggatttgaatgagcgtaagcaat<br>tcagggccatgagtgataatacacagcctgagtctgcatccgtgtggatgaagatcactggag<br>gaataagctctggtaagctgaatgacgggcaaaataaaacaacaaccaatcagtttatcaatc<br>agctcgggggggatatttataaattccatgctgaacaactgggtgattttaccttagggattatg<br>ggaggatacgcgaatgcaaaaggtaaaacgataaattacacgagcaacaaagctgccagaa<br>acacactggatggttattctgtcggggtatacggtacgtggtatcagaatggggaaaatgcaa<br>cagggctctttgctgaaacttggatgcaatataactggtttaatgcatcagtgaaaggtgacgg<br>actggaagaagaaaaatataatctgaatggtttaaccgcttctgcaggtgggggatataacctg<br>aatgtgcacacatggacatcacctgaaggaataacaggtgaattctggttacagcctcatttgc<br>aggctgtctggatgggggttacaccggatacacatcaggaggataacggaacggtggtgca<br>gggagcagggaaaaataatattcagacaaaagcaggtattcgtgcatcctggaaggtgaaaa<br>gcaccctggataaggataccgggcggaggttccgtccgtatatagaggcaaactggatccat<br>aacactcatgaatttggtgttaaaatgagtgatgacagccagttgttgtcaggtagccgaaatca<br>gggagagataaagacaggtattgaaggggtgattactcaaaacttgtcagtgaatggcggag<br>tcgcatatcaggcaggaggtcacgggagcaatgccatctccggagcactggggataaaata<br>cagcttctgataatgatcctggcacgcggcgcgccccttggtgcgcaaactattaactggcga<br>actacttactctagcttcccggcggttaatgtcatgataataatggtttcttagacgtcaggtggc<br>acttttcggggaaatgtgcgcggaacccctatttgtttatttttctaaatacattcaaatatgtatcc<br>gctcatgagacaataaccctgataaatgcttcaataatattgaaaaaggaagagtatgagtattc<br>aacatttccgtgtcgcccttattcccttttttgcggcattttgccttcctgtttttgctcacccagaaa<br>cgctggtgaaagtaaaagatgctgaagatcagttgggtgcacgagtgggttacatcgaactg<br>gatctcaacagcggtaagatccttgagagttttcgccccgaagaacgttttccaatgatgagca<br>cttttaaagttctgctatgtggcgcggtattatcccgtattgacgccgggcaagagcaactcggt<br>cgccgcatacactattctcagaatgacttggttgagtactcaccagtcacagaaaagcatcttac<br>ggatggcatgacagtaagagaattatgcagtgctgccataaccatgagtgataacactgcggc<br>caacttacttctgacaacgatcggaggaccgaaggagctaaccgcttttttgcacaacatggg<br>ggatcatgtaactcgccttgatcgttgggaaccggagctgaatgaagccataccaaacgacg<br>agcgtgacaccacgatgcctgtagcaatggcaacaacgttgcgcaaactattaactggcgaa<br>ctacttactctagcttcccggcaacaattaatagactggatggaggcggataaagttgcaggac<br>cacttctgcgctcggcccttccggctggctggtttattgctgataaatctggagccggtgagcgt<br>gggtctcgcggtatcattgcagcactggggccagatggtaagccctcccgtatcgtagttatct<br>acacgacggggagtcaggcaactatggatgaacgaaatagacagatcgctgagataggtgc<br>ctcactgattaagcattggtaactgtcagaccaagtttactcatatatactttagattgatttaaaac	

ttcatttttaatttaaaaggatctaggtgaagatcctttttgataatctcatgaccaaaatcccttaac<br>gtgagttttcgttccactgagcgtcagaccccgtagaaaagatcaaaggatcttcttgagatcct<br>ttttttctgcgcgtaatctgctgcttgcaaacaaaaaaaccaccgctaccagcggtggtttgtttg<br>ccggatcaagagctaccaactctttttccgaaggtaactggcttcagcagagcgcagatacca<br>aatactgttcttctagtgtagccgtagttaggccaccacttcaagaactctgtagcaccgcctac<br>atacctcgctctgctaatcctgttaccagtggctgctgccagtggcgataagtcgtgtcttaccg<br>ggttggactcaagacgatagttaccggataaggcgcagcggtcgggctgaacggggggttc<br>gtgcacacagcccagcttggagcgaacgacctacaccgaactgagatacctacagcgtgag<br>ctatgagaaagcgccacgcttcccgaagggagaaaggcggacaggtatccggtaagcggc<br>agggtcggaacaggagagcgcacgagggagcttccagggggaaacgcctggtatctttata<br>gtcctgtcgggtttcgccacctctgacttgagcgtcgatttttgtgatgctcgtcaggggggcgg<br>agcctatggaaaaacgccagcaacgcggcctttttacggttcctggccttttgctggccttttgc<br>tcacatgttctttcctgcgttat	

Supplementary Table. S3

Primers	Function	Sequence
Axe/Txe_fwd	Four sequences were seamlessly assembled by Gibson assembly method	cctgcgttatCGATACTCTCTCCTTTCATC
Axe/Txe_rev		tctgcgccatGTCGACTGCAAATGCATTTC
HsADA_fwd		tgcagtcgacATGGCGCAGACCCCGGCG
HsADA_rev		tttcgagctcCAGGTTCTGACCCGCGCTCG
AIDA1_fwd		tcagaacctgGAGCTCGAAAACCTGTAC
AIDA1_rev		gtctaagaaaCCATTATTATCATGACATTAACC
pUC19 backbone_fwd		ataataatggTTTCTTAGACGTCAGGTG
pUC19 backbone_rev		gagagtatcgATAACGCAGGAAAGAACATG

1 F. Tecles, C. P. Rubio, M. D. Contreras-Aguilar *et al.*, Adenosine deaminase activity in pig saliva: analytical validation of two spectrophotometric assays. *J. Vet. Diagn. Invest.* 2018; **30**: 175-79.

2 S. Freigang, F. Ampenberger, A. Weiss *et al.*, Fatty acid-induced mitochondrial uncoupling elicits inflammasome-independent IL-1α and sterile vascular inflammation in atherosclerosis. *Nat. Immunol.* 2013; **14**: 1045-53.

3 W. K. Lin, Y. Liu, J. H. Wang *et al.*, Engineered Bacteria Labeled with Iridium(III) Photosensitizers for Enhanced Photodynamic Immunotherapy of Solid Tumors. *Angew. Chem. Int. Ed. Engl.* 2023; **62**: e202310158.

4 Y. Tang, J. Xu, L. Chen *et al.*, Rapid in vivo determination of fluoroquinolones in cultured puffer fish (Takifugu obscurus) muscle by solid-phase microextraction coupled with liquid chromatography-tandem mass spectrometry. *Talanta* 2017; **175**: 550-56.

5 F. Ahmadi, C. Sparham, E. Boyacı *et al.*, Time Weighted Average Concentration Monitoring Based on Thin Film Solid Phase Microextraction. *Environ. Sci. Technol.* 2017; **51**: 3929-37.

6 Y. Hao, T. Stuart, M. H. Kowalski *et al.*, Dictionary learning for integrative, multimodal and scalable single-cell analysis. *Nat. Biotechnol.* 2024; **42**: 293-304.
